# miR-124-3p Suppresses the Invasiveness and Metastasis of Hepatocarcinoma Cells *via* Targeting CRKL

**DOI:** 10.3389/fmolb.2020.00223

**Published:** 2020-09-15

**Authors:** Abbasi Majid, Jinxia Wang, Muhammad Nawaz, Sattar Abdul, Munawar Ayesha, Chunmei Guo, Qinglong Liu, Shuqing Liu, Ming-Zhong Sun

**Affiliations:** ^1^Department of Biotechnology, College of Basic Medical Sciences, Dalian Medical University, Dalian, China; ^2^Department of Rheumatology and Inflammation Research, Institute of Medicine, Sahlgrenska Academy, University of Gothenburg, Gothenburg, Sweden; ^3^Department of Biochemistry, College of Basic Medical Sciences, Dalian Medical University, Dalian, China; ^4^Department of General Surgery, The Second Affiliated Hospital, Dalian Medical University, Dalian, China

**Keywords:** HCC, miR-124-3p, CRKL, proliferation, invasion, migration, EMT, apoptosis

## Abstract

Abnormal expressions of microRNAs are involved in growth and progression of human cancers including hepatocellular carcinoma (HCC). An adaptor protein CRKL plays a pivotal role in HCC growth, whereas miR-124-3p downregulation is associated with clinical stage and the poor survival of patients. However, the relationship between miR-124-3p and CRKL and the molecular mechanisms through which they regulate HCC metastasis remains unclear. In the current work, we explored miR-124-3p and its correlation with CRKL expression in HCC patient tissues. We found that miR-124-3p deficiency is inversely co-related with CRKL overexpression in tumorous tissues of HCC patients, which was also consistent in HCCLM3 and Huh7 HCC cell lines. Target validation data shows that miR-124-3p directly targets CRKL. The overexpression of miR-124-3p reverses the CRKL expression at both mRNA and protein levels and inhibits the cell development, migration, and invasion. Mechanistic investigations showed that CRKL downregulation suppresses the ERK pathway and EMT process, and concomitant decrease in invasion and metastasis of HCC cells. The expressions of key molecules in the ERK pathway such as RAF, MEK, ERK1/2, and pERK1/2 and key promoters of EMT such as *N*-cadherin and vimentin were downregulated, whereas *E*-cadherin, a key suppression indicator of EMT, was upregulated. MiR-124-3p-mediated CRKL suppression led to BAX/BCL-2 increase and C-JUN downregulation, which inhibited the cell proliferation and promoted the apoptosis in HCC cells. Collectively, our data illustrates that miR-124-3p acts as an important tumor-suppressive miRNA to suppress HCC carcinogenesis through targeting CRKL. The miR-124-3p-CRKL axial regulated pathway may offer valuable indications for cancer research, diagnosis, and treatment.

## Introduction

Hepatocellular carcinoma (HCC) is one of the most frequently occurring cancers and is the 5th most prevalent human malignancy globally (Forner et al., 2018). HCC develops in a complex multistep process together with modulations of extracellular matrix (ECM), translocation, intravasation, invasion, and migration, which ultimately form the metastatic niche (Liu et al., 2016; Zhou et al., 2016; Guo et al., 2018). Although great developments in the treatment of HCC have been made, 70% of HCC patients are unable to adapt to therapeutic actions like surgical resection and liver transplantation because of the difficulties in compatibility, tumor location, and size (Llovet et al., 2008; Liang et al., 2013). To control the progression and mortality of HCC mortality, new potential indicators together with their regulation mechanism are still highly required for its diagnosis and treatment.

MicroRNAs (miRNA, miRs) play essential roles in the origination and progression of HCC and exhibit applicable uses in the diagnosis and therapeutic action for the patients of HCC (Lee and Dutta, 2009; Li et al., 2017). miRNAs are non-coding regulatory RNA, of 18∼25 nucleotides in length, and are involved in the gene expression regulations at the posttranscriptional level through attaching at 3′-untranslated regions (3′-UTR) with their targeted mRNAs (Ambros, 2004; Bartel, 2009; Wang et al., 2016b; Guo et al., 2018). Studies have revealed that miRNAs play a critical part in the early stages and growth of tumors, by regulating biological processes like apoptosis, cell development, cellular proliferation, metastasis, differentiation, and metabolism (Bartel, 2009; Pan et al., 2010; Guo et al., 2018). Abnormal regulations of miRNAs in a variety of cancers revealed their roles both as tumor suppressor and as tumor promoter (Calin et al., 2004; Calin and Croce, 2006; Callegari et al., 2015; Guo et al., 2018; Luo et al., 2018).

Recent investigations have reported that the aberrant expression of miR-124-3p is associated with several cancers including bladder cancer (Yuan et al., 2017), breast cancer (Du et al., 2016), esophageal cancers (Zhang Y. et al., 2016), glioblastoma (Luo et al., 2018), and HCC (He et al., 2018; Long et al., 2018; Hu et al., 2019). In HCC, miR-124-3p downregulation has been related with clinical stage and the poor survival of patients (Long et al., 2018). Early study reported that miR-124-3p acted as a tumor suppressor by targeting ROCK2 and EZH2 genes (Zheng et al., 2012). MiR-124-3p also hinders the invasion in prostate cancer via blocking TGF-α-induced epithelial–mesenchymal transition (EMT) (Qin et al., 2014). Conversely, miR-124-3p downregulation was also found in various carcinomas. For instance, the reduced levels of miR-124-3p were associated with the poor prognosis of glioblastoma (Zhang Y. et al., 2016; Luo et al., 2018; Wu et al., 2018), whereas the restoration of miR-124-3p could prevent the malignant capacity of neoplastic cells (Cai et al., 2017).

V-crk sarcoma virus CT10 oncogene homolog (avian)-like (CRKL) is a CRK adapter protein family member, transversely expressed in eukaryotic organisms (Birge et al., 2009; Guo et al., 2014) and contributes in tumor growth and invasion. The overexpression of CRKL is involved in EMT, invasion, and apoptosis in various cancers (Cheung et al., 2011; Bell and Park, 2012). Studies have suggested that CRKL contributes pivotal roles in malignancy of human cancers such as glioblastoma (Lv et al., 2013), breast cancer (Zhao et al., 2013), and colon cancer (Lan et al., 2014), among others (Tsuda et al., 2004; Brabek et al., 2005; Mortazavi et al., 2011; Guo et al., 2014). Additionally, CRKL has been proposed as a possible candidate for cancer diagnosis and prognosis and as therapeutic target for certain cancers (Lin et al., 2015; Shi et al., 2015). High expression levels of CRKL have been shown to be strongly interrelated with decreased disease-free and overall survival in HCC patients, and CRKL was suggested as novel prognostic marker for HCC (Liu et al., 2013a,b). Hence, CRKL could play a role in cell signaling cascades via forming a complex immediately with the downstream receptor protein to regulate tyrosine kinase activity, or by performing a role as an upstream mediator for signal launch (Bell and Park, 2012; Park et al., 2016). The deregulations of CRKL have been correlated with development and progression of various cancers (Sriram and Birge, 2010; Shi et al., 2015). Our previous study stated that the knockdown and overexpression of CRKL significantly efficiently promote and suppress *in vitro* migration as well as invasion capabilities of HCC HepG2 cells (Guo et al., 2018). However, the regulation of CRKL by miR-124-3p has not been investigated so far. Moreover, the underlying molecular mechanisms through which miR-124-3p and CRKL interaction regulates the metastasis and invasion of HCC remain unclear.

In the current work, 23 frozen HCC human tissues including their corresponding non-tumor liver tissues were used for miR-124-3p. The CRKL protein expression analysis from these tissues was reported in our previous work (Guo et al., 2018, 2020). In the current study, we quantified miR-124-3p in these tissues and correlated with tissue expression of CRKL from a previous study. We also performed CRKL expression analysis in HCC cell lines *in vitro* and investigated their tumorigenesis activity. We predict and found that miR-124-3p binds to the 2283–2289 and 3785–3791 sites of *CRKL*-3′-UTR and negatively regulates the CRKL expression level in patient tissue samples and cells. Interestingly, clinical tissue samples confirmed downregulation of miR-124-3p, whereas the CRKL protein levels were found significantly higher. Moreover, the changes in their expression levels were negatively correlated in tumorous tissues from HCC patients and HCC cells. Besides that, miR-124-3p overexpression suppressed cell proliferation, migration, and invasion, as well as increased the apoptosis of HCCLM3 and Huh7 compared to normal cell line LO2. By directly downregulating the CRKL expression level, the miR-124-3p-CRKL axis mediated the malignant behaviors of HCC cancer cell lines through RAF/MEK/ERK1/2 pathways and EMT features. Collectively, our data discloses that miR-124-3p acts as an important tumor-suppressive miRNA through targeting and regulating CRKL in HCC. The miR-124-3p-CRKL axial regulated pathway may offer valuable indications for cancer research, diagnosis, and treatment.

## Materials and Methods

### Patients’ Tissue Samples

Twenty three frozen HCC human tissues including their corresponding non-tumor liver tissues (14 male and 9 female patients, 8 patients under ≥ 60 age and 15 < 60 age) were used for comparative miR-124-3p expression analysis. However, the CRKL protein expression analysis from these tissues was already reported in our previous work (Guo et al., 2018, 2020). In the current work, we quantified miR-124-3p in these tissues and correlated miR-124-3p expression from the current study, with CRKL expression from a previous study. The tissue samples were obtained from the Division of the Hepatobiliary and Pancreatic Surgery Department of Surgery, the Second Affiliated Hospital of Dalian Medical University, Dalian, China. No patient was treated with chemotherapy/radiotherapy prior to operation. Tissues were then instantly preserved at −196°C liquid nitrogen following resection. The Dalian Medical University Committee of Medical Ethics (ethical number 014, year 2019) has approved the study protocol and utilization of human tissues. All experiment methods were designed following the applicable rules and regulations.

### Cell Cultures

The human normal liver cells LO2 and HCC cells HCCLM3 with high metastasis and Huh7 with low metastasis were acquired from the Chinese Academy of Sciences (Shanghai, China). Cells were cultured with 90% Dulbecco’s Modified Eagle’s Medium (DMEM Gibco, United States) accompanied with 10% fetal bovine serum (FBS, TransGen, China), and double antibiotics 100 U/ml streptomycin and 100 U/ml penicillin (Gibco, United States) in 5% CO_2_ at 37°C.

### Cell Transfection

Cells in each group were digested using trypsin, transferred into a 15-ml falcon tube, then centrifuged for 5 min at 1000 rpm. Then, the supernatant was removed and the cell pellet was resuspended in fresh DMEM. 1 ml of HCCLM3 or Huh7 cells with the density of 2 × 10^5^/mL was transfected in each well of a six-well plate for 12 h to reach ∼75% confluence. Meanwhile, 100 μl transfection solution was prepared with 5 μl of miR-124-3p mimic or miR-Negative control (miR-NC) (Ribbo, China) with the concentration of 20 μM, 5 μl of Lipofectamine 2000 reagent (Invitrogen, United States) and 90 μl of serum-less medium, mixed well at RT for 20 min and added into each well cells for transfection. Incubation times for transfected cells were 24, 48, and 72 h individually at 37°C, 5% CO_2_.

### RNA Extraction and Quantitative RT-PCR

The total RNA was extracted using TRIzol Kit (Invitrogen, United States) conferring to the company’s guidelines. The concentration of RNA was measured by a NanoDrop 2000 spectrometer (Thermo, United States). The cDNA was produced through reverse transcription using PrimeScript (Takara, Japan). qRT-PCR was achieved on a 7300-plus RT-PCR using ROX reagents (Roche, Germany) and FastStart SYBR Green. The internal references for miR-124-3p and *CRKL* were snRNA U6 and β-actin (*ACTB*), respectively. The 2^−ΔCT^ method was used for analyzing relative levels of expression of miR-124-3p in tumorous versus paracancerous non-tumorous tissues, and negative correlations were analyzed by spearman correlation test. Primer sequences designed for miR-124-3p, *CRKL*, *ACTB*, and U6 are provided in Table 1.

**TABLE 1:** Primer sequences use in this study.

Gene	Primers	Sequences
miR-124-3p	Forward	5′-TCTTTAAGGCACGCGGTG-3′
	Reverse	5′-TATGGTTTTGACGACTGTGTGAT-3′
U6	Forward	5′-CTCGCTTCGGCAGCACA-3′
	Reverse	5′-AACGCTTCACGAATTTGCGT-3′
CRKL	Forward	5′-GTGCTTATGACAAGACTGCCT-3′
	Reverse	5′-CACTCGTTTTCATCTGGGTTT-3′
ACTB	Forward	5′-AGGCCAACCGCGAGAAG-3′
	Reverse	5′-ACAGCCTGGATAGCAACGTACA-3′

### Western Blotting Analysis

The extraction of total proteins from different groups of tissues and HCC cells through radioimmunoprecipitation assay (RIPA) solution (0.5%, 0.1% SDS, sodium deoxycholate, 1% Triton X-100, 150 mM NaCl, 50 mM pH 8.0 Tris–HCl with 0.5 mM PMSF, 1 mM Na_3_VO_4_, and 1 μg/ml leupeptin). The lysates were centrifuged at 12000 rpm for 15 min at 4°C, and the supernatants (containing proteins) were collected. The concentrations of total proteins in each sample were measured by Bradford assay. The equal amount of protein (35 μg) was separately taken from all samples, boiled in loading buffer for 5 min, and isolated through 10% sodium dodecyl sulfate-polyacrylamide gel electrophoresis followed by electrophoretic transfer of the protein band into a nitrocellulose membrane (NC) (PALL, United States). Non-specific binding was blocked with TBST (0.1% Tween-20, 100 mM NaCl, and 50 mM Tris and at pH 7.5) with skim milk (5% w/v) (BD, United States) for 2 h at room temperature (RT). Then, NC membranes were incubated in primary antibodies at 4°C for overnight.

The primary antibodies with their dilution factors include CRKL (1:2000, Cat# GTX107677, United States), RAF (1:500, Thermo Scientific Cat# PA5-37713, USA Invitrogen), p-MEK1/2 (1:500, Cat# ABP50356 pSer218/222, Abbkine, China), ERK1/2 (1:1000, Cell Signaling Technology Cat# 9102, United States), p-ERK1/2 (1:500, Cell Signaling Technology Cat# 9106), C-JUN (1:500, Cat# ABP57278, Abbkine, China), BCL-2 (1:1000, Proteintech Cat# 12789-1-AP), BAX (1:1000, Proteintech Cat# 50599-2-Ig), vimentin (1:1000, Proteintech Cat# 10366-1-AP), *N*-cadherin (1:1000, Proteintech Cat# 22018-1-AP), *E*-cadherin (1:1000, Proteintech Cat# 20874-1-AP), ACTB (1:4000, Proteintech Cat# 66009-1-Ig), GAPDH (1:4000, Proteintech Cat# 10494-1-AP), and HRP-conjugated AffiniPure Goat Anti-Rabbit IgG (1:4000, Proteintech Cat# SA00001-2). After washing three times for 10 min with TBST, the membranes were incubated for 2 h at RT in a secondary antibody. The incubation membrane was washed again three times for 10 min. The enhanced chemiluminescence (ECL) detector (Advansta, United States) was used for visualizing protein bands, and protein bands were analyzed by Bio-Rad ChemiDoc MP system. The analyses of variance between groups were performed using unpaired Student *t*-test and ANOVA on triplicate experiments.

### Wound Healing Assay

After the transfections with miR-NC and miR-124-3p mimic, the HCCLM3 and Huh7 cells with a density of 2 × 10^5^ cells/per ml were propagated into six-well plates and cultured to 90% confluency. The vertical scratches were created on a cell monolayer with a 200-μl sterilized tip. Separated and floated cells were gently discarded and washed away using 1 ml PBS, and each well was poured with 2 ml of fresh DMEM media. The wound-recovered gap was captured under a microscope (Olympus, Japan) with magnification 4 × at time intermissions of 0, 24, 48, and 72 h, and the scratched area was measured using ImageJ software. The data was presented as mean ± SD of triplicate biological experiments. Unpaired Student *t*-tests were applied between NC and miR-124-3p mimic group cells.

### Cell Proliferation Assay

To investigate cell proliferation, MTT assay was performed. Twenty four hour post-transfections with miR-NC/miR-124-3p mimics, each group was set to 3000 cells per well with 100 μl 10% FBS DMEM medium in a 96-well plate and incubated at 37°C with 5% CO_2_ for 24, 48, 72, and 96 h independently. Cells of each well were treated with MTT reagent (5 mg/ml) according to the manufacturer’s protocol (Coolaber, Beijing, China) for 4 h in the dark in an incubator. Then, MTT was exchanged with 150 μl DMSO to diffuse crystals of formazan. Then, absorbance was measured at 492 nm by a microplate reader (Thermo Scientific). Unpaired Student *t*-tests were applied for addressing the difference between NC and miR-124-3p mimic group cells. A graph was plotted between absorbance values versus time.

### Clonogenic Assay

The colony-forming capacity of HCCLM3 and Huh7 cells was quantified using plate clone formation assay. Following the 24-h transfections of miR-NC/miR-124-3p mimics of HCCLM3 and Huh7, 1000 cells were taken from each group and inoculated in 2 ml of 10% DMEM into a six-well plate following incubation of 12 days in CO_2_ at 37°C until clusters were visible. PBS was used to wash colonies, and 0.5% crystal violet was used for staining after fixing with absolute methanol. The stained colonies were photographed and calculated. All experiments were performed three times independently for the cells in each group. The data were expressed as mean ± SD, and the difference was analyzed by unpaired Student *t*-test.

### *In vitro* Cell Migration Assay

Cell migration capabilities of HCCLM3 and Huh7 cells were investigated by transwell chamber assay. Concisely, 1 × 10^4^ cells from each group were seeded in chambers using 8-μm polycarbonate filters (Corning, United States) with 200 μl FBS-free DMEM in a 24-well plate. Subsequently, 600 μl of 20% medium, acting as the cell’s chemoattractant, was added into the bottom of the chambers in 24-well plates following the incubation at 37°C with 5% CO_2_ for 24 h. Excessive cells were discarded from the bottom of chambers with cotton wipes, and the cells that migrated to the bottommost were fixed with absolute methanol and stained with crystal violet (0.5%). Five random fields were selected at the chamber’s lower side to capture images using an upright light microscope with 10 × magnification. The difference from triplicate experiments between NC group and miR-124 mimic group cells was statistically processed using the unpaired Student *t*-test analysis.

### *In vitro* Cell Invasion Assay

Extracellular matrix (ECM) 50-μl gels (1:7 dilution in DMEM, Sigma, United States) were used to pre-coat the transwell chamber filters followed by 30 min of incubation at 37°C with 5% CO_2_. The rest of the steps were followed by cell migration assay as described above.

### Immunofluorescence Assay

After 24 h of transfection with miR-NC/miR-124-3p mimics, cells were washed and fixed in the presence of 4% paraformaldehyde (PFA), for 20 min at RT. Each group contained 2 × 10^4^ cells/ml with 10% FBS DMEM in a six-well plate. 0.3% bovine serum albumin (BSA, Sigma-Aldrich, United States) was used to block non-specific binding for 1 h and incubated with CRKL antibody (1:500, GeneTex Cat# GTX107677, United States) overnight in the dark at 4°C. Excessive unbound antibodies were removed by PBS washing (three times) and then incubated with a secondary antibody in the dark for 1 h. HCCLM3 and Huh7 cells were stained with 4′,6-diamidino-2-phenylindole (DAPI) for 5 min in the dark at RT. Finally, the images were taken using an Olympus Bx3 upright fluorescence microscope.

### Luciferase Reporter Assay

#### Construction of Plasmid

The wild-type *CRKL*-3′-UTR fragment of 2187 base pairs was amplified by PCR that contains a pair of identified potentials of miR-124-3p binding sites at 2283–2289 and 3785–3791. The amplified products were implanted in a luciferase reporter gene at its downstream site in a firefly luciferase-expressing vector of psiCHECK-2, containing *Xho*I as well as a *Not*I rift site designated as psiCHECK-2-*CRKL*-3′-UTR-WT. The specific primers sequences were designed, for forward primer 5′-CTCGAGGCTCATAGTGAACACAGCAGACCTAGAAATGT AGC-3′ and for reverse primers 5′-GCGCGGCCGCCGGCT GATGCAAGTTTTATTGAGACAATAT-3′.

In contrast, the fragments of psiCHECK-2-*CRKL*-3′-UTR-WT1 (including 2283–2289 sites only) and psiCHECK-2-*CRKL*-3′-UTR-WT2 (including site 2 only) were also constructed. The 2283–2289 site forward and reverse primers were designed as 5′-CTCGAGATTAGCCCTTCCTTGCCAGTGAGAAC-3′ and 5′-GCGGCCGCAGTCCGTTAAGTTTTACTAGTTT-3′. The 3785–3791 site forward and reverse primers were 5′-CTCGAGGGGATGATGTGGTTTTTTGCCAGGTGTTTATA AT-3′ and 5′-GCGGCCGCCGGCTGATGCAAGTTTTATTGAG ACAATAT-3′. psiCHECK-2-*CRKL*-3′-UTR-MUT1 and MUT2 were constructed through site-directed mutagenesis by substituting the seed regions of CACGGAA to miR-124-3p. For psiCHECK-2-*CRKL*-3′-UTR-MUT1, two sets of primer sequences were designed. The sequences of forward and reverse primer 1 were 5′-CTCGAGATTAGCCCTTCCTTGCCAGTGAGAAC-3′ and 5′-GGTCGTGCCCTCCTTGAACTTCCGTGACATTCCTCCC-3′, and the sequences of forward and reverse primer 2 were 5′-CAGAGGGAGGAATGTACAGGAAGTTCAAGGAGGGCA CG-3′ and 5′-CTCGAGGGGATGATGTGGTTTTTTGCCAGG TGTTTATAAT-3′. The forward sequence for primer 3 was 5′-CTCGAGGGGATGATGTGGTTTTTTGCCAGGTGTTTAT AAT-3′, and that of reverse was 5′-GCGGCGGCGGAA TACAAAAATAAATTCCGTGTAATCTGTT-3′. The primer 4 forward sequence was 5′-CTTAACAGATT ACACGGAATTTATTTTTGTAT-3′ and that of the reverse sequence was 5′-CGGCTGATGCAAGTTTTATTGAGAC-3′ for psiCHECK-2-*CRKL*-3′-UTR-MUT2.

#### Luciferase Reporter Gene Assay

To detect the obligatory binding sites between miR-124-3p and *CRKL*, the luciferase reporter assay was performed. The constitutive firefly expression was provided with the psiCHECK-2 vector as an internal control. A 24-well-plate was used to seed 1 × 10^5^ cells in each well and incubated in 1 ml of 15% FBS DMEM medium with 5% CO_2_ at 37°C for 24 h. Co-transfection by miR-NC and miR-124-3p mimics was done in 293T cells in the presence of 1 μg of each psiCHECK-2-*CRKL*-3′-UTR-Wildtype, Wildtype1, Wildtype2, psiCHECK-2-*CRKL*-3′-UTR-MUT1, and Mutant2 correspondingly. Cells were collected from each well after 48 h of transfection and were washed with PBS and lysed in 100 μl passive lysis buffer (PLB) by gentle agitation for 15 min at RT. The luminometer tubes were loaded with lysates including 50 μl luciferase assay reagent II (LAR II to detect firefly potency) as well as 50 μl stop reagent (to detect luciferase potency). The potency or activity of luciferase was detected through a GloMax fluorescence reader (GloMax Multi Detection System). Unpaired Student t-test analysis was performed to measure the difference between groups.

### Data Presentation and Statistical Analysis

Three biological independent experiments were performed for every assay each with three replicates, and the data were presented as mean ± standard deviation of replicates. GraphPad Prism version 6 was used to create plots and graphs. The analysis of variances among groups was performed through unpaired Student *T*-test and ANOVA. The relationship of miR-124-3p with CRKL in HCC samples was analyzed via Spearman’s correlation, and the differences with *P* < 0.05 were considered significant.

## Results

### miR-124-3p Negatively Correlates With CRKL Expression in HCC Patient Tissues and Cells

**FIGURE 1:**
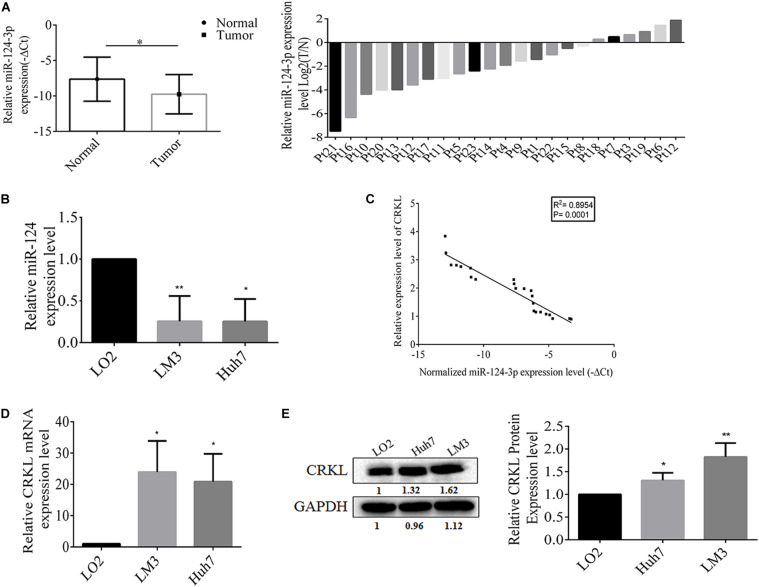
**(A)** The miR-124-3p level was downregulated in tumorous tissues with −ΔCT value of –9.7564 ± 2.7746 from HCC patients compared with that of –7.6357 ± 3.1093 from paracancerous normal liver tissue with statistical significance (*P* = 0.0188) by unpaired Student t-test analysis and presented as mean ± SD of 23 cases (*n* = 23). **(B)** Compared with LO2 cells, the miR-124-3p level was significantly reduced by 74% in HCCLM3 (*P* = 0.0130) and 75% in Huh7 cells (*P* = 0.0085) by unpaired Student *t*-test. **(C)** miR-124-3p downregulation and CRKL upregulation in HCC tumorous tissues were negatively correlated (*R*^2^ = 0.8954, *P* = 0.0001) by Spearman correlation test. **(D)** CRKL expressions at the mRNA level were increased by 23.844 ± 10.011 (*P* = 0.0164) and 20.821 ± 8.901 (*P* = 0.0178) folds, and **(E)** those at the protein level were elevated by 1.31 ± 0.165 (*P* = 0.0093) and 1.816 ± 0.321 (*P* = 0.0314) folds in HCCLM3 and Huh7 cells compared to LO2 cells. The data were presented as mean ± SD (standard deviation) of triplicate experiments for each assay. ^∗^, ^∗∗^, and ^∗∗∗^ refer to *P*-values below 0.05, 0.01, and 0.001.

First, the qRT-PCR assay showed that miR-124-3p relative level was downregulated at the −ΔCT level in HCC tumorous tissues compared with paracancerous normal tissue (*P* = 0.0188, Figure 1A). The miR-124-3p expression level was also significantly abridged by 74% in HCCLM3 (*P* = 0.0130) and 75% in Huh7 cells (*P* = 0.0085, Figure 1B) compared with normal liver LO2 cells. Our previous work showed the significant CRKL overexpression levels in the same cohort of HCC tumorous tissues as used in the present work (Guo et al., 2018, 2020). Our data analysis revealed a significantly negative correlationship between reduced miR-124-3p levels and overexpressed CRKL expression levels in tumorous tissues from HCC patients (*R*^2^ = 0.8954, *P* = 0.0001, Figure 1C).

Consistent with patient tissues, the CRKL levels were also elevated *in vitro*. Results show that *CRKL* mRNA levels in HCCLM3 were increased by 23.8-fold (*P* = 0.0164) and in Huh7 by 20.8-fold (*P* = 0.0178), respectively, compared to that in LO2 cells (Figure 1D). CRKL protein levels were increased 1.83-fold in HCCLM3 (*P* = 0.0093) and 1.31-fold in Huh7 cells (*P* = 0.0314) compared to LO2 cells (Figure 1E).

### miR-124-3p Suppresses CRKL Expressions in HCC Cells

**FIGURE 2:**
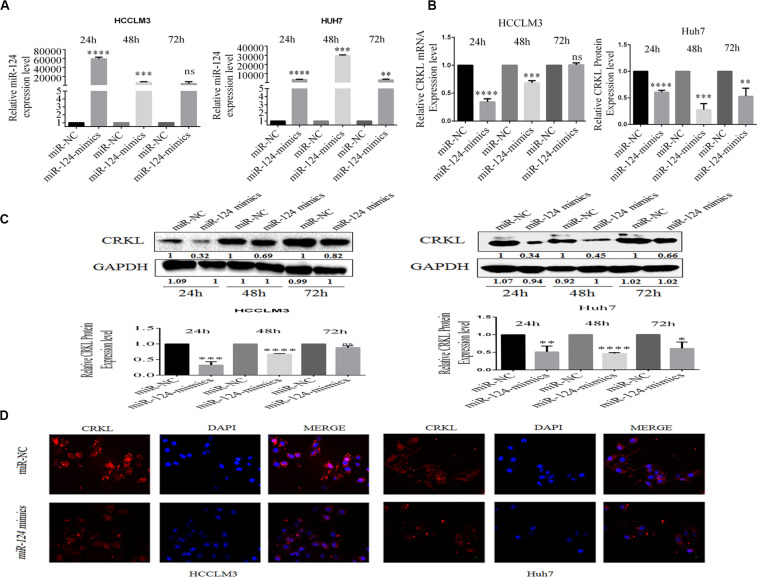
**(A)** miR-124-3p overexpression in HCCLM3 and Huh7 through miR-124-3p mimic transfections. **(B)** *CRKL* mRNA levels were suppressed in miR-124-3p-overexpressing HCCLM3 and Huh7 cells. **(C)** Western blotting assays of CRKL level changes in miR-124-3p-overexpressing HCCLM3 and Huh7 cells. **(D)** Immunofluorescence assay was executed to associate CRKL expression pattern changes in HCC cells LM3 and Huh7 following upregulated miR-124-3p by miR-124-p mimic transfection. The data of three independent experiments for each assay were shown as mean ± SD. Unpaired Student *t*-test analysis was performed to address the difference. ns refers to non-statistical significance. ^∗^, ^∗∗^, ^∗∗∗^, and ^****^ refer to *P*-values below 0.05, 0.01, 0.001, and 0.0001.

In order to further validate whether miR-124-3p deregulation affects CRKL expression, the transient transfection of miR-124-3p mimic was performed in HCCLM3 and Huh7 cell lines. At the transfection time points of 24, 48, and 72 h, miR-124-3p expression levels in HCCLM3 cells were increased by 59347- (*P* = 0.0001), 6699- (*P* = 0.0006), and 4363-fold (ns), and those in Huh7 cells were increased by 3292- (*P* = 0.0001), 29551- (*P* = 0.0001), and 2990-fold (*P* = 0.0042), respectively (Figure 2A). Additionally, the miR-124-3p overexpression affected the CRKL expression at the mRNA level, which was decreased by 65.56% (*P* = 0.00001), 31.8% (*P* = 0.0002), and non-significant in HCCLM3 cells and by 39.3% (*P* = 0.00001), 72.3% (*P* = 0.0004), and 47% (*P* = 0.0058) in Huh7 cells (Figure 2B) accordingly.

Consistent with mRNA levels of CRKL, miR-124-3p upregulation also resulted in decreases in CRKL expression at protein levels in HCCLM3 by 67.7% (*P* = 0.00001), 32.7% (*P* = 0.0002), and 11.7% (ns) and by 65.7% (*P* = 0.0001), 45.7% (*P* = 0.0018), and 39.4% (*P* = 0.0025) at 24, 48, and 72 h, respectively (Figure 2C). Moreover, compared with miR-NC-transfected group cells, the immunofluorescence staining assay for the identification of CRKL expression exhibited dull and poor CRKL expression patterns in HCCLM3 and Huh7 cells transfected with miR-124-3p mimics (Figure 2D).

### miR-124-3p Antagonistically Modulates CRKL Expression via Binding to Its 3′-UTR

**FIGURE 3:**
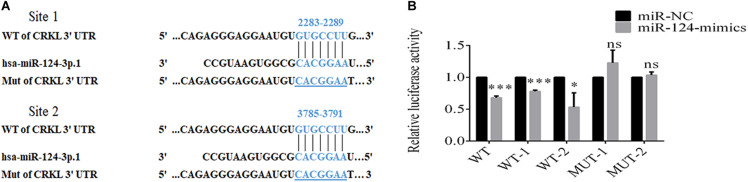
miR-124-3p binds to 3′-UTR of *CRKL*. **(A)** Putative sites for binding of *CRKL*-3′-UTR to miR-124-3p. **(B)** Dual luciferase activity assay of miR-124-3p binding to 3′-UTR of CRKL. The co-transfection of 293T cells was done through miR-NC/miR-124-3p mimics and psiCHECK-2-*CRKL*-3′-UTR-WT/psiCHECK-2-*CRKL*-3′-UTR-MUT for 48 h. The data of triplicate experiments were presented as mean ± SD and analyzed by unpaired Student t-test for statistical differences. ns refers to non-statistical significance. ^∗^ and ^∗∗∗^ refer to *P*-values below 0.05 and 0.001.

Bioinformatic analyses through TargetScan showed that *CRKL* was a potential target gene to miR-124-3p with two potential binding sites at 2283–2289 and 3785–3791 at *CRKL-*3′-UTR (Figure 3A). To validate the interaction between the miR-124-3p and *CRKL*-3′-UTR in 293T cells, luciferase reporter gene assay was performed. The two binding sites of miR-124-3p in *CRKL*-3′-UTR were cloned downstream of the firefly luciferase gene. The intensities of fluorescence were suppressed by 32.1 ± 1.8% (*P* = 0.0002), 22.1 ± 1.1% (*P* = 0.0001), and 46.8 ± 12.9% (*P* = 0.0226) in 293T cells co-transfected with the psiCHECK-2-*CRKL*-3′-UTR-WT, WT1, and WT2 plasmids and miR-124 mimic compared with miR-NC-transfected 293T cells co-transfected with psiCHECK-2-*CRKL*-3′-UTR-MUT1 and MUT2 (Figure 3B). This indicates that *CRKL* was a direct target of miR-124-3p.

### miR-124-3p Overexpression Suppressed the Growth of HCCLM3 and Huh7 Cells

**FIGURE 4:**
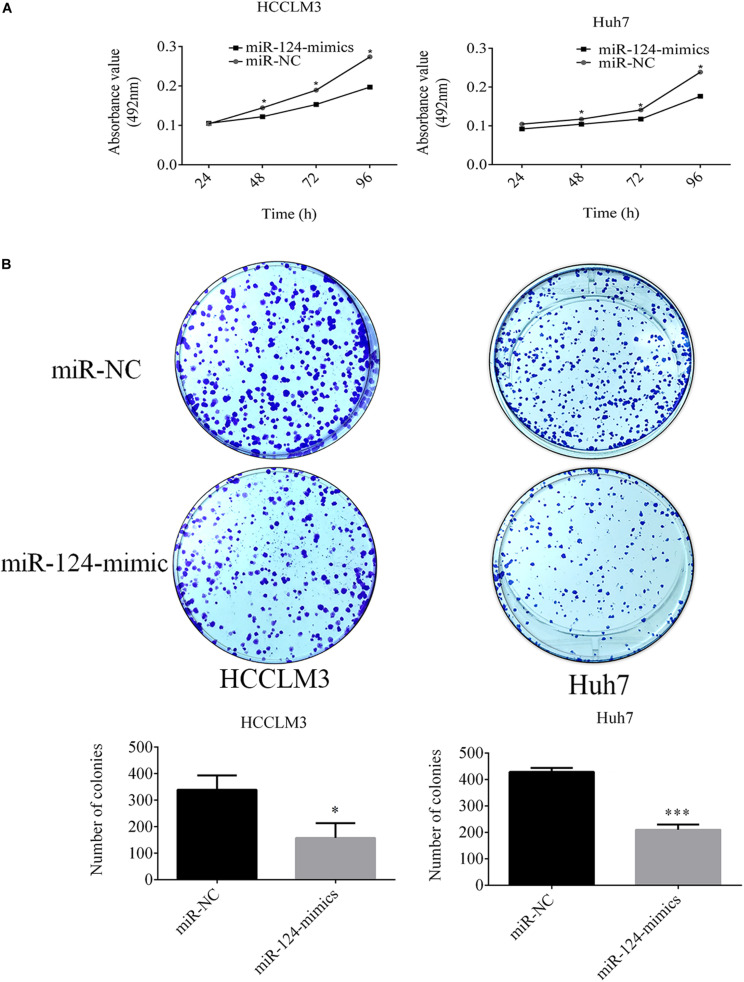
**(A)** miR-124-3p overexpression decreased the proliferation of HCCLM3 and Huh7. **(B)** Overexpressed miR-124-3p decreased comparative colony-forming capabilities and sizes of HCCLM3 and Huh7 cells. The data of three independent experiments were presented as mean ± SD and analyzed using unpaired Student *t*-test for statistical significances. ^∗^ and ^∗∗∗^ refer to *P*-values below 0.05 and 0.001.

miR-124-3p inhibited the proliferation of HCCLM3 and Huh7 cells. The MTT assay showed that proliferations of HCCLM3 cells transfected with miR-124 mimic were decreased by 15.58% (*P* = 0.0426), 21.95% (*P* = 0.0110), and 31.14% (*P* = 0.0227) at time intervals of 48, 72, and 96 h, respectively, compared with cells transfected by miR-NC. Similarly, the proliferation of miR-124 mimic-transfected Huh7 cells was decreased by 10.72% (*P* = 0.162), 11.45% (*P* = 0.0002), 17.02% (*P* = 0.0016), and 26.08% (*P* = 0.0004) at 24, 48, 72, and 96 h, respectively (Figure 4A).

Additionally, the CRKL expression level negatively mediated the colony-forming capabilities of HCCLM3 and Huh7 cells. Soft agar plate clone formation assay showed that the number of colonies formed by miR-124-3p mimic-transfected HCCLM3 was 158.66 ± 55.51 which was only about 46.44% of that (339.33 ± 53.72) of the miR-NC-transfected group (*P* = 0.0152, Figure 4B). Concordantly, the colony-forming ability in Huh7 cells was decreased through the miR-124-3p overexpression by 50.98% (colony number decreased from 429 ± 14.93 to 210.33 ± 20.03, *P* = 0.0001, Figure 4B). Moreover, overexpressed miR-124-3p decreased the colony size of HCCLM3 and Huh7.

### miR-124-3p Inhibited *in vitro* Migrations and Invasions of HCCLM3 and Huh7 Cells

**FIGURE 5:**
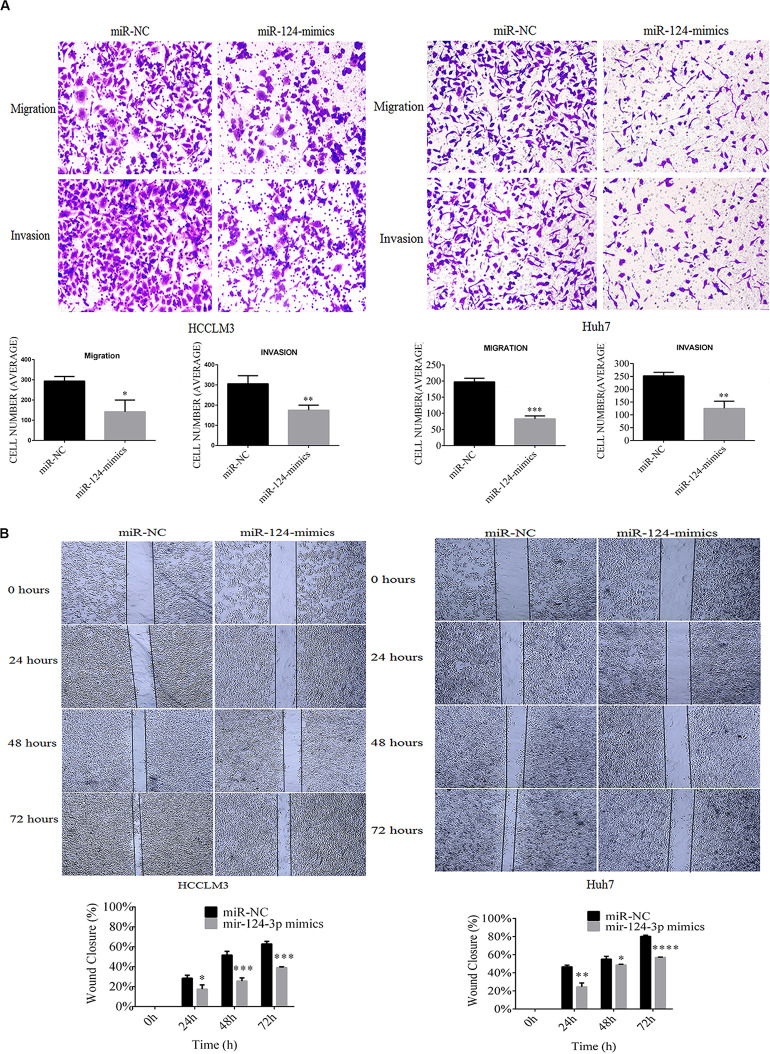
MiR-124-3p overexpression **(A)** decreased cell migration by 293.6 ± 23.2 to 141.6 ± 58.3 from triplicate biological assays (*P* = 0.0138) in HCCLM3 and 197.6 ± 11 to 83 ± 9 from triplicate biological assays (*P* = 0.0002) in Huh7, and decreased cell invasion by 305.6 ± 40 to 175 ± 25 from triplicate biological assays (*P* = 0.0087) in HCCLM3 and by 252.3 ± 13.6 to 125 ± 28.6 from 3 triplicate biological assays (*P* = 0.0022) in Huh7, as well as **(B)** decreased cell motilities by 0.283 ± 0.030 to 0.174 ± 0.042 (*P* = 0.0223), 0.515 ± 0.039 to 0.255 ± 0.032 (*P* = 0.0009), and 0.628 ± 0.025 to 0.370 ± 0.041 (*P* = 0.0008) at 24, 48, and 72 h in HCCLM3 and by 0.466 ± 0.018 to 0.245 ± 0.042 (*P* = 0.002), 0.551 ± 0.031 to 0.486 ± 0.0075 (*P* = 0.0265), and 0.802 ± 0.012 to 0.569 ± 0.004 (*P* = 0.0001) at 24, 48, and 72 for Huh7. Unpaired Student *t*-test analysis was performed for statistical significance. ^∗^, ^∗∗^,^***^, and ^****^ refer to *P* values below 0.05, 0.01, 0.001, and 0.0001.

In our recent work, CRKL overexpression positively related with migration and invasion abilities of HCCLM3 and Huh7 cells (Guo et al., 2020). After having confirmed the expression correlation and the target relation, we aimed to inspect the effect of miR-124-3p upregulation on cell migration and invasion abilities of HCCLM3 and Huh7 cells. Results showed that overexpressed miR-124-3p reduces the migration, invasion, and motility abilities of HCCLM3 and Huh7 cells. Due to miR-124-3p overexpression, the average quantity of cell migrated cells per field was decreased from 293.6 ± 23.2 to 141.6 ± 58.3 for HCCLM3 cells (*P* = 0.0138, Figure 5A) and from 197.6 ± 11 to 83 ± 9 for Huh7 cells (*P* = 0.0002, Figure 5A). Consistently, the mean number of invaded cells per field was decreased from 305.6 ± 40 to 175 ± 25 for HCCLM3 cells (*P* = 0.0087) and from 252.3 ± 13.6 to 125 ± 28.6 for Huh7 cells (*P* = 0.0022, Figure 5A). As miR-124-3p repressed the migration and invasion abilities of HCCLM3 and Huh7, our data suggested tumor-suppressor function of miR-124-3p in HCC tumorigenesis.

Moreover, overexpressed miR-124-3p reduces the motility capacities of HCCLM3 and Huh7 cells. As shown in Figure 5B, the wound healing assay indicated that wound closure percentages of miR-NC-transfected HCCLM3 cells were 30%, 52%, and 65% at the incubation time points of 24, 48, and 72 h, respectively, while those of miR-124-3p mimic-transfected HCCLM3 cells were 18% (*P* = 0.0223), 26% (*P* = 0.0009), and 38% (*P* = 0.0008). Consistently, the wound closure percentages of miR-NC-transfected Huh7 cells were measured as 47%, 56%, and 82% at the time intervals of 24, 48, and 72, while those of miR-124-3p mimic-transfected Huh7 cells were measured as 25% (*P* = 0.002), 48% (*P* = 0.0265), and 58% (*P* = 0.0001). The overexpression of miR-124-3p suppressed the motility abilities of HCC cell lines.

### miR-124-3p-CRKL Axial Regulation Mediates the Malignant Behaviors of HCC Cells via RAF/MEK/ERK and C-JUN Pathways, EMT Process, and Cell Apoptosis

**FIGURE 6:**
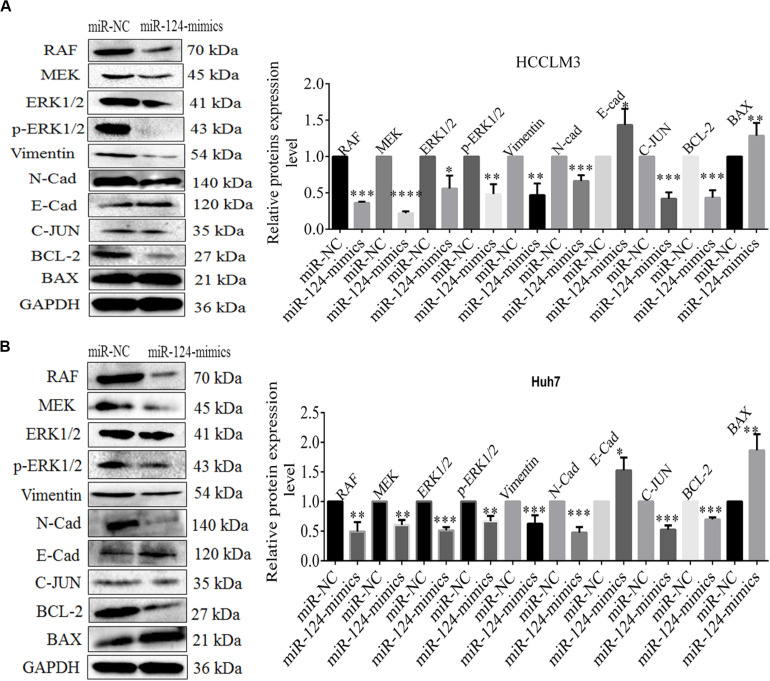
**(A)** The expression level changes of proteins RAF 0.363 ± 0.015 (*P* = 0.0001), MEK 0.220 ± 0.026 (*P* = 0.0001), ERK1/2 0.506 ± 0.177 (*P* = 0.0128), p-ERK1/2 0.483 ± 0.135 (*P* = 0.0027), vimentin 0.470 ± 0.160 (*P* = 0.0047), *N*-cadherin 0.626 ± 0.140 (*P* = 0.0008), *E*-cadherin 1.433 ± 0.223 (*P* = 0.0284), C-JUN 0.413 ± 0.075 (*P* = 0.0003), BCL-2 0.433 ± 0.102 (*P* = 0.0007), and BAX 1.286 ± 0.175 (*P* = 0.034) in miR-NC/miR-124-3p mimic-transfected HCCLM3 and **(B)** RAF 0.493 ± 0.158 (*P* = 0.0054), MEK 0.603 ± 0.085 (*P* = 0.0013), ERK1/2 0.513 ± 0.055 (*P* = 0.0001), p-ERK1/2 0.666 ± 0.090 (*P* = 0.0031), vimentin 0.626 ± 0.140 (*P* = 0.0099), *N*-cadherin 0.476 ± 0.092 (*P* = 0.0006), *E*-cadherin 1.526 ± 0.215 (*P* = 0.0133) C-JUN 0.526 ± 0.070 (*P* = 0.0003), BCL-2 0.696 ± 0.035 (*P* = 0.0001), and BAX 1.863 ± 0.273 (*P* = 0.0054) in Huh7. GAPDH was taken as the internal reference. The data was presented as mean ± SD of triplicate experiments for each protein. Unpaired Student *t*-test analysis was done for statistical significance. ^∗^, ^∗∗^, and ^∗∗∗^ refer to *P*-values below 0.05, 0.01, and 0.001.

The above evidences already proved that CRKL expression levels were inversely regulated by miR-124-3p through attaching with its 3′-UTR region. We then explored their axial regulation mechanism. Using Western blot assay, we found that the protein levels of RAF, MEK, ERK1/2, p-ERK1/2, C-JUN, BCL-2, vimentin, and *N*-cadherin in miR-124-3p-transfected HCCLM3 cells were significantly decreased by 64% (*P* = 0.0001), 78% (*P* = 0.0001), 44% (*P* = 0.0128), 52% (*P* = 0.0027), 59% (*P* = 0.0003), 57% (*P* = 0.0007), 53% (*P* = 0.0047), and 37.33% (*P* = 0.0008), compared with miR-NC-transfected cells. Likewise, the levels of BAX and *E-*cadherin were elevated by 1.29-fold (*P* = 0.034) and 1.43-fold (*P* = 0.0284) in miR-124-3p-overexpressing HCCLM3 cells via miR-124-3p mimic transfection (Figure 6A).

Concordantly, overexpressing miR-124-3p in Huh7 cells led to decreased expressions of RAF, MEK, ERK1/2, p-ERK1/2, C-JUN, BCL-2, vimentin, and N-cadherin with 51% (*P* = 0.0054), 40% (*P* = 0.0013), 49% (*P* = 0.0001), 33% (*P* = 0.0031), 47% (*P* = 0.0003), 30%(*P* = 0.0001), 37.3% (*P* = 0.0099), and 55% (*P* = 0.0006), whereas the expression level of BAX and E-cadherin increases to 1.86-fold (*P* = 0.0054) and 1.50-fold (*P* = 0.0133) in Huh7 cells (Figure 6B). The data indicates that the miR-124-3p-CRKL axis mediates the malignant properties of HCCLM3 and Huh7 *via* C-JUN in regulating cell proliferation, *via* RAF/MEK/ERK1/2 and Vimentin/*N*-cadherin/*E*-cadherin (EMT) regulating cell invasion and metastasis, and *via* BAX/BCL-2 by regulating cell apoptosis.

## Discussion

Hepatocellular carcinoma (HCC) is the commonly occurring cancer which ranks 5th among the most prevalent human malignancies in the world (Ren et al., 2017; Guo et al., 2018; Mahmoudvand et al., 2019). HCC is counted as a fundamental reason for cancer-related deaths globally. Studies revealed tumor-suppressor function of miR-124-3p in certain cancers including HCC (He et al., 2018; Long et al., 2018; Hu et al., 2019). MiR-124-3p has been shown to be vital for HDAC-mediated C-EBPα initiation in HCC (Hu et al., 2019) and may act as tumor-suppressor *via* targeting CBL in breast cancer (Wang et al., 2016a). More reports show that miR-124-3p reduces gastric cancer cell proliferation *via* targeting SPHK1 (Xia et al., 2012) and suppresses cervical cancer tumorigenesis through targeting AEG-1 (Zhang X. et al., 2016).

In HCC, miR-124-3p downregulation has been associated with clinical stage and poor survival patients (Long et al., 2018). However, the underlying molecular mechanism through which it regulates the metastasis and invasion of HCC remains unclear. Current work linked the downregulated miR-124-3p to CRKL with HCC progression using patient tissues and HCC cell lines. CRKL performs a vital function in the aggressive malignancy of human cancers and is pleiotropic in regulation of proliferation, adhesion, migration, invasion, and phagocytosis of cells (Tsuda et al., 2004; Brabek et al., 2005; Mortazavi et al., 2011; Guo et al., 2014). CRKL itself has been reported as a possible candidate for cancer diagnosis and prognosis and as a therapeutic target for certain cancers (Lin et al., 2015; Shi et al., 2015). Our recent study showed that CRKL is highly expressed in IHC of hepatocarcinoma patient tissue samples and HCC cells and is negatively regulated by miR-429 overexpression (Guo et al., 2018, 2020); we demonstrated that the CRKL protein level was overexpressed and miR-124-3p was downexpressed in tumorous tissues of HCC patients, which provided a negative correlation between CRKL and miR-124-3p alterations. Current findings proposed that miR-124-3p downregulation was probably a normal occurrence in HCC, which was consistent with a report showing that miR-124-3p suppressed migration and invasion of HCC cells via decreasing the expression of integrin alphaV (Cai et al., 2017). MiR-124-3p overexpression negatively regulates CRKL at mRNA and protein expression levels in HCCLM3 and Huh7. By directly targeting at *CRKL*’s 3′-UTR, miR-124-3p apparently suppressed *CRKL*/CRKL expression levels in HCC cells. These results suggested that direct binding of miR-124-3p with *CRKL* probably post-transcriptionally mediated the function of CRKL.

Additionally, miR-124-3p overexpression in HCC cells reduces their *in vitro* malignant capacities. The proliferation, colony formation, migration, invasion, and motility capacities of HCCLM3 and Huh7 cells were highly reduced due to miR-124-3p overexpression. Previous studies reported that CRKL enhances malignancy of the cancers such as glioblastoma (Lv et al., 2013), breast cancer (Zhao et al., 2013), and colon cancer (Lan et al., 2014). In liver cancer, protein–protein interface analysis proved CRKL as a unique marker for HCC, and analysis of the differential network biology resolved the innovative communication between CRKL-FLT1 which intensely correlated with the migrating capacity of HCC (Liu et al., 2013a,b). Our previous work also indicated that CRKL upregulation potentially promoted HCC progression by enhancing the *in vitro* malignant behaviors of HepG2 cells (Guo et al., 2018). The *in vitro* data on miR-124-3p and CRKL expression as well as invasion and migration activities in HCC cells from current study are consistent with our previous studies (Guo et al., 2018, 2020).

Besides these, also based on the evidence that CRKL was a straight downstream protein of miR-124-3p, the expression of CRKL was highly suppressed in HCC cells by miR-124-3p and the inverse correlation of miR-124-3p deficiency with CRKL upregulation in HCC tumorous tissues indicates that miR-124-3p together with its target binding with CRKL forms an important axis regulation mechanism in HCC malignancy.

**FIGURE 7:**
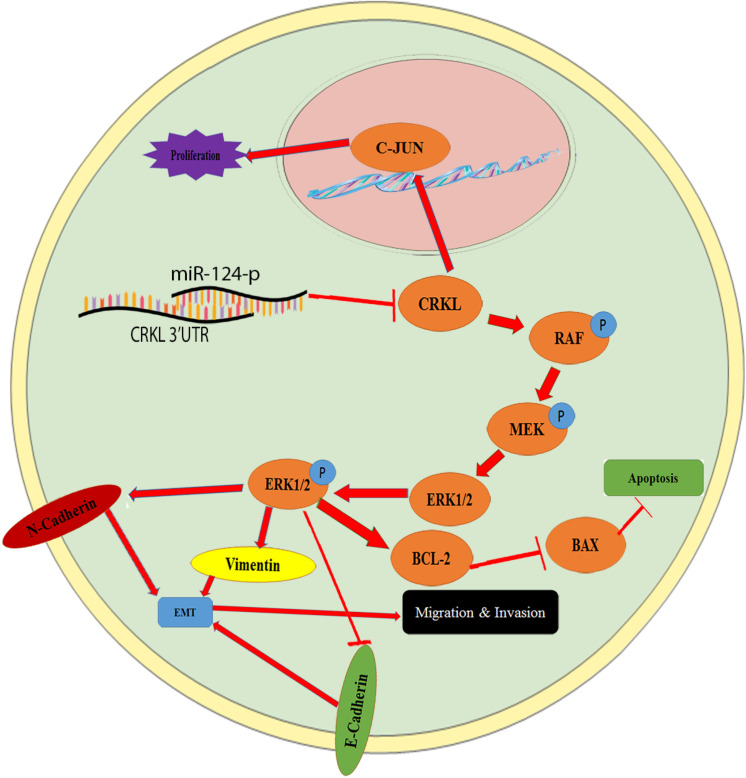
The schematic illustration of the miR-124-3p-CRKL axis in HCC malignancy. CRKL downregulation by miR-124-3p leads to suppressed C-JUN expression and decreased proliferations of HCC cells, whereas the CRKL suppression by miR-124-3p results in increased BAX levels and reduced BCL-2 and concomitant increased apoptosis of HCC cells. By binding to 3′-UTR of *CRKL*, miR-124-3p downregulates CRKL expression, which inhibits the activity of the RAF/MEK/ERK1/2 pathway and results in decreased EMT, ultimately leading to inhibited migration, invasion, and metastasis of HCC cells.

Current work also demonstrated that miR-124-3p and miR-124-3p-CRKL axis mediated HCC malignancy *via* the RAF-MEK-ERK pathway through regulating cancer migration and invasion, and *via N*-cadherin, *E*-cadherin, and vimentin, through regulation of EMT and *via* C-JUN and BAX/BCL-2 pathways by regulating cell proliferation and apoptosis, as schemed in Figure 7. RAS, RAF, MEK, ERK, and C-JUN cascades are vital courses for the integration of cell signaling pathway (Li et al., 2016). Epithelial–mesenchymal transition (EMT) is a central transformation course of tumor metastatic progression. Vimentin, *E*-cadherin, and *N*-cadherin are essential indicators of the cancer EMT process (Mathias and Simpson, 2009; Voulgari and Pintzas, 2009). The imbalance of expression ratio of Bax/Bcl-2 reveals the growth status of cancer cells (Rashmi et al., 2005; Yu and Zhang, 2005).

For the first time, current work revealed that the miR-124-3p-CRKL axis mediated HCC invasiveness and metastasis through the RAF-MEK-ERK pathway and EMT process (Figure 7). As a main subfamily of the MAPK pathway, the ERK pathway performs essential functions in cellular differentiation and metastasis, the most vital pathway for HCC progression and developments are the RAS/Raf/ERK pathway (Chen and Wang, 2015). Overexpression miR-124-3p notably abridged the expression level of proteins, RAF, MEK, ERK1/2, and p-ERK1/2 in HCCLM3 and Huh7 cells. In cancer progression, EMT performs important metastatic steps. As indicators for EMT, vimentin and *N*-cadherin expression levels were diminished; however, *E*-cadherin expression levels were improved in miR-124-3p-overexpressing HCCLM3 and Huh7 cells. By deactivating RAF-MEK-ERK pathway activity and suppressing EMT progress (Figure 7), miR-124-3p overexpression resulted in decreased metastasis and invasion capacities of HCC cells. The results mentioned above showed that miR-124-3p played a tumor-suppressive role and CRKL acting as a tumor promoter for HCC. It is implicated that the insufficiency of miR-124-3p inversely correlated with CRKL upregulation endorsed the invasiveness and metastasis of HCC cells and the clinical development and progression of HCC patients through activating the ERK pathway and EMT process (Figure 7).

Abnormal expression of miR-124-3p affected the proliferation and apoptosis of HCC cells. Following miR-124-3p overexpression, the C-JUN expression level was significantly reduced in HCCLM3 and Huh7, which led to their decreased cell proliferations and colony formation capacities. Meanwhile, miR-124-3p overexpression resulted in BAX upregulation and BCL-2 downregulation with BAX/BCL-2 ratios increased by ∼200% and ∼166% in HCCLM3 and Huh7 cells, which probably synergically led to their weakened growths by enhancing their apoptosis. Therefore, the insufficiency of miR-124-3p inversely correlated with CRKL upregulation might contribute to HCC clinical progression *via* elevated malignancy of HCC cancer cells through C-JUN, BAX, and BCL-2 deregulations.

More interestingly, the miR-124-3p-CRKL axis affected the apoptosis and growth of HCCLM3 and Huh7 cells through BAX/BCL-2 *via* the linkage from the RAF-MEK-ERK-pathway (Figure 7). RAF, MEK, ERK, and p-ERK1/2 were involved in the cell apoptosis process (McCubrey et al., 2007; Li et al., 2016). Via targeting phosphoinositide 3-kinase catalytic subunit alpha (PIK3CA), miR-124-3p overexpression noticeably repressed HepG2 cell proliferation through encouraging G1-phase cell-cycle arrest (Lang and Ling, 2012). Reported as an adaptor protein for RAS/MAPK, JAK/STAT, and PI3K pathways, CRKL led to the overexpression of apoptosis-inhibited gene BCL-2 in colon cancer (Lan et al., 2014). Current work indicated that targeting and suppressing CRKL overexpressed miR-124-3p in HCCLM3 and Huh7 cells, resulting in reduced RAF-MEK-ERK pathway activation, increased expression of pro-apoptotic protein (BAX), and suppressed expression of an anti-apoptotic protein (BCL-2) and conclusively promoting their apoptosis for weakened excessive growth (Figure 7).

## Conclusion

This work demonstrates that miR-124-3p deficiency negatively correlated with CRKL overexpression, which enhances the clinical progression of HCC patients and the malignancy of HCC cells. CRKL is a direct target gene of miR-124-3p. By binding to the 3′-UTR of *CRKL*, miR-124-3p suppresses CRKL expression in HCC cells and the malignant potentials of HCC cells. As illustrated in Figure 7, the miR-124-3p-CRKL axis mediates the invasive and metastatic capacities of HCCLM3 and Huh7 cells through C-JUN and RAF/MEK/ERK1/2-BAX/BCL-2 pathways (Figure 7) and EMT process through vimentin, *N*-cadherin, and *E-*cadherin. miR-124-3p-CRKL axial function could be of great importance in the fundamental research, clinical diagnosis, and treatment of liver cancer.

## Data Availability Statement

The raw data supporting the conclusions of this article will be made available by the authors, without undue reservation.

## Ethics Statement

The studies involving human participants were reviewed and approved by The Dalian Medical University Committee of Medical ethics ethical number 014, year 2019. The patients/participants provided their written informed consent to participate in this study.

## Author Contributions

AM designed the research, carried out the experiments, and wrote the first draft of the manuscript. MA helped in the experiments. CG helped in analyzing the data. QL provided the HCC tissue samples. AM, JW, MN, SA, MA, CG, QL, SL, and M-ZS contributed in manuscript writing. JW, MN, and SA helped in manuscript writing, editing, and critical evaluation. M-ZS supervised the project. M-ZS and SL read and approved the manuscript. All authors contributed to the article and approved the submitted version.

## Conflict of Interest

The authors declare that the research was conducted in the absence of any commercial or financial relationships that could be construed as a potential conflict of interest.

## References

[B1] Ambros V. (2004). The functions of animal microRNAs. *Nature* 431 350–355. 10.1038/nature02871 15372042

[B2] Bartel D. P. (2009). MicroRNAs: target recognition and regulatory functions. *Cell* 136 215–233. 10.1016/j.cell.2009.01.002 19167326 PMC3794896

[B3] Bell E. S. Park M. (2012). Models of crk adaptor proteins in cancer. *Genes Cancer* 3 341–352. 10.1177/1947601912459951 23226572 PMC3513787

[B4] Birge R. B. Kalodimos C. Inagaki F. Tanaka S. (2009). Crk and CrkL adaptor proteins: networks for physiological and pathological signaling. *Cell Commun. Signal.* 7:13. 10.1186/1478-811X-7-13 PMC2689226 19426560

[B5] Brabek J. Constancio S. S. Siesser P. F. Shin N. Y. Pozzi A. Hanks S. K. (2005). Crk-associated substrate tyrosine phosphorylation sites are critical for invasion and metastasis of SRC-transformed cells. *Mol. Cancer Res.* 3 307–315. 10.1158/1541-7786.mcr-05-0015 15972849

[B6] Cai Q. Dong Y. Wang R. Qi B. Guo J. Pan J. (2017). MiR-124 inhibits the migration and invasion of human hepatocellular carcinoma cells by suppressing integrin alphaV expression. *Sci. Rep.* 7:40733. 10.1038/srep40733 PMC5240551 28094803

[B7] Calin G. A. Croce C. M. (2006). MicroRNA signatures in human cancers. *Nat. Rev. Cancer* 6 857–866. 10.1038/nrc1997 17060945

[B8] Calin G. A. Sevignani C. Dumitru C. D. Hyslop T. Noch E. Yendamuri S. (2004). Human microRNA genes are frequently located at fragile sites and genomic regions involved in cancers. *PNAS* 101 2999–3004. 10.1073/pnas.0307323101 14973191 PMC365734

[B9] Callegari E. Gramantieri L. Domenicali M. D’Abundo L. Sabbioni S. Negrini M. (2015). MicroRNAs in liver cancer: a model for investigating pathogenesis and novel therapeutic approaches. *Cell Death Differ.* 22 46–57. 10.1038/cdd.2014.136 25190143 PMC4262781

[B10] Chen C. Wang G. (2015). Mechanisms of hepatocellular carcinoma and challenges and opportunities for molecular targeted therapy. *World J. Hepatol.* 7 1964–1970. 10.4254/wjh.v7.i15.1964 26244070 PMC4517155

[B11] Cheung H. W. Du J. Boehm J. S. He F. Weir B. A. Wang X. (2011). Amplification of CRKL induces transformation and epidermal growth factor receptor inhibitor resistance in human non-small cell lung cancers. *Cancer Discov.* 1 608–625. 10.1158/2159-8290.cd-11-0046 22586683 PMC3353720

[B12] Du S. Li H. Sun X. Li D. Yang Y. Tao Z. (2016). MicroRNA-124 inhibits cell proliferation and migration by regulating SNAI2 in breast cancer. *Oncol. Rep.* 36 3259–3266. 10.3892/or.2016.5163 27748910

[B13] Forner A. Reig M. Bruix J. (2018). Hepatocellular carcinoma. *Lancet* 391 1301–1314. 29307467 10.1016/S0140-6736(18)30010-2

[B14] Guo C. Gao C. Zhao D. Li J. Wang J. Sun X. (2020). A novel ETV6-miR-429-CRKL regulatory circuitry contributes to aggressiveness of hepatocellular carcinoma. *J. Exp. Clin. Cancer Res.* 39:170. 10.1186/s13046-020-01559-1 PMC7178969 32326970

[B15] Guo C. Liu S. Sun M.-Z. (2014). The role of CT10 regulation of kinase-like in cancer. *Future Oncol.* 10 2687–2697. 10.2217/fon.14.199 25531052

[B16] Guo C. Zhao D. Zhang Q. Liu S. Sun M.-Z. (2018). miR-429 suppresses tumor migration and invasion by targeting CRKL in hepatocellular carcinoma via inhibiting Raf/MEK/ERK pathway and epithelial-mesenchymal transition. *Sci. Rep.* 8:2375. 10.1038/s41598-018-20258-8 PMC5799248 29403024

[B17] He R. Yang X. Liang L. Chen G. Ma J. (2018). MicroRNA-124-3p expression and its prospective functional pathways in hepatocellular carcinoma: a quantitative polymerase chain reaction, gene expression omnibus and bioinformatics study. *Oncol. Lett.* 15 5517–5532. 29552191 10.3892/ol.2018.8045 PMC5840674

[B18] Hu X. Feng J. Huang X. Lu P. Wang Z. Dai H. (2019). Histone deacetylases up-regulate C/EBPalpha expression through reduction of miR-124-3p and miR-25 in hepatocellular carcinoma. *Biochem. Biophys. Res. Commun.* 514 1009–1016. 10.1016/j.bbrc.2019.05.024 31092334

[B19] Lan B. Zhang J. Shan J. Zhang P. Zhang W. Chen Y. (2014). Downregulation of CRKL expression can inhibit tumorigenesis in colon cancer. *Front. Biosci. Landmark* 19:528–534. 10.2741/4223 24389200

[B20] Lang Q. Ling C. (2012). MiR-124 suppresses cell proliferation in hepatocellular carcinoma by targeting PIK3CA. *Biochem. Biophys. Res. Commun.* 426 247–252. 10.1016/j.bbrc.2012.08.075 22940133

[B21] Lee Y. S. Dutta A. (2009). MicroRNAs in cancer. *Annu. Rev. Pthol.* 4 199–227. 10.1146/annurev.pathol.4.110807.092222 PMC2769253 18817506

[B22] Li L. Zhao G. Shi Z. Qi L.-L. Zhou L.-Y. Fu Z. (2016). The Ras/Raf/MEK/ERK signaling pathway and its role in the occurrence and development of HCC. *Oncol. Lett.* 12 3045–3050. 10.3892/ol.2016.5110 27899961 PMC5103898

[B23] Li Y. Bai W. Zhang J. (2017). MiR-200c-5p suppresses proliferation and metastasis of human hepatocellular carcinoma (HCC) via suppressing MAD2L1. *Biomed. Pharmacother.* 92 1038–1044. 10.1016/j.biopha.2017.05.092 28609841

[B24] Liang H. Wei P. Hung C. Wu C. Wang W. Huang M. (2013). MicroRNA-200a/b influenced the therapeutic effects of curcumin in hepatocellular carcinoma (HCC) cells. *Tumour Biol.* 34 3209–3218. 10.1007/s13277-013-0891-z 23760980

[B25] Lin Q. Sun M.-Z. Guo C. Shi J. Chen X. Liu S. (2015). CRKL overexpression suppresses *in vitro* proliferation, invasion and migration of murine hepatocarcinoma Hca-P cells. *Biomed. Pharmacother.* 69 11–17. 10.1016/j.biopha.2014.10.025 25661331

[B26] Liu C. Chen T. Chau G. Y. Jan Y. Chen C. Hsu C. N. (2013a). Analysis of protein-protein interactions in cross-talk pathways reveals CRKL protein as a novel prognostic marker in hepatocellular carcinoma. *Mol. Cell Proteomics* 12 1335–1349. 10.1074/mcp.o112.020404 23397142 PMC3650343

[B27] Liu C. Chen T. Chen C. Kao C. Huang C. (2013b). Differential network biology reveals a positive correlation between a novel protein-protein interaction and cancer cells migration. *Conf. Proc. IEEE Eng. Med. Biol. Soc.* 2013 2700–2703. 10.1109/EMBC.2013.6610097 24110284

[B28] Liu S. Guo C. Wang J. Wang B. Qi H. Sun M.-Z. (2016). ANXA11 regulates the tumorigenesis, lymph node metastasis and 5-fluorouracil sensitivity of murine hepatocarcinoma Hca-P cells by targeting c-Jun. *Oncotarget* 7 16297–16310. 10.18632/oncotarget.7484 26908448 PMC4941315

[B29] Llovet J. M. Di, Bisceglie A. M. Bruix J. Kramer B. S. Lencioni R. Zhu A. (2008). Design and endpoints of clinical trials in hepatocellular carcinoma. *J. Natl. Cancer Inst.* 100 698–711. 10.1093/jnci/djn134 18477802

[B30] Long H. Ma Y. Yang H. Xue S. Liu J. Yu F. (2018). Reduced hsa-miR-124-3p levels are associated with the poor survival of patients with hepatocellular carcinoma. *Mol. Biol. Rep.* 45 2615–2623. 10.1007/s11033-018-4431-1 30341691

[B31] Luo L. Chi H. Ling J. (2018). MiR-124-3p suppresses glioma aggressiveness via targeting of Fra-2. *Pathol. Res. Pract.* 214 1825–1834. 10.1016/j.prp.2018.09.017 30243808

[B32] Lv S. Qin J. Yi R. Coreman M. Shi R. Kang H. (2013). CrkL efficiently mediates cell proliferation, migration, and invasion induced by TGF-beta pathway in glioblastoma. *J. Mol. Neurosci.* 51 1046–1051. 10.1007/s12031-013-0096-3 23959425

[B33] Mahmoudvand S. Shokri S. Taherkhani R. Farshadpour F. (2019). Hepatitis C virus core protein modulates several signaling pathways involved in hepatocellular carcinoma. *World J. Gastroenterol.* 25 42–58. 10.3748/wjg.v25.i1.42 30643357 PMC6328967

[B34] Mathias R. A. Simpson R. J. (2009). Towards understanding epithelial-mesenchymal transition: a proteomics perspective. *Biochim. Biophy. Acta* 1794 1325–1331. 10.1016/j.bbapap.2009.05.001 19439204

[B35] McCubrey J. A. Steelman L. S. Chappell W. H. Abrams S. L. Wong E. W. Chang F. (2007). Roles of the Raf/MEK/ERK pathway in cell growth, malignant transformation and drug resistance. *Biochim. Biophy. Acta* 1773 1263–1284. 10.1016/j.bbamcr.2006.10.001 17126425 PMC2696318

[B36] Mortazavi F. Dubinett S. Rettig M. (2011). c-Crk proto-oncogene contributes to transcriptional repression of p120-catenin in non-small cell lung cancer cells. *Clin. Exp. Metast*. 28 391–404. 10.1007/s10585-011-9378-8 21336985 PMC3081060

[B37] Pan J. Hu H. Zhou Z. Sun L. Peng L. Yu L. (2010). Tumor-suppressive mir-663 gene induces mitotic catastrophe growth arrest in human gastric cancer cells. *Oncol. Rep.* 24 105–112. 20514450 10.3892/or_00000834

[B38] Park T. Koptyra M. Curran T. (2016). Fibroblast growth requires CT10 regulator of kinase (Crk) and Crk-like (CrkL). *J. Biol. Chem.* 291 26273–26290. 10.1074/jbc.m116.764613 27807028 PMC5159491

[B39] Qin W. Pan Y. Zheng X. Li D. Bu J. Xu C. (2014). MicroRNA-124 regulates TGF-alpha-induced epithelial-mesenchymal transition in human prostate cancer cells. *Int. J. Oncol.* 45 1225–1231. 10.3892/ijo.2014.2506 24969691

[B40] Rashmi R. Kumar S. Karunagaran D. (2005). Human colon cancer cells lacking Bax resist curcumin-induced apoptosis and Bax requirement is dispensable with ectopic expression of Smac or downregulation of Bcl-XL. *Carcinogenesis* 26 713–723. 10.1093/carcin/bgi025 15661804

[B41] Ren Y. Shang J. Li J. Liu W. Zhang Z. Yuan J. (2017). The long noncoding RNA PCAT-1 links the microRNA miR-215 to oncogene CRKL-mediated signaling in hepatocellular carcinoma. *J. Biol. Chem.* 292 17939–17949. 10.1074/jbc.m116.773978 28887306 PMC5663891

[B42] Shi J. Meng L. Sun M.-Z. Guo C. Sun X. Lin Q. (2015). CRKL knockdown promotes in vitro proliferation, migration and invasion, *in vivo* tumor malignancy and lymph node metastasis of murine hepatocarcinoma Hca-P cells. *Biomed. Pharmacother.* 71 84–90. 10.1016/j.biopha.2015.02.022 25960220

[B43] Sriram G. Birge R. B. (2010). Emerging roles for crk in human cancer. *Genes Cancer* 1 1132–1139. 10.1177/1947601910397188 21779437 PMC3092275

[B44] Tsuda M. Makino Y. Iwahara T. Nishihara H. Sawa H. Nagashima K. (2004). Crk associates with ERM proteins and promotes cell motility toward hyaluronic acid. *J. Biol. Chem.* 279 46843–46850. 10.1074/jbc.m401476200 15326184

[B45] Voulgari A. Pintzas A. (2009). Epithelial-mesenchymal transition in cancer metastasis: mechanisms, markers and strategies to overcome drug resistance in the clinic. *Biochim. Biophys. Acta* 1796 75–90. 10.1016/j.bbcan.2009.03.002 19306912

[B46] Wang Y. Chen L. Wu Z. Wang M. Jin F. Wang N. (2016a). miR-124-3p functions as a tumor suppressor in breast cancer by targeting CBL. *BMC Cancer* 16:826. 10.1186/s12885-016-2862-4 27842510 PMC5109743

[B47] Wang Y. Dong X. Hu B. Wang X. Wang Q. Wang W. (2016b). The effects of micro-429 on inhibition of cervical cancer cells through targeting ZEB1 and CRKL. *Biomed. Pharmacother.* 80 311–321. 10.1016/j.biopha.2016.03.035 27133071

[B48] Wu Q. Xu L. Wang C. Fan W. Yan H. Li Q. (2018). MicroRNA-124-3p represses cell growth and cell motility by targeting EphA2 in glioma. *Biochem. Biophys. Res. Commun.* 503 2436–2442. 10.1016/j.bbrc.2018.06.173 29969628

[B49] Xia J. Wu Z. Yu C. He W. Zheng H. He Y. (2012). miR-124 inhibits cell proliferation in gastric cancer through down-regulation of SPHK1. *J. Pathol.* 227 470–480. 10.1002/path.4030 22450659

[B50] Yu J. Zhang L. (2005). The transcriptional targets of p53 in apoptosis control. *Biochem. Biophys. Res. Commun.* 331 851–858. 10.1016/j.bbrc.2005.03.189 15865941

[B51] Yuan Q. Sun T. Ye F. Kong W. Jin H. (2017). MicroRNA-124-3p affects proliferation, migration and apoptosis of bladder cancer cells through targeting AURKA. *Cancer Biomark*. 19 93–101. 10.3233/cbm-160427 28269755 PMC13020699

[B52] Zhang X. Cai D. Meng L. Wang B. (2016). MicroRNA-124 inhibits proliferation, invasion, migration and epithelial-mesenchymal transition of cervical carcinoma cells by targeting astrocyte-elevated gene-1. *Oncol. Rep.* 36 2321–2328. 10.3892/or.2016.5025 27571703

[B53] Zhang Y. Wang Q. Li H. Ye T. Gao F. Liu Y. (2016). miR-124 radiosensitizes human esophageal cancer cell TE-1 by targeting CDK4. *Genet. Mol. Res.* 15:15027893. 10.4238/gmr.15027893 27323123

[B54] Zhao T. Miao Z. Wang Z. Xu Y. Wu J. Liu X. (2013). Overexpression of CRKL correlates with malignant cell proliferation in breast cancer. *Tumour Biol.* 34 2891–2897. 10.1007/s13277-013-0851-7 23686806

[B55] Zheng F. Liao Y. Cai M. Liu Y. Liu T. Chen S. (2012). The putative tumour suppressor microRNA-124 modulates hepatocellular carcinoma cell aggressiveness by repressing ROCK2 and EZH2. *Gut* 61 278–289. 10.1136/gut.2011.239145 21672940

[B56] Zhou X. Zhang C. Lu S. Chen G. Li L. Liu L. (2016). miR-625 suppresses tumour migration and invasion by targeting IGF2BP1 in hepatocellular carcinoma. *Oncogene* 35 5078–5077. 10.1038/onc.2016.61 27499093

