# Supplementary material for: miR-124-3p Suppresses the Invasiveness and Metastasis of Hepatocarcinoma Cells via Targeting CRKL
Source: Front Mol Biosci. 2020 Sep 15;7:223. doi: 10.3389/fmolb.2020.00223 (PMC7522612; doi:10.3389/fmolb.2020.00223)
Supplement: Supplementary file 1 [file Data_Sheet_1.pdf]

Supplementary Material

1 Supplementary Data

1.1 Supplementary Figures (supplementary not used in article)

1.1.1 Supplementary Figures 1 original blots

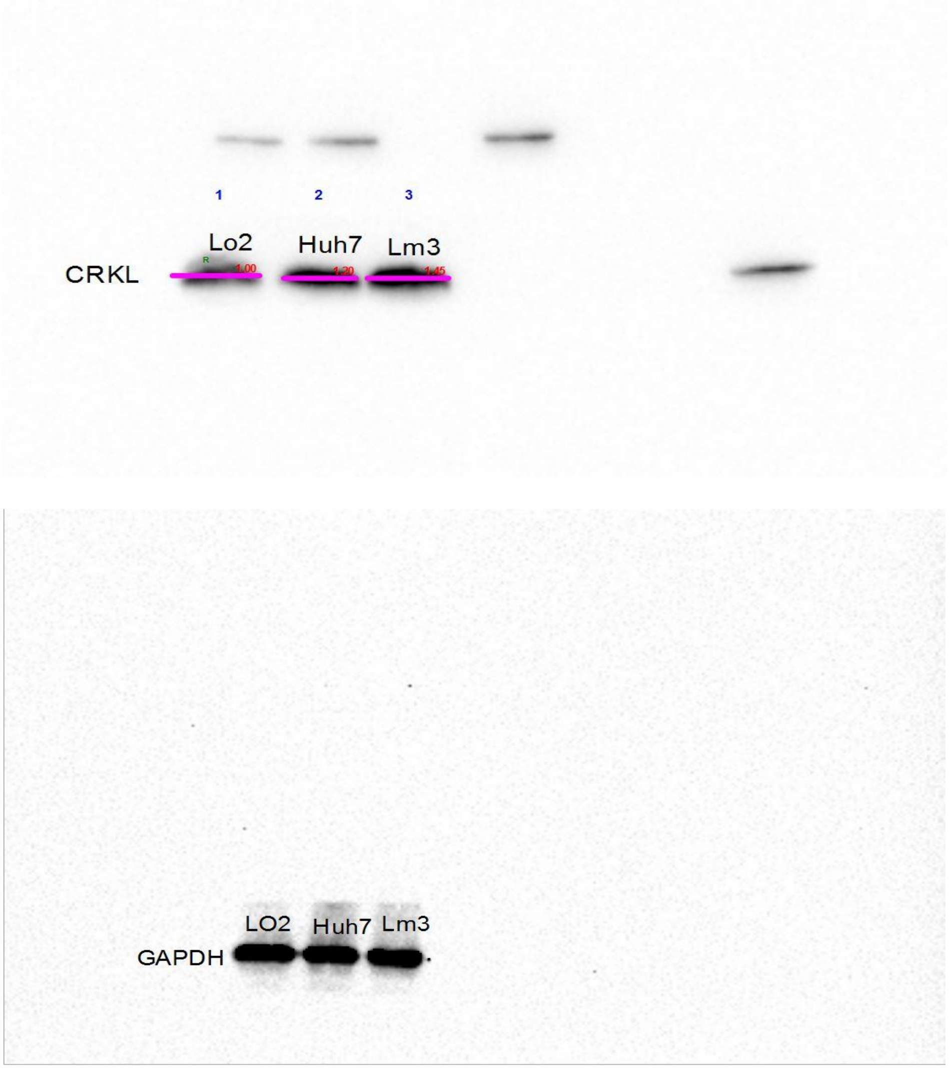

Figure 1 CRKL LO2, Huh7, LM3 Bands including Gapdh

1.2 Supplementary figure 2 (original blots of CRKL transfected with miR-124-3p mimics in HCCLM3)

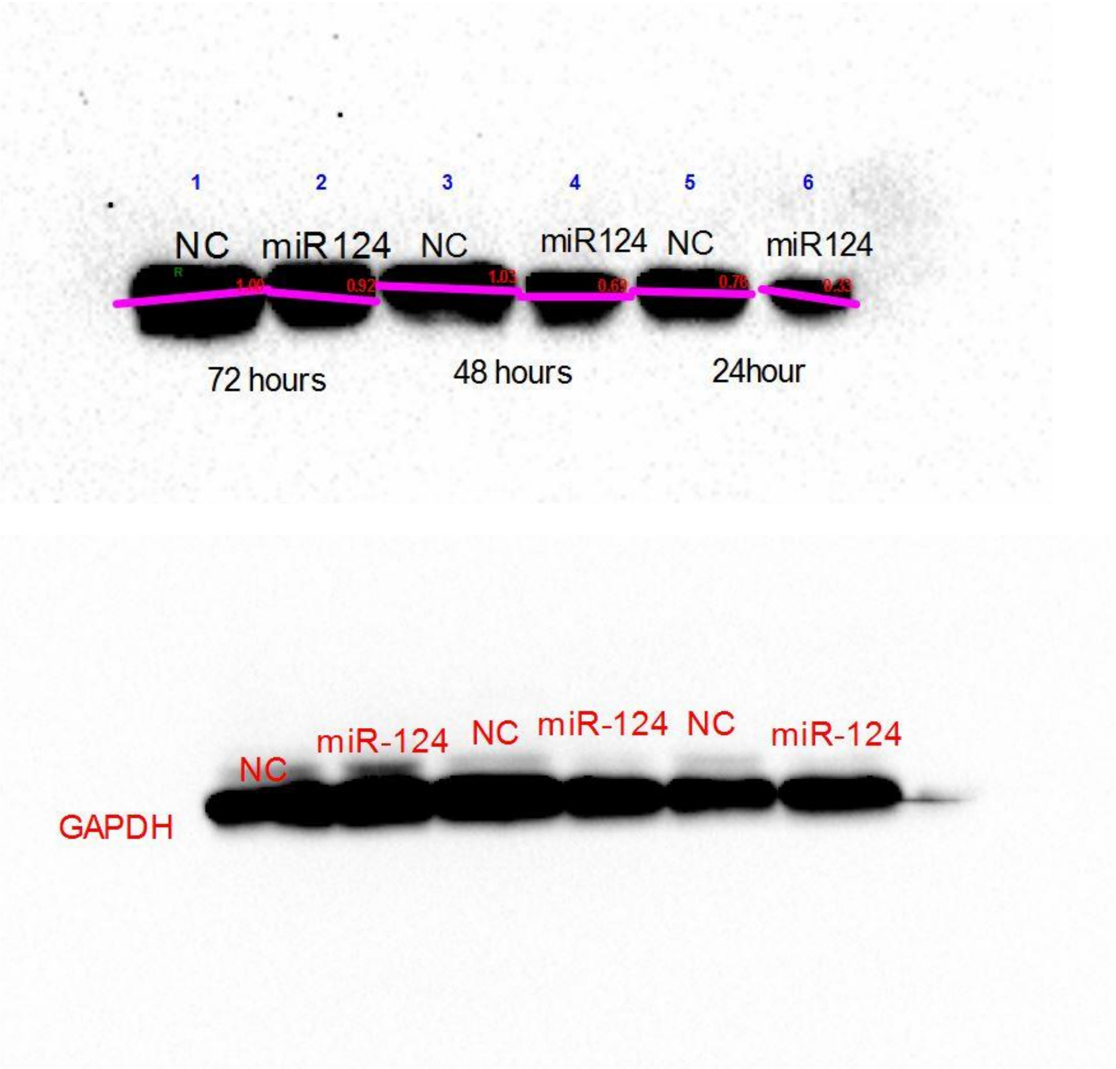

Figure 2 expression of CRKL in transfected HCCLM3 at 24, 48, and 72 hours of post-transfection with miR-124-3p mimics.

1.3 Supplementary figure 3 (original blots of CRKL transfected with miR-124-3p mimics in Huh7)

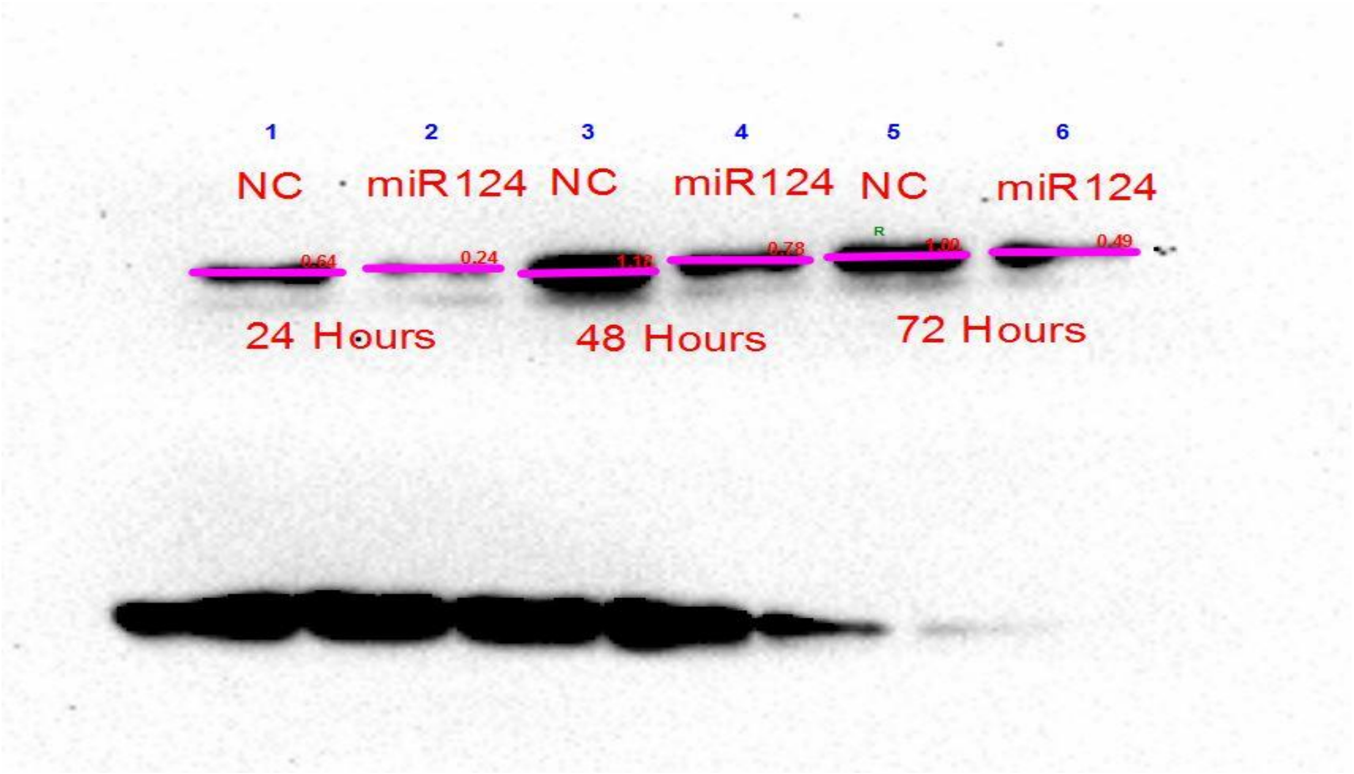
1 2 3 4 5 6
NC miR124 NC miR124 NC miR124
R
0.64
0.24
0.78
0.49
24 Hours
48 Hours
72 Hours

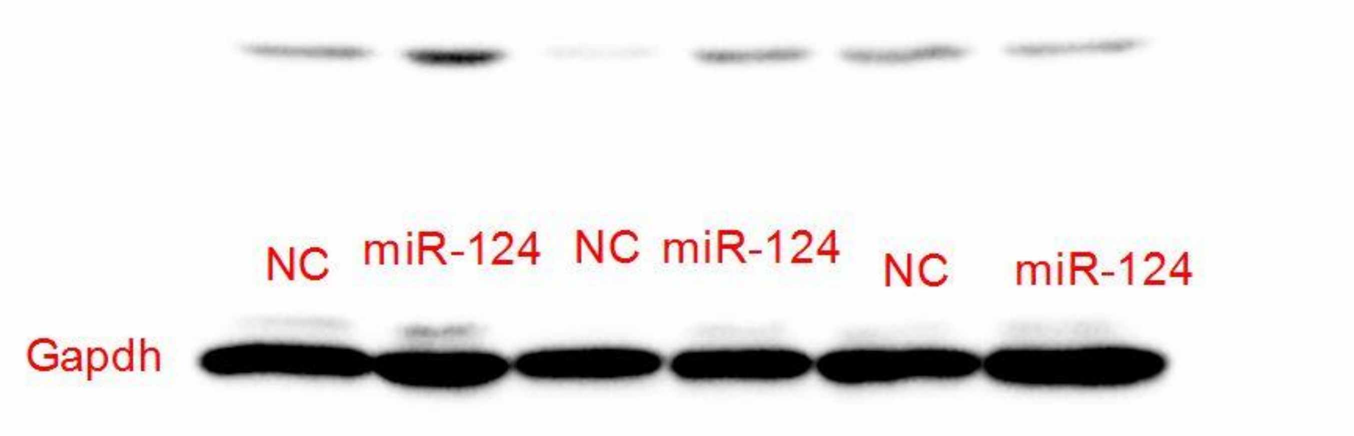
NC miR-124 NC miR-124 NC miR-124
Gapdh

Figure 3 expression of CRKL in transfected Huh7 at 24, 48, and 72 hours of post-transfection with miR-124-3p mimics.

1.4 Supplementary Figure 4 (original blots of signaling proteins, transfected with miR-124-3p mimics in HCCLM3)

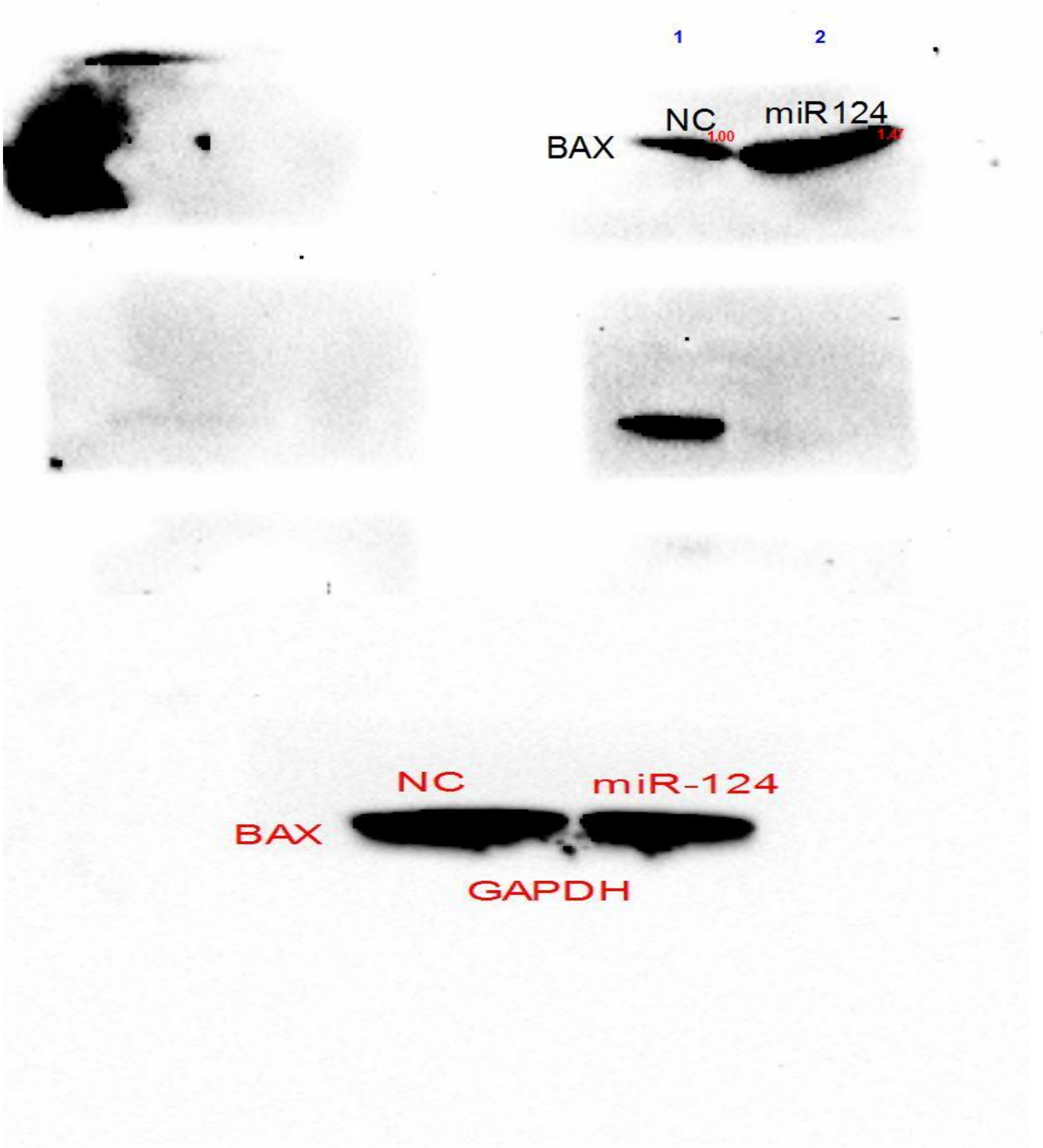

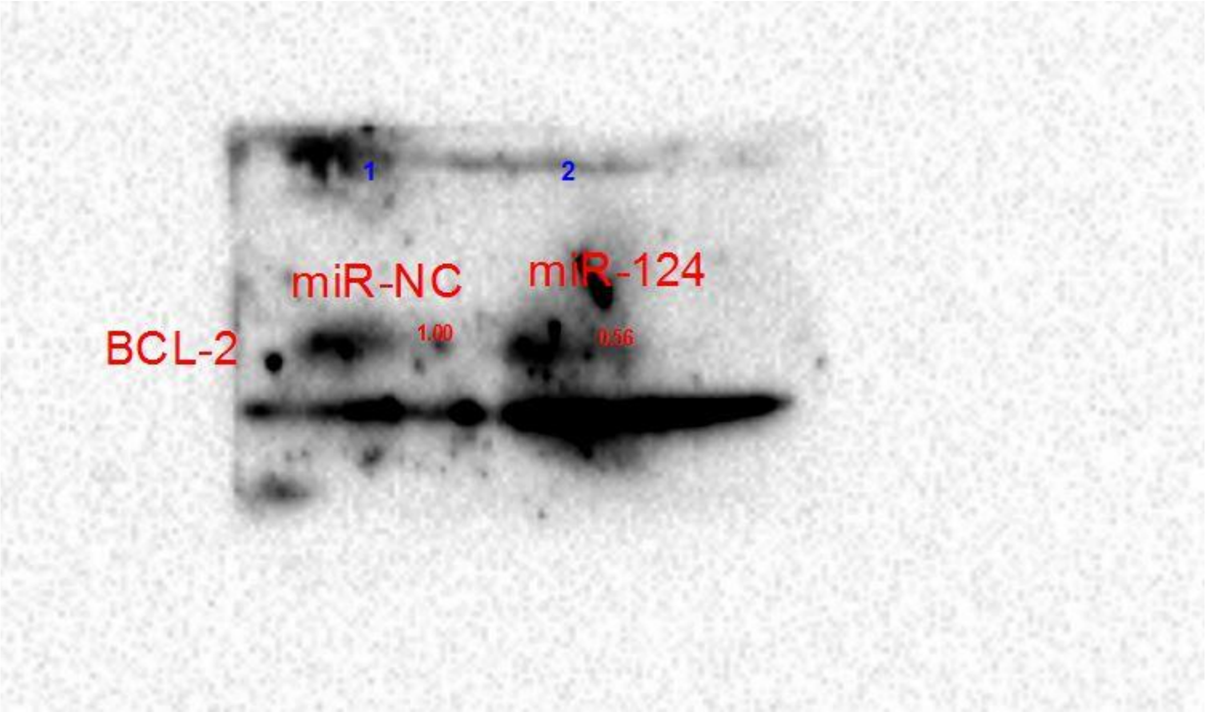
1
2
miR-NC
miR-124
BCL-2
1.00
0.56

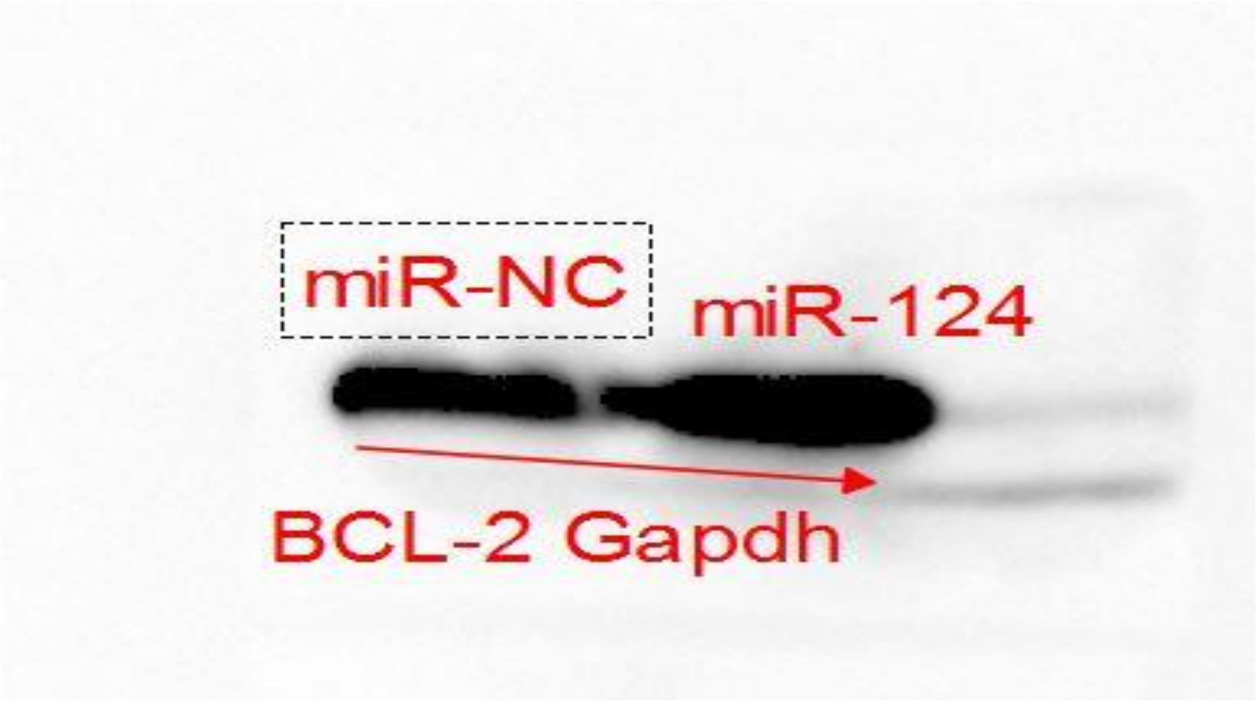
miR-NC
miR-124
BCL-2 Gapdh

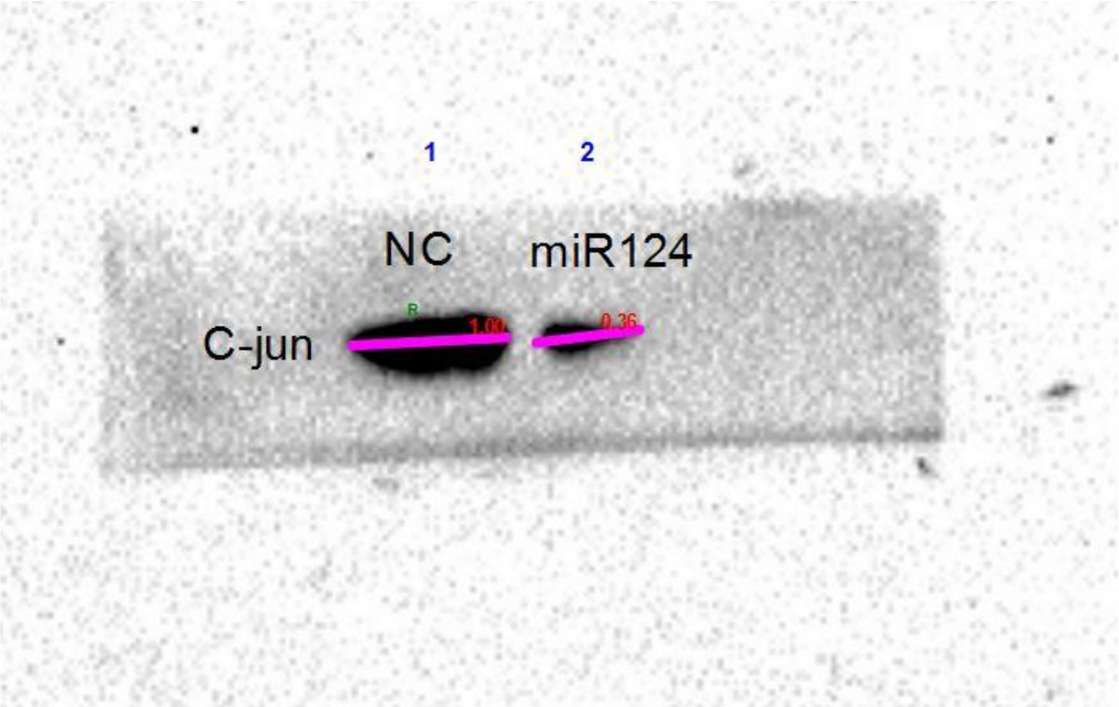
1
2
NC
miR124
C-jun
1.00
0.36

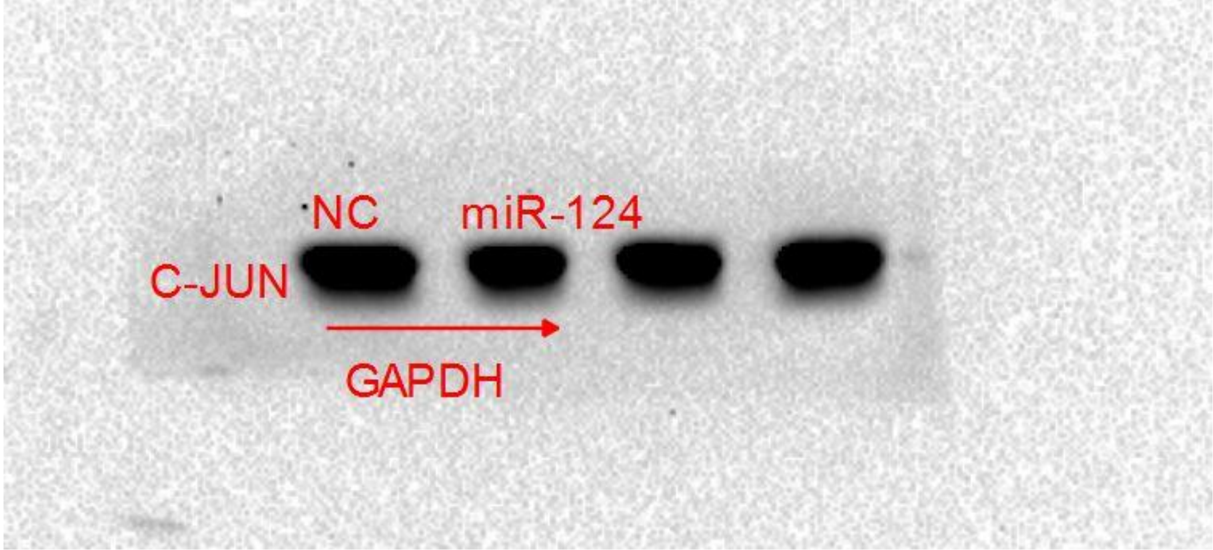
NC
miR-124
C-JUN
GAPDH

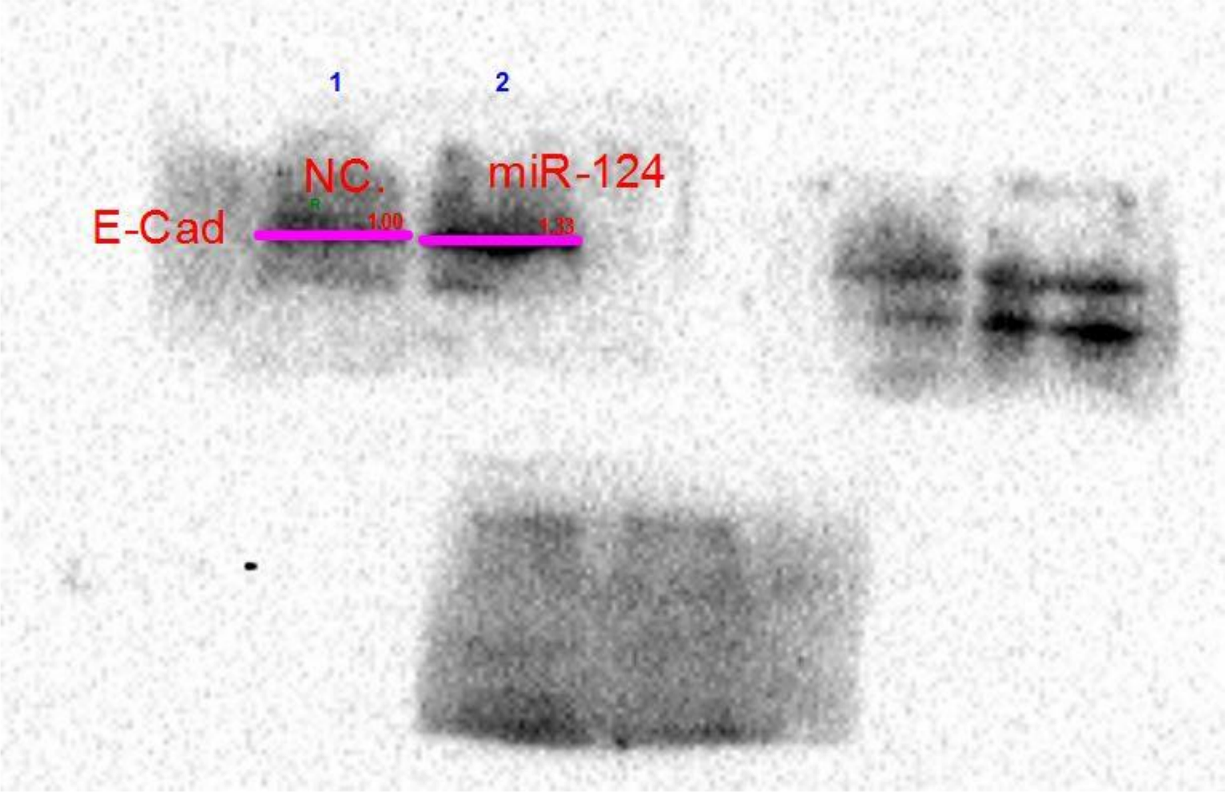
1
2
NC.
miR-124
E-Cad
1.00
1.33

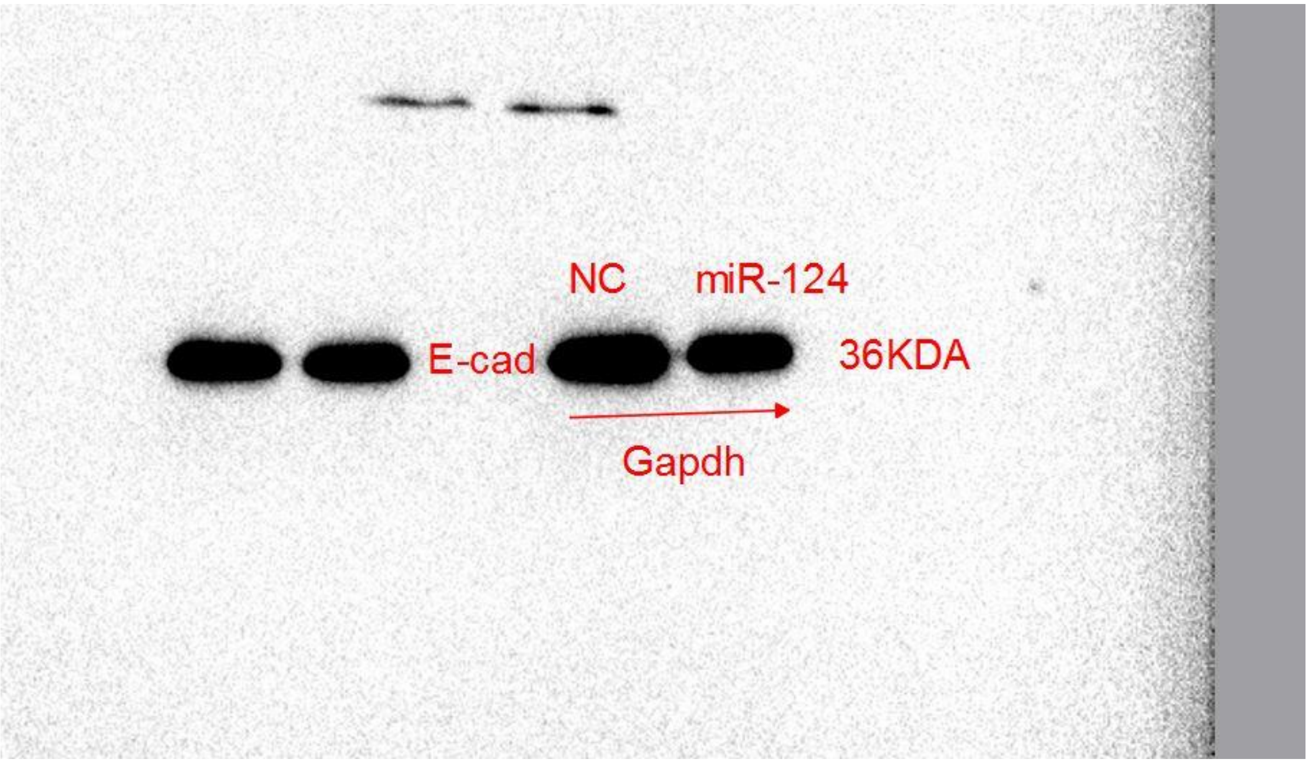
NC
miR-124
E-cad
36KDA
Gapdh

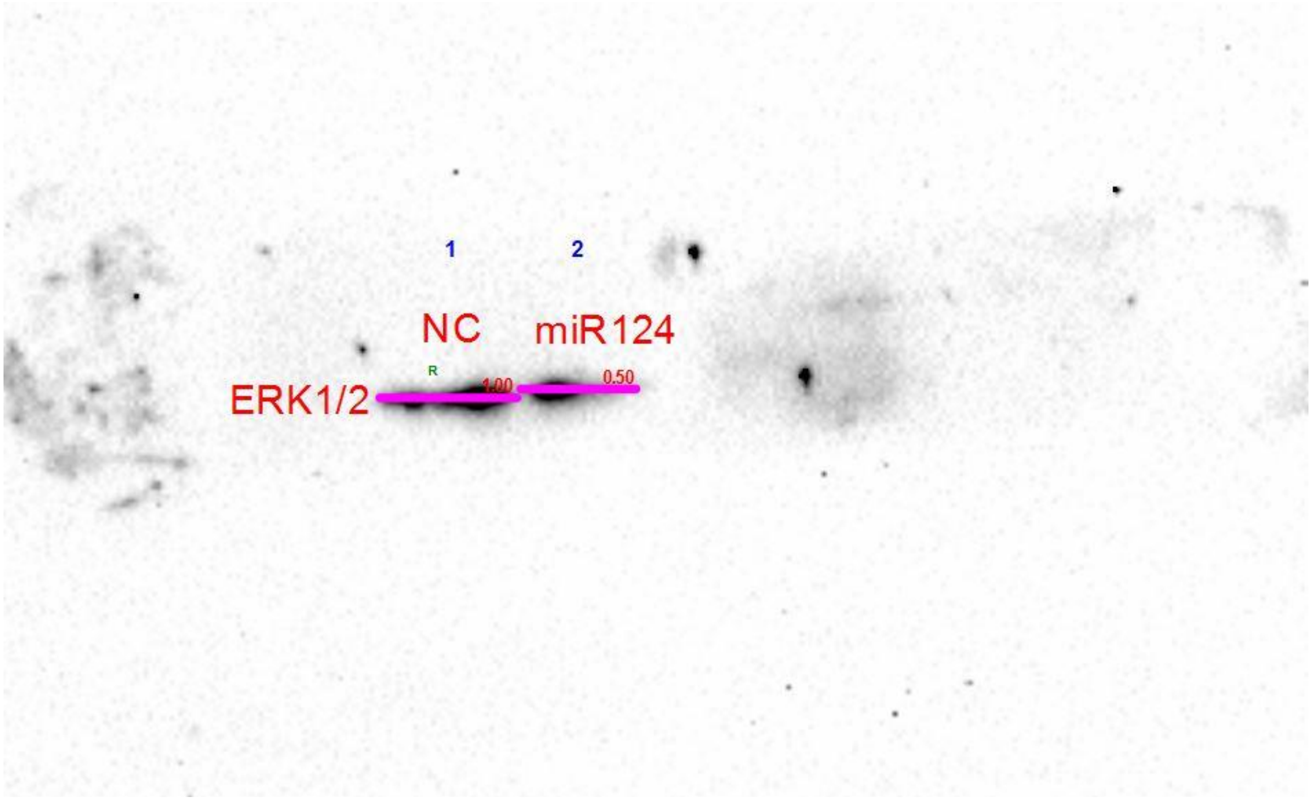
1
2
NC
miR124
ERK1/2
R
1.00
0.50

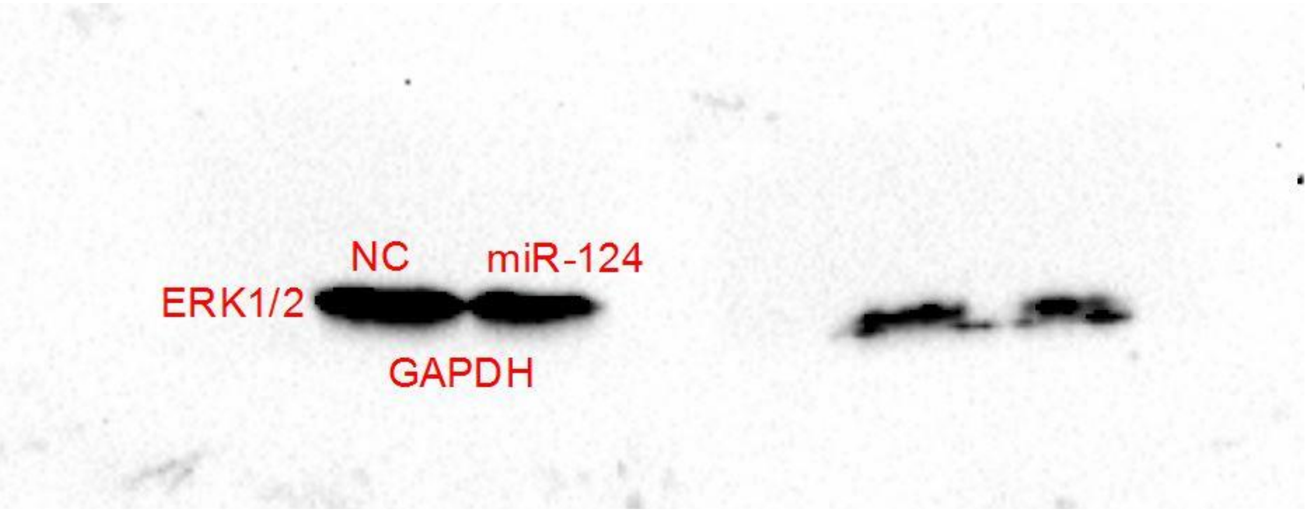
NC
miR-124
ERK1/2
GAPDH

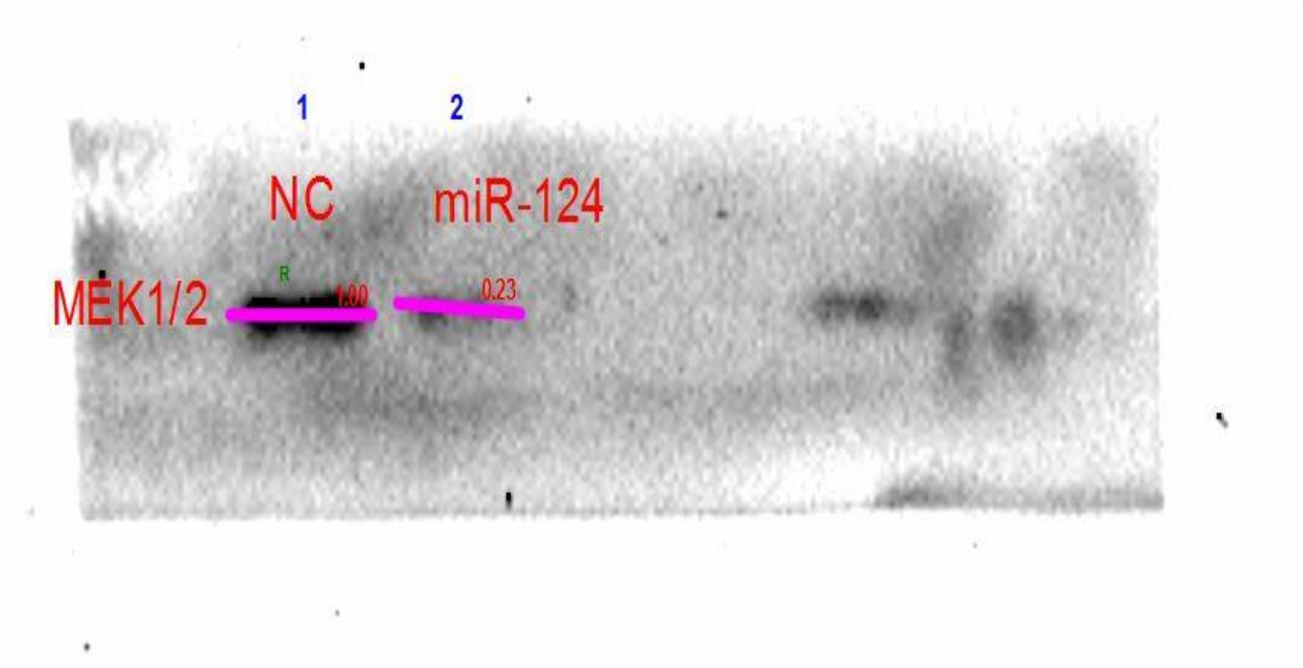
1
2
NC
miR-124
MEK1/2
1.00
0.23

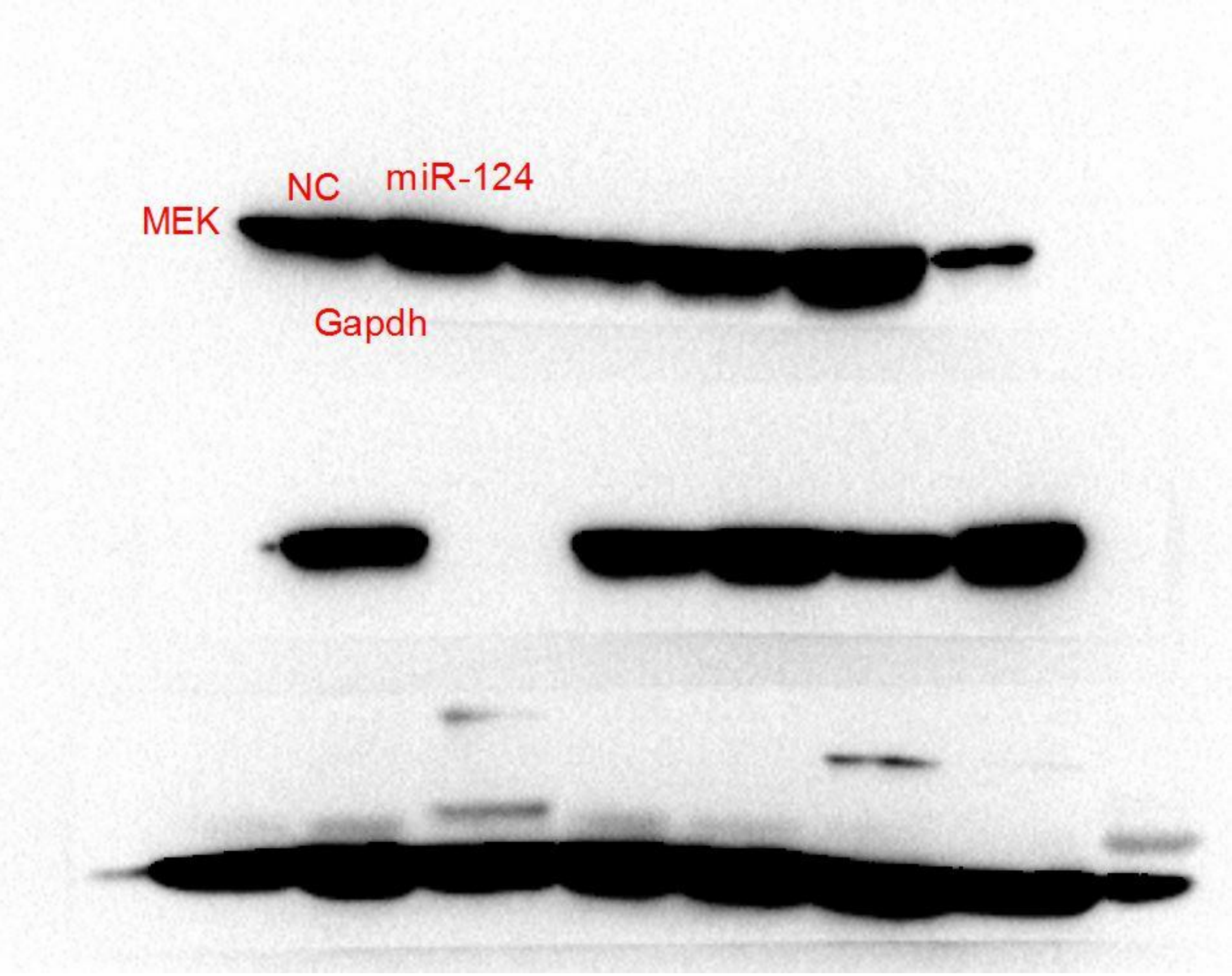
NC
miR-124
MEK
Gapdh

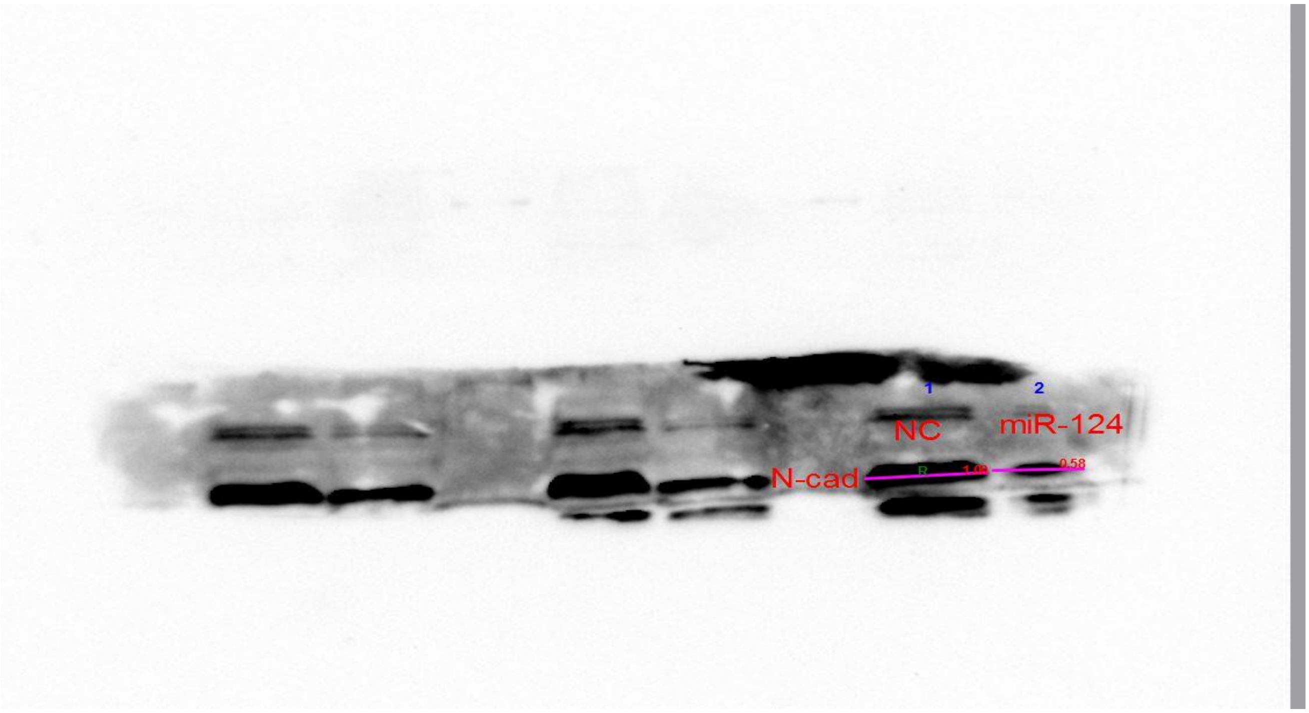
1
2
NC
miR-124
N-cad
R
0.58

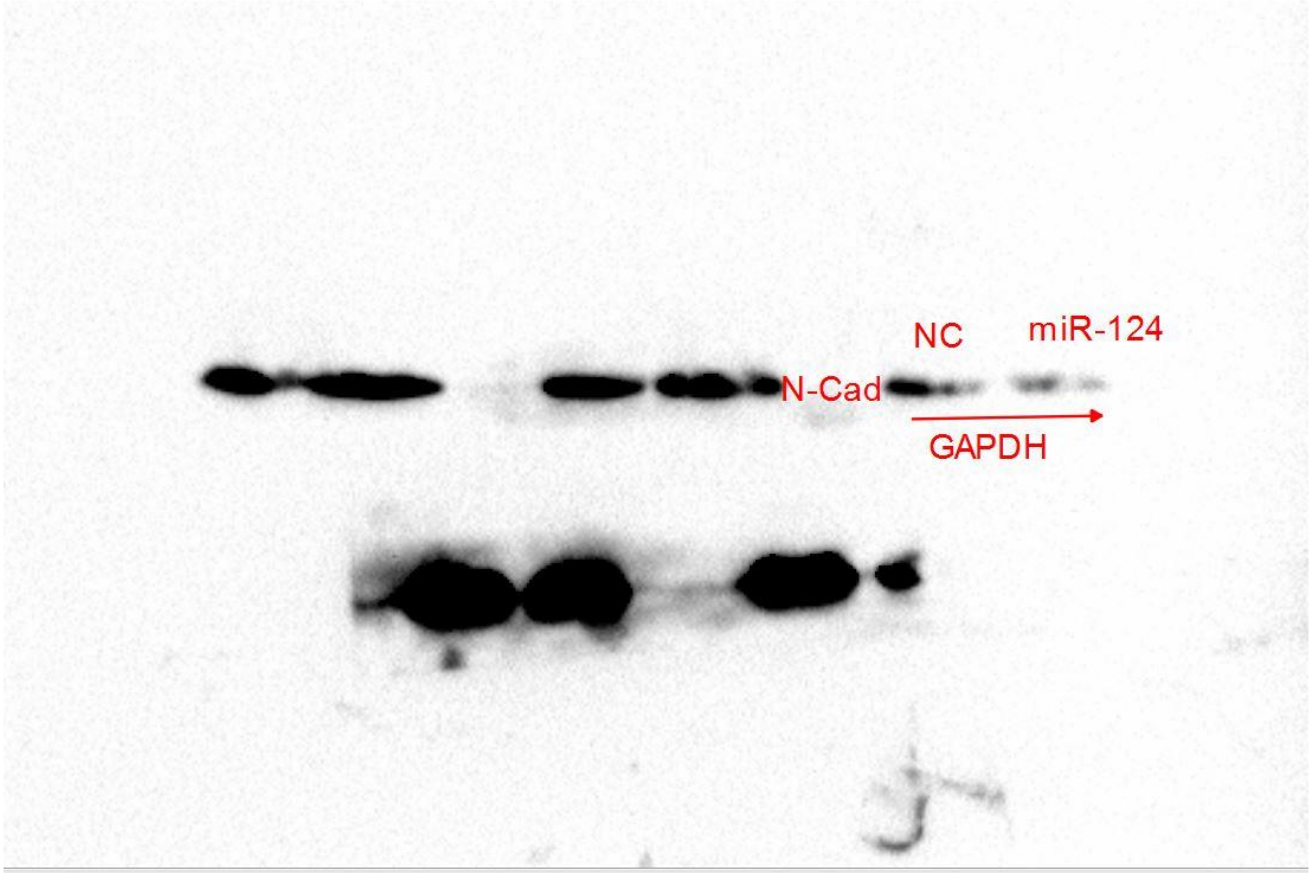
NC
miR-124
N-Cad
GAPDH

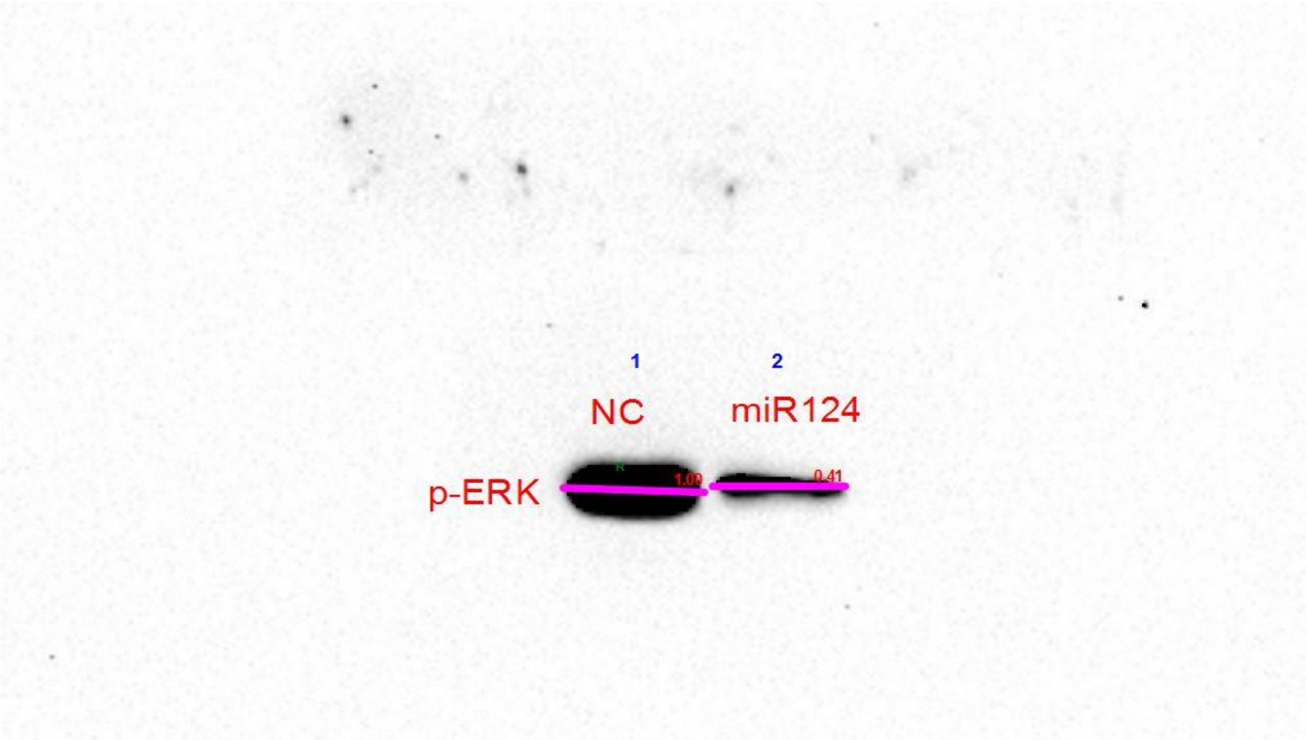
1
2
NC
miR124
p-ERK
1.00
0.41

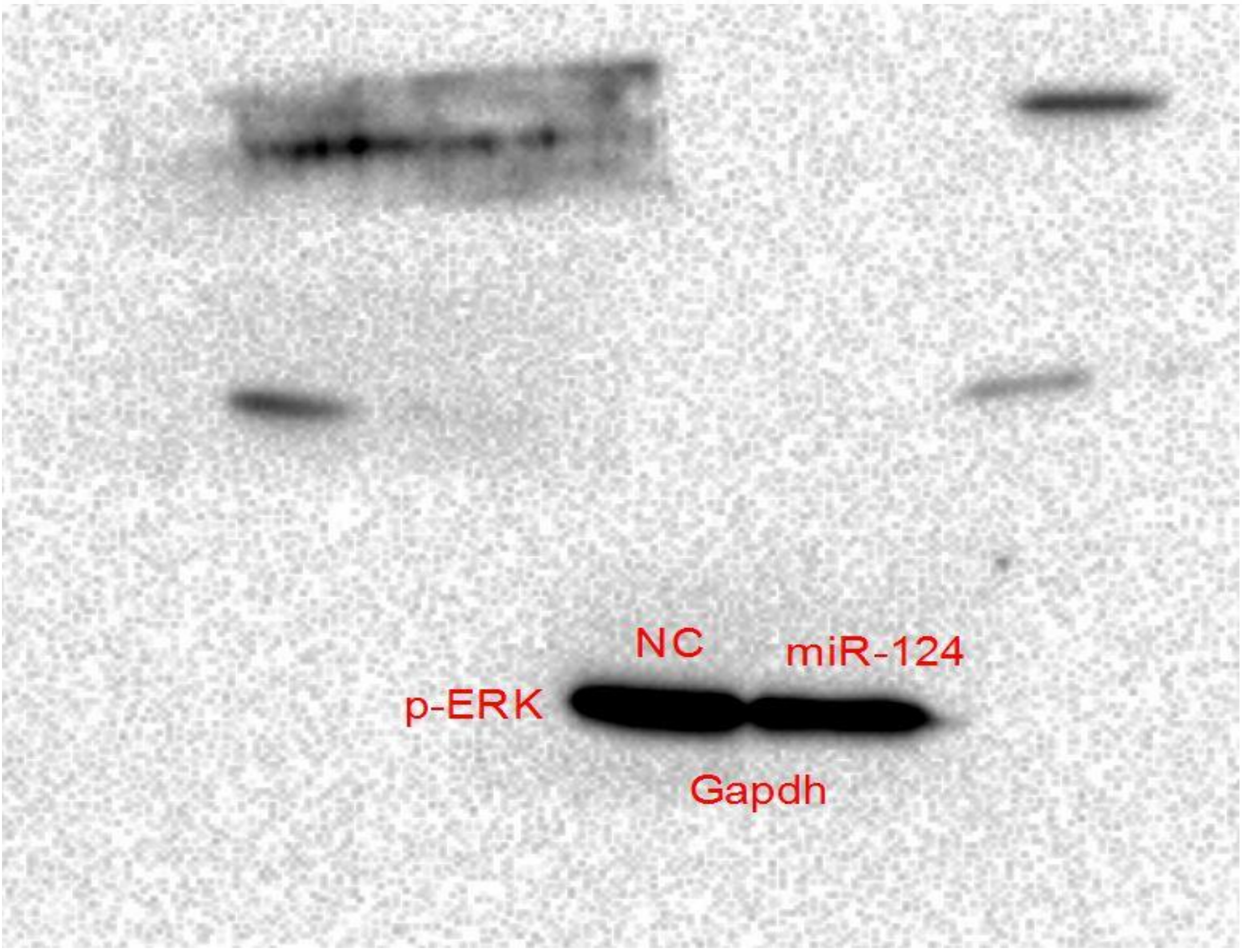
NC
miR-124
p-ERK
Gapdh

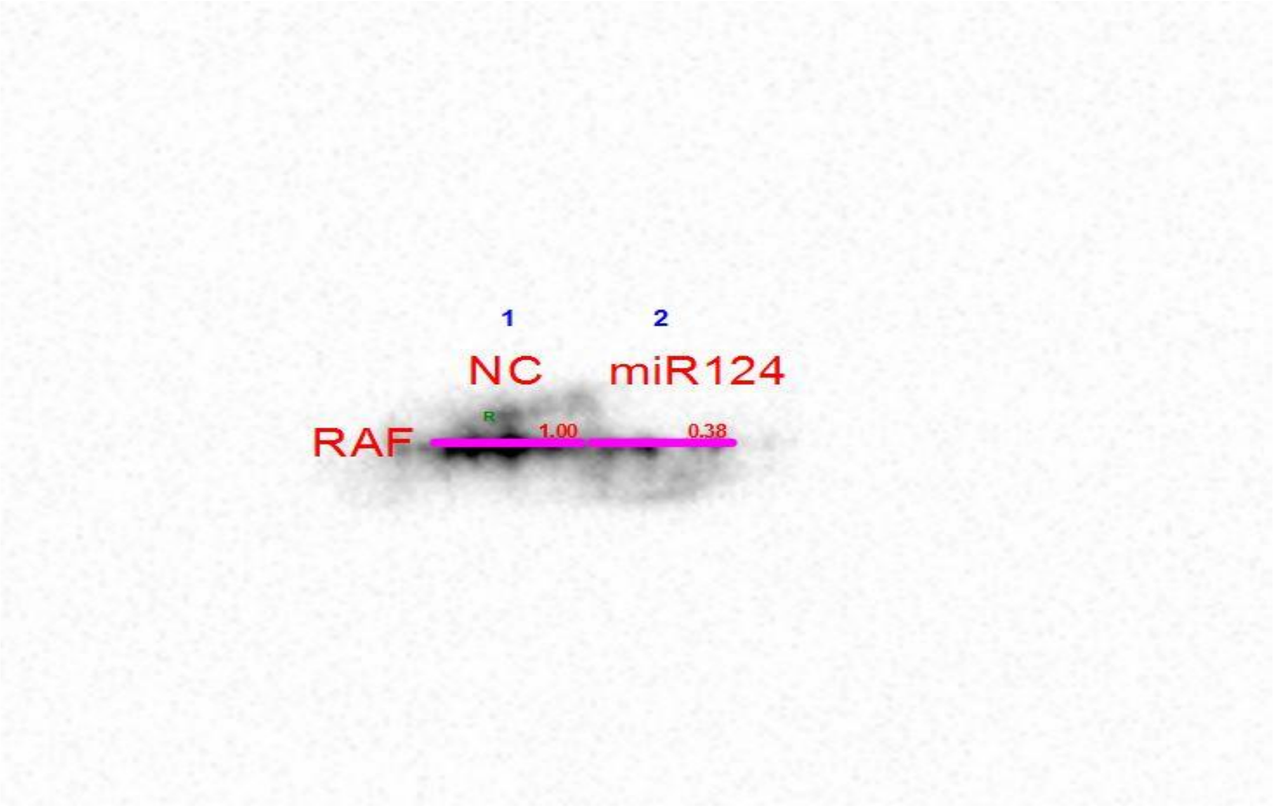
1
2
NC
miR124
RAF
1.00
0.38

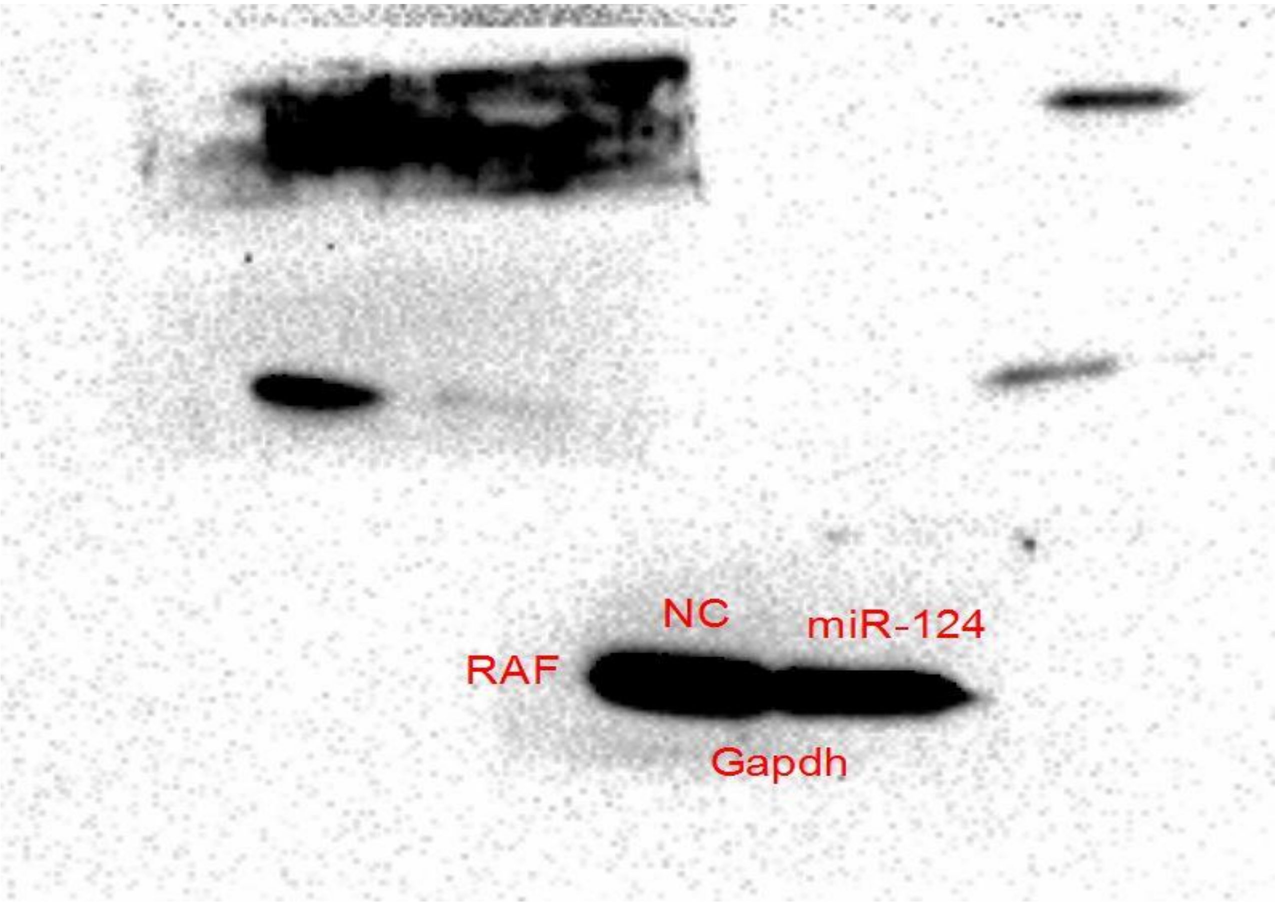
NC
miR-124
RAF
Gapdh

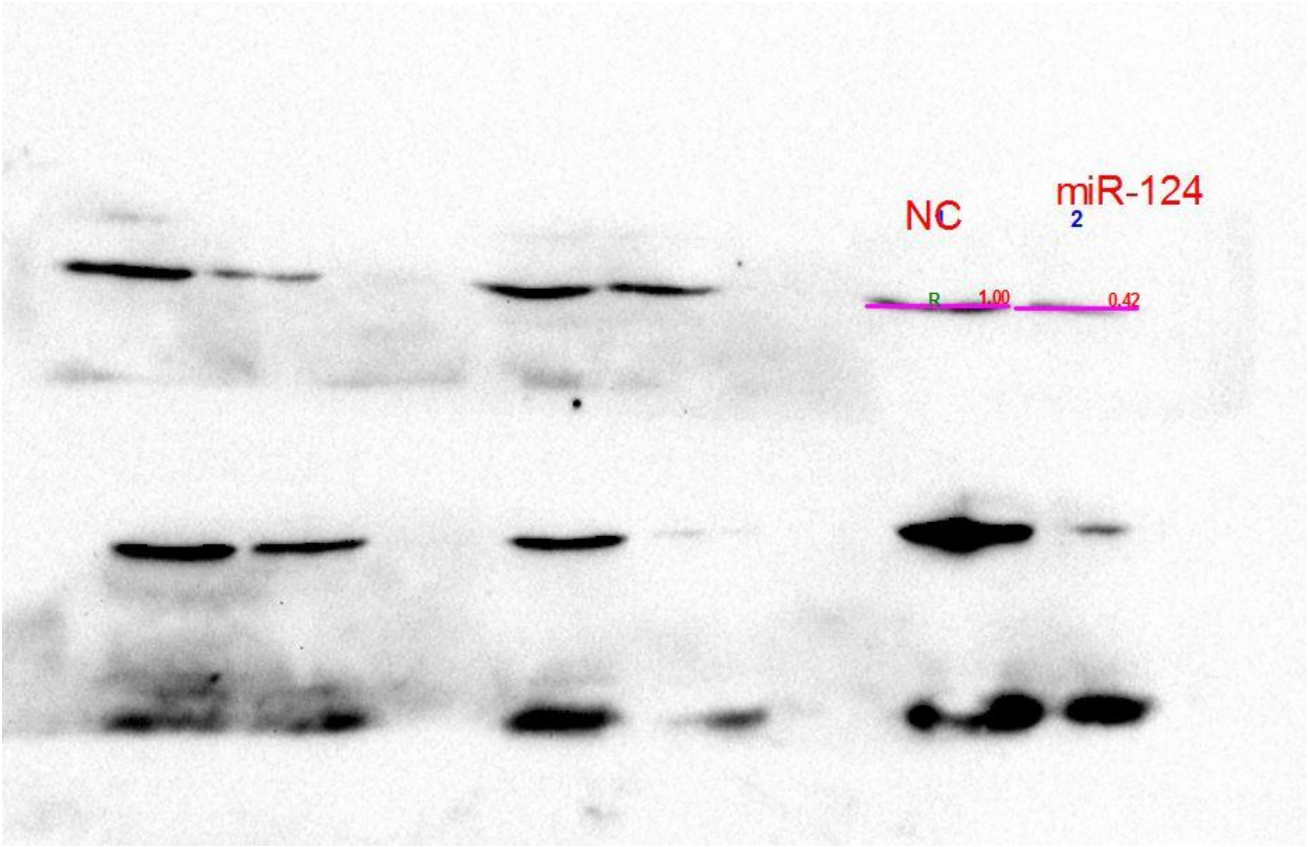
NC
miR-124
2
1.00
0.42

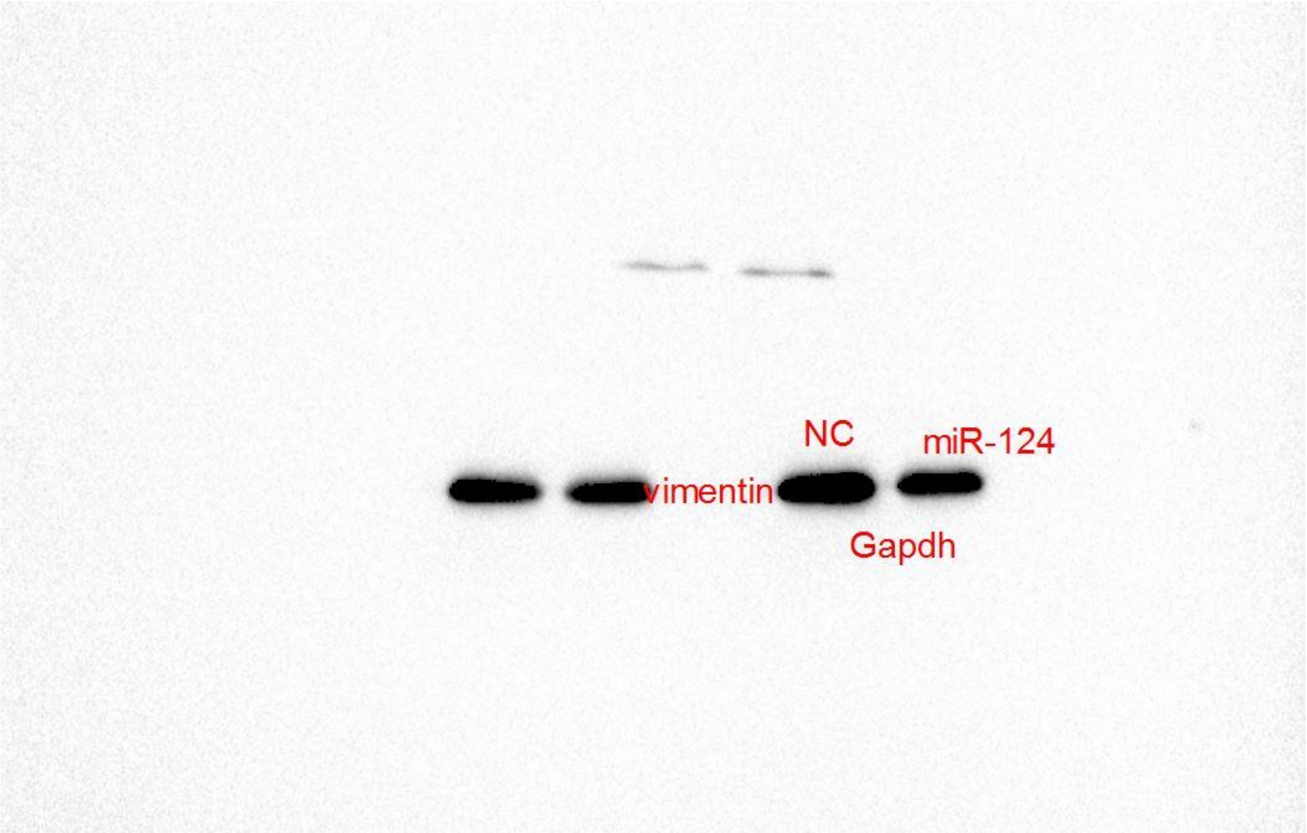
NC
miR-124
vimentin
Gapdh

Figure 4 original Blots of Bax, BCL-2, C-JUN, E-cad, ERK1/2, MEK, N-cad, p-ERK1/2, Raf and vimentin transfected with miR-NC and miR-124-3p mimics in HCCLM3**.**

1.5 Supplementary figure 5 (original blots of signaling proteins, transfected with miR-124-3p mimics in Huh7)

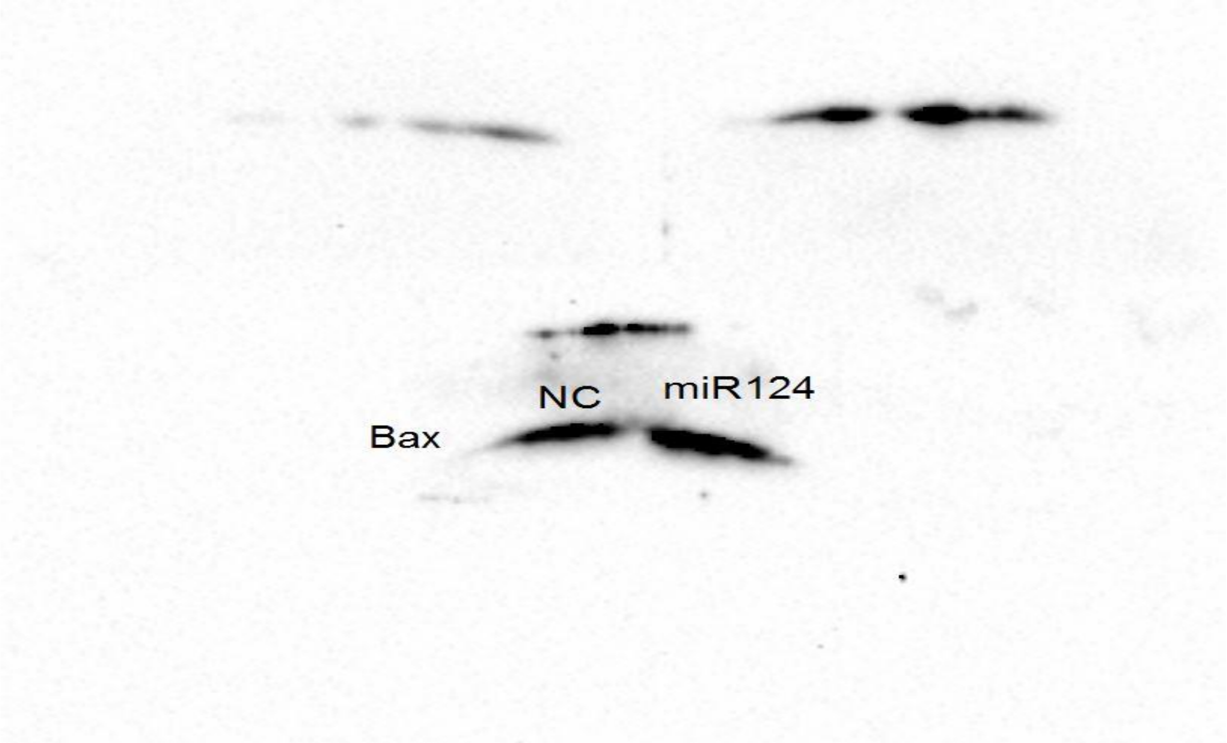

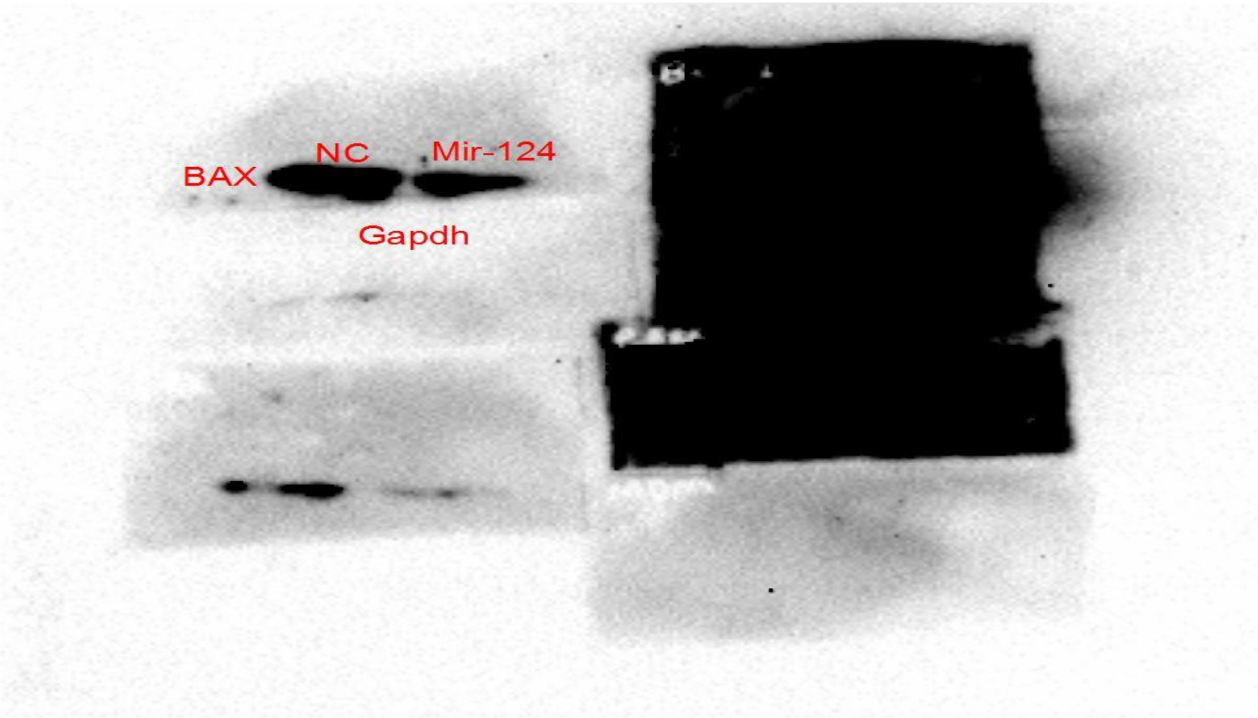

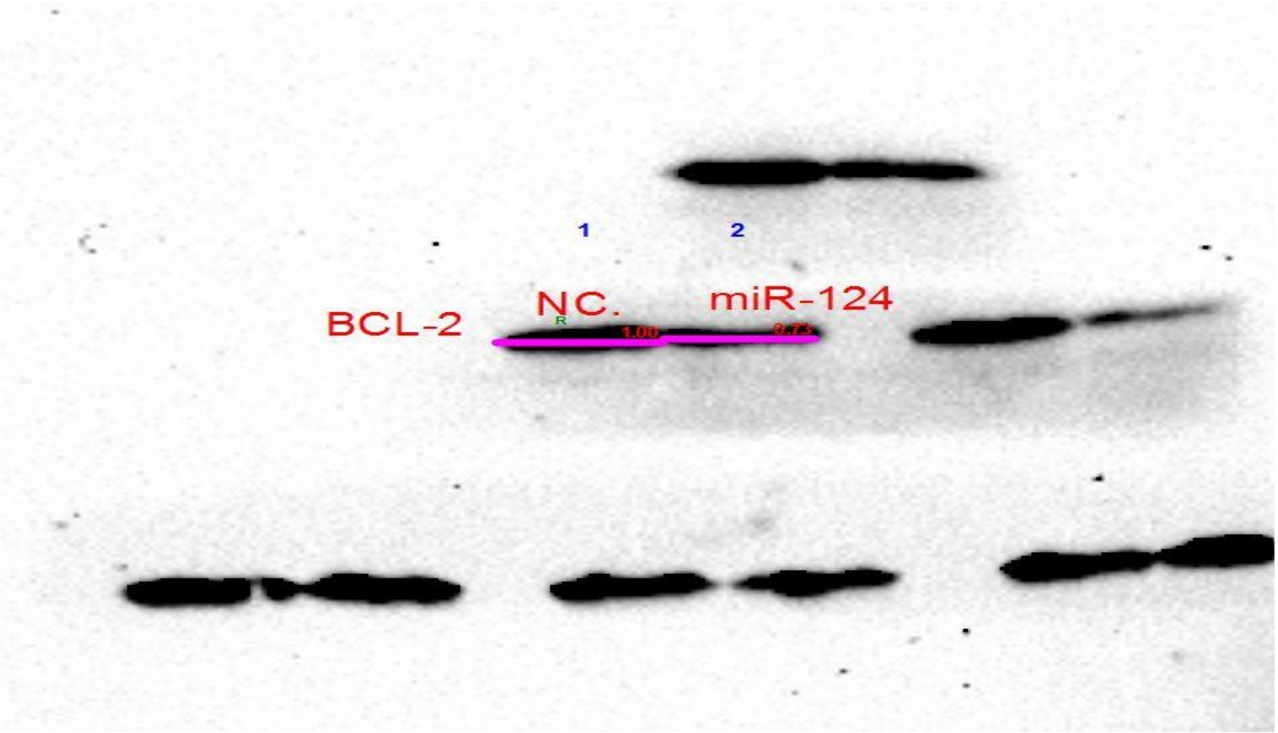
1
2
BCL-2
NC.
miR-124
1.00
0.73

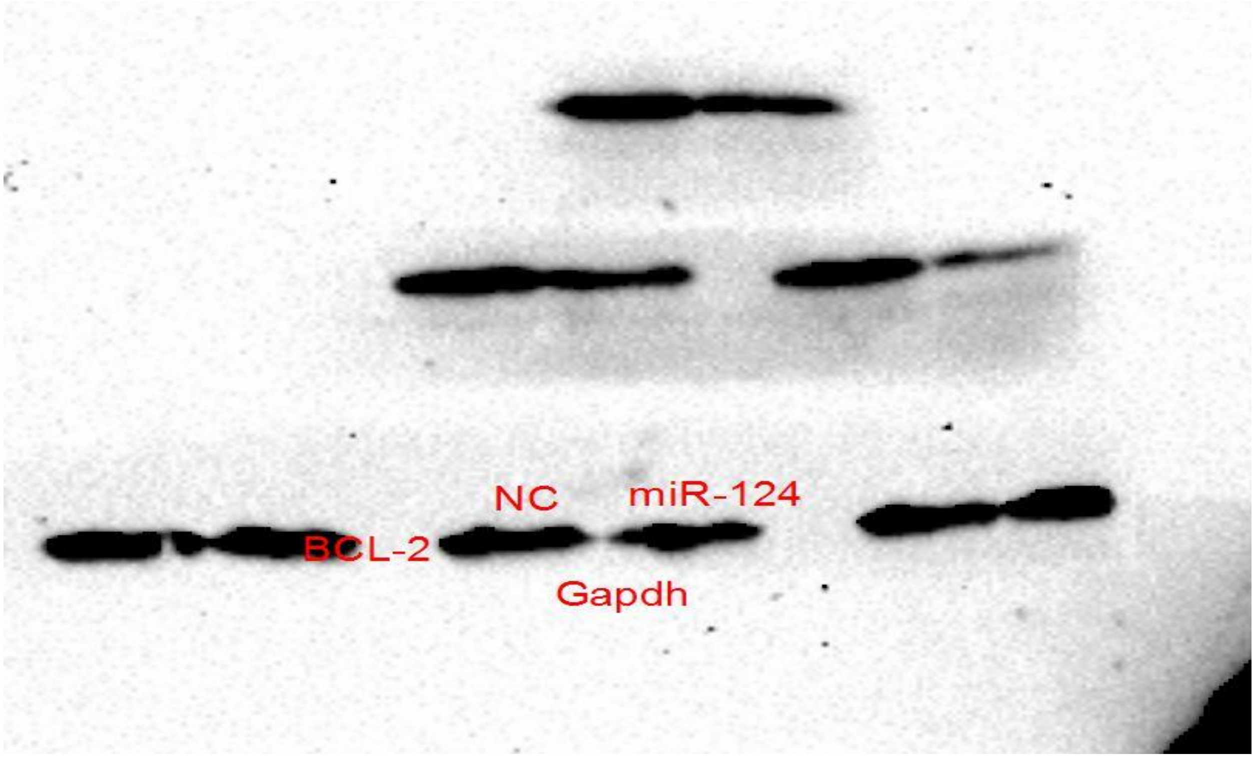
NC
miR-124
BCL-2
Gapdh

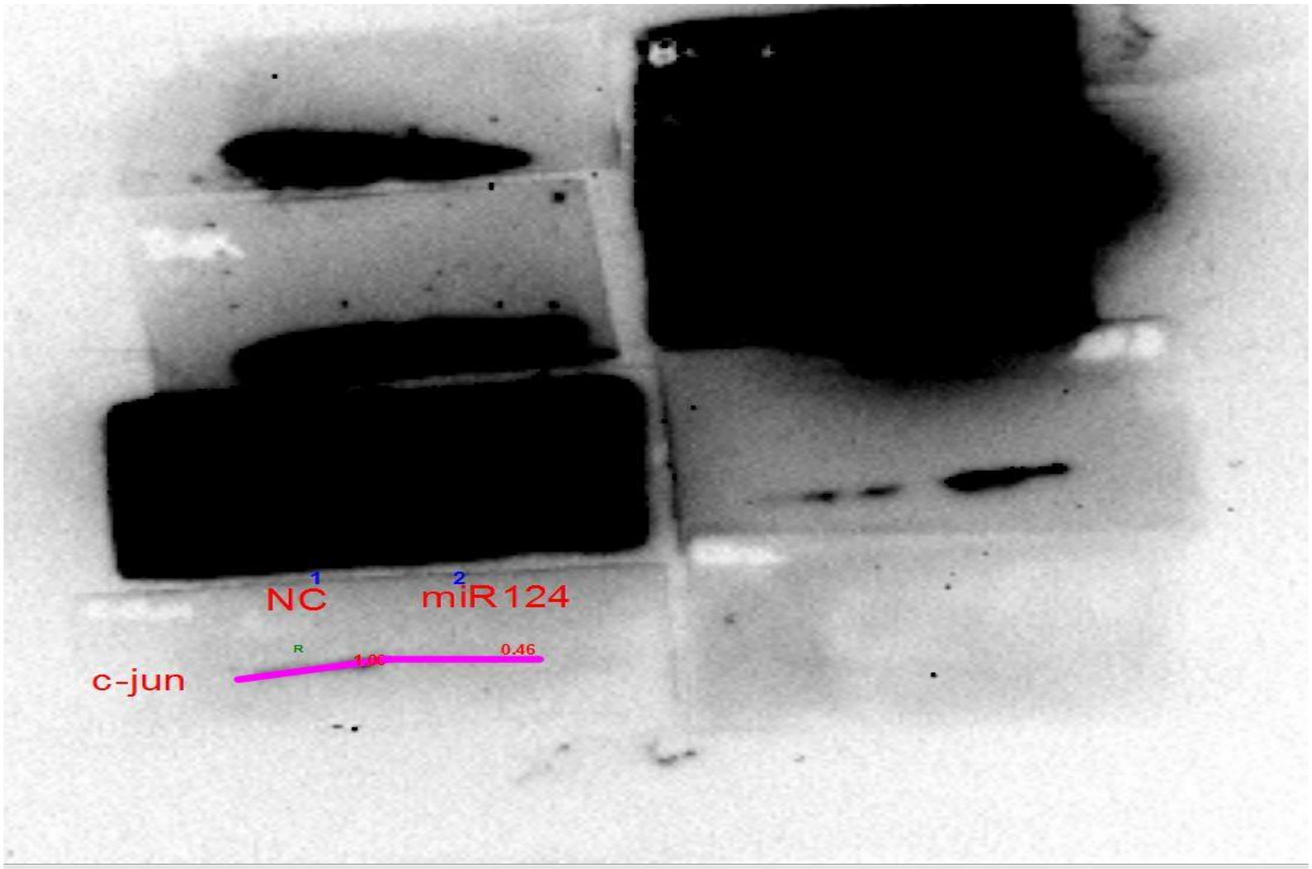
1
2
NC
miR124
R
0.46
c-jun

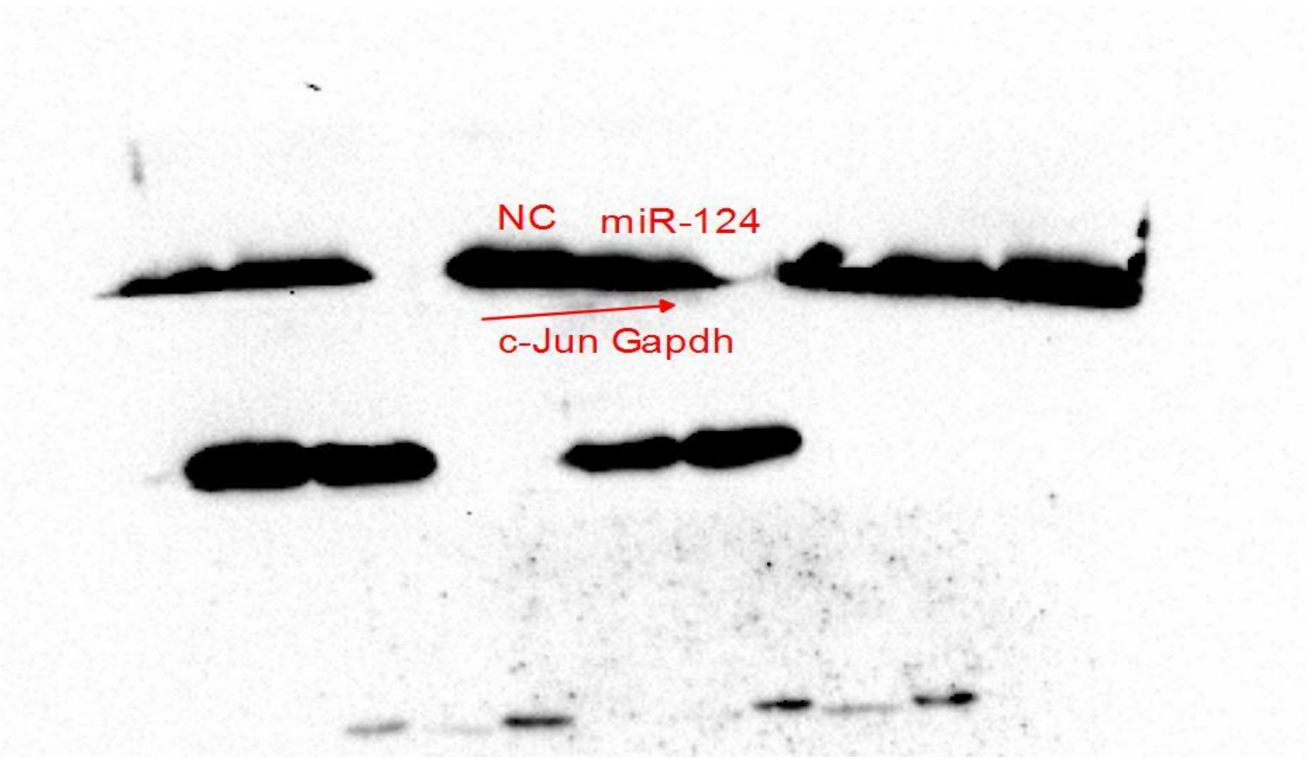
NC
miR-124
c-Jun Gapdh

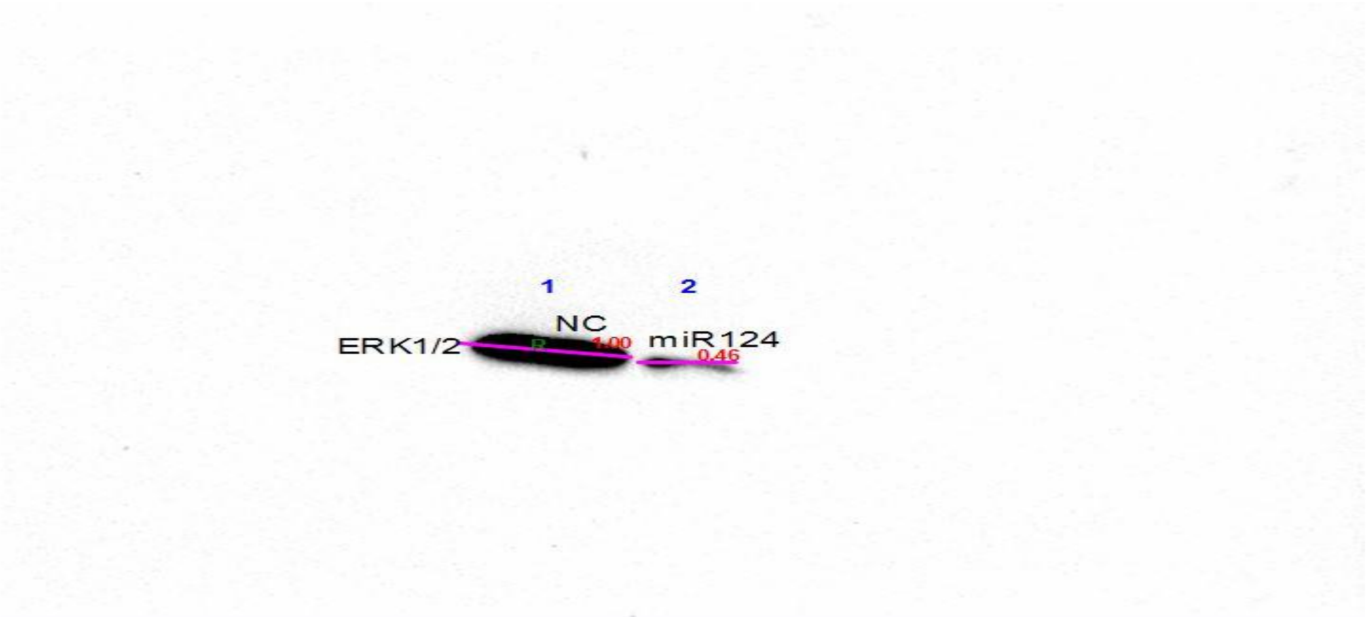
1
2
NC
ERK1/2
1.00
miR124
0.46

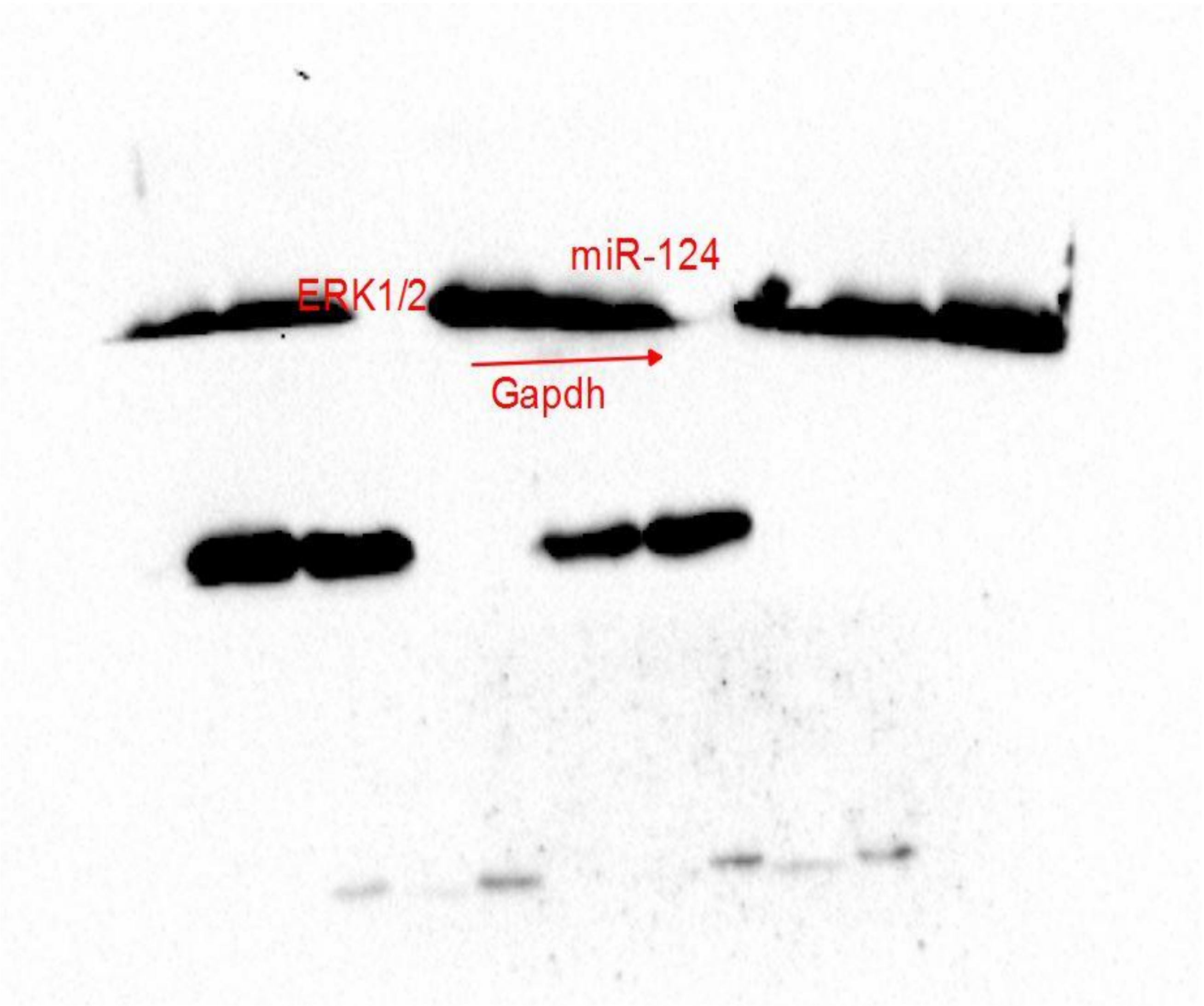
miR-124
ERK1/2
Gapdh

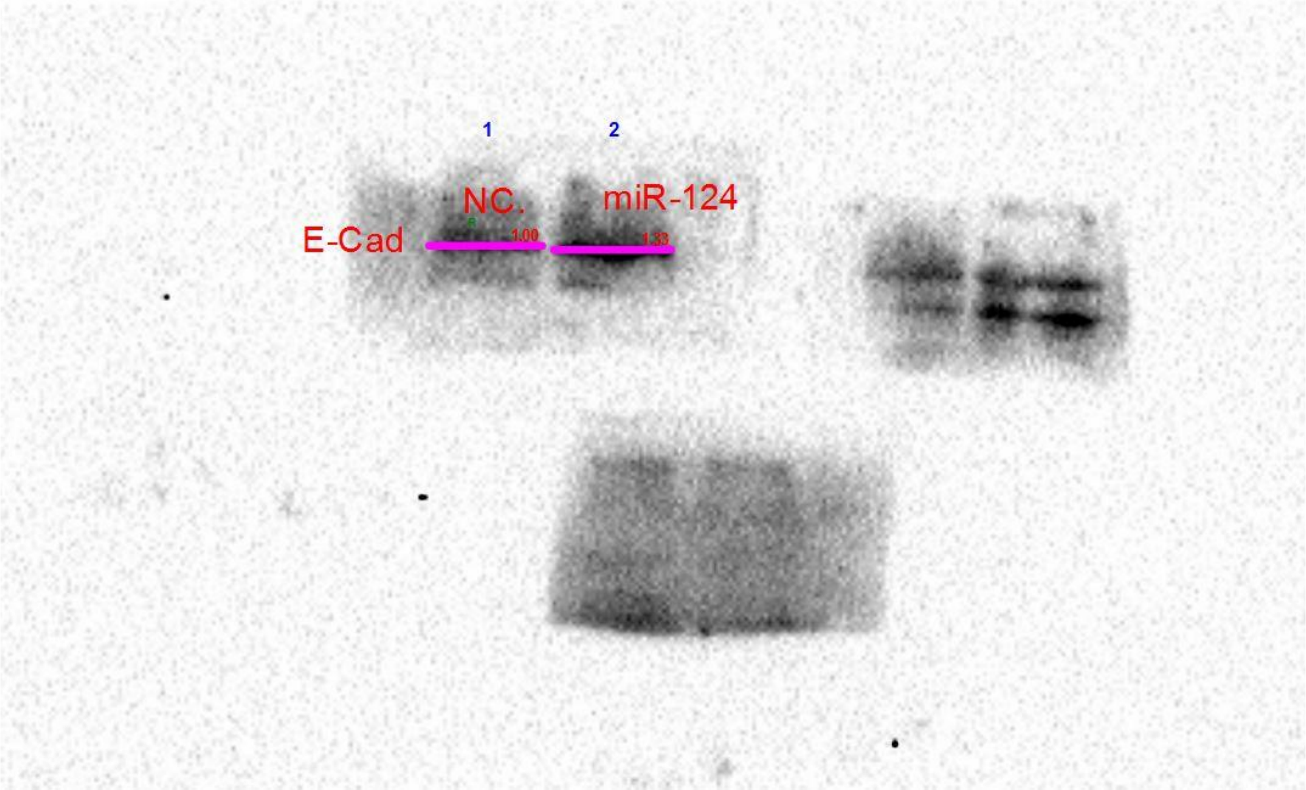
1
2
NC.
miR-124
E-Cad
1.00
1.33

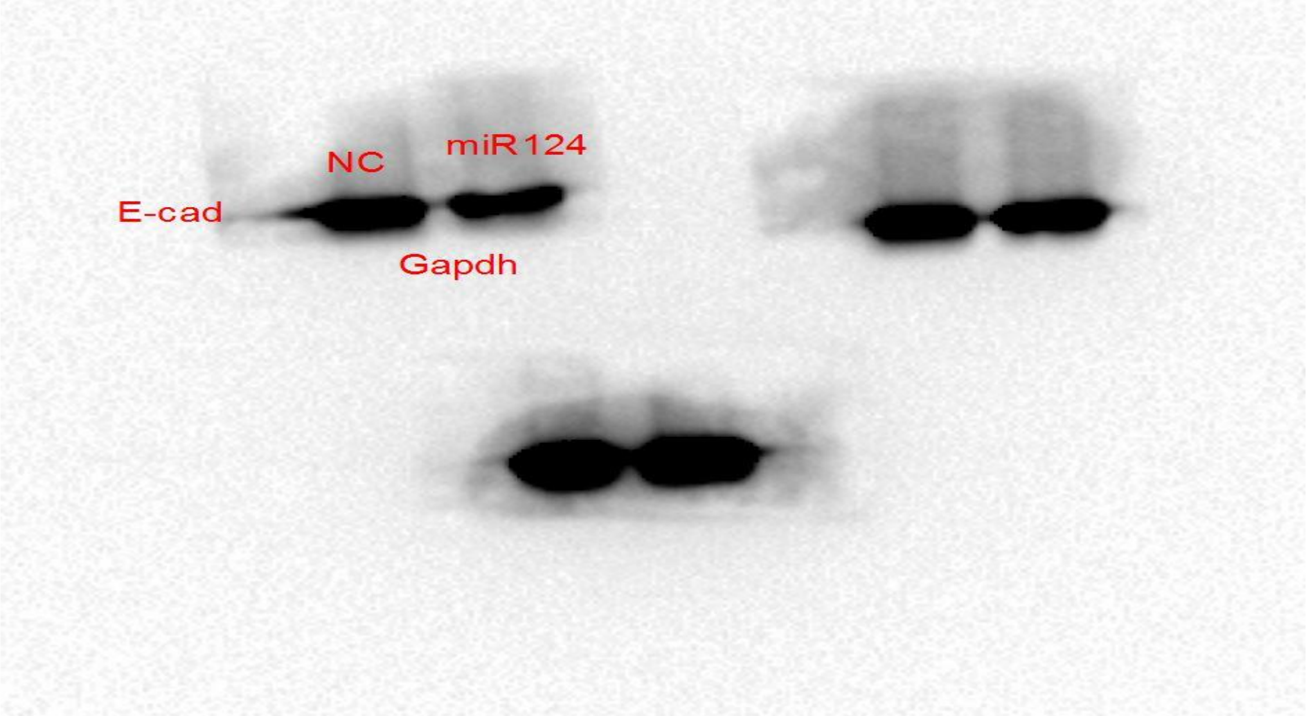
NC
miR124
E-cad
Gapdh

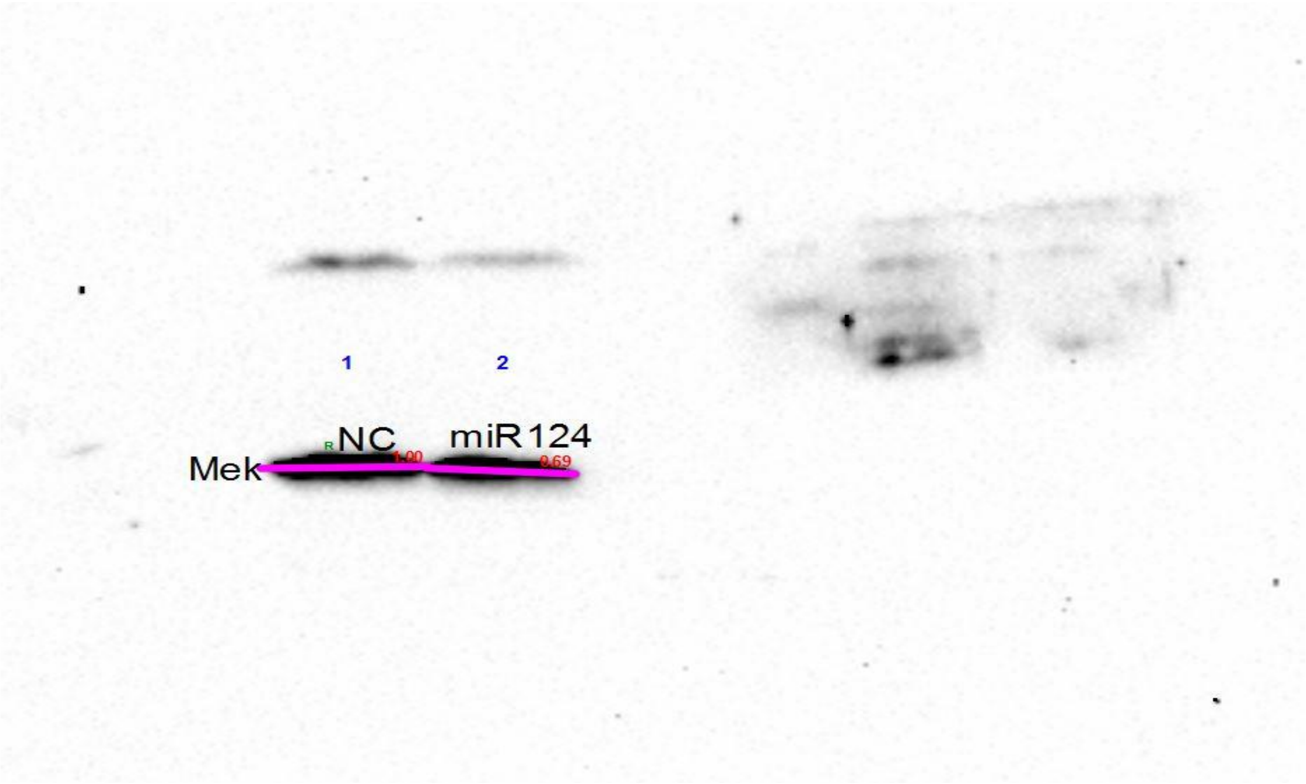
1
2
NC
miR124
Mek
1.00
0.69

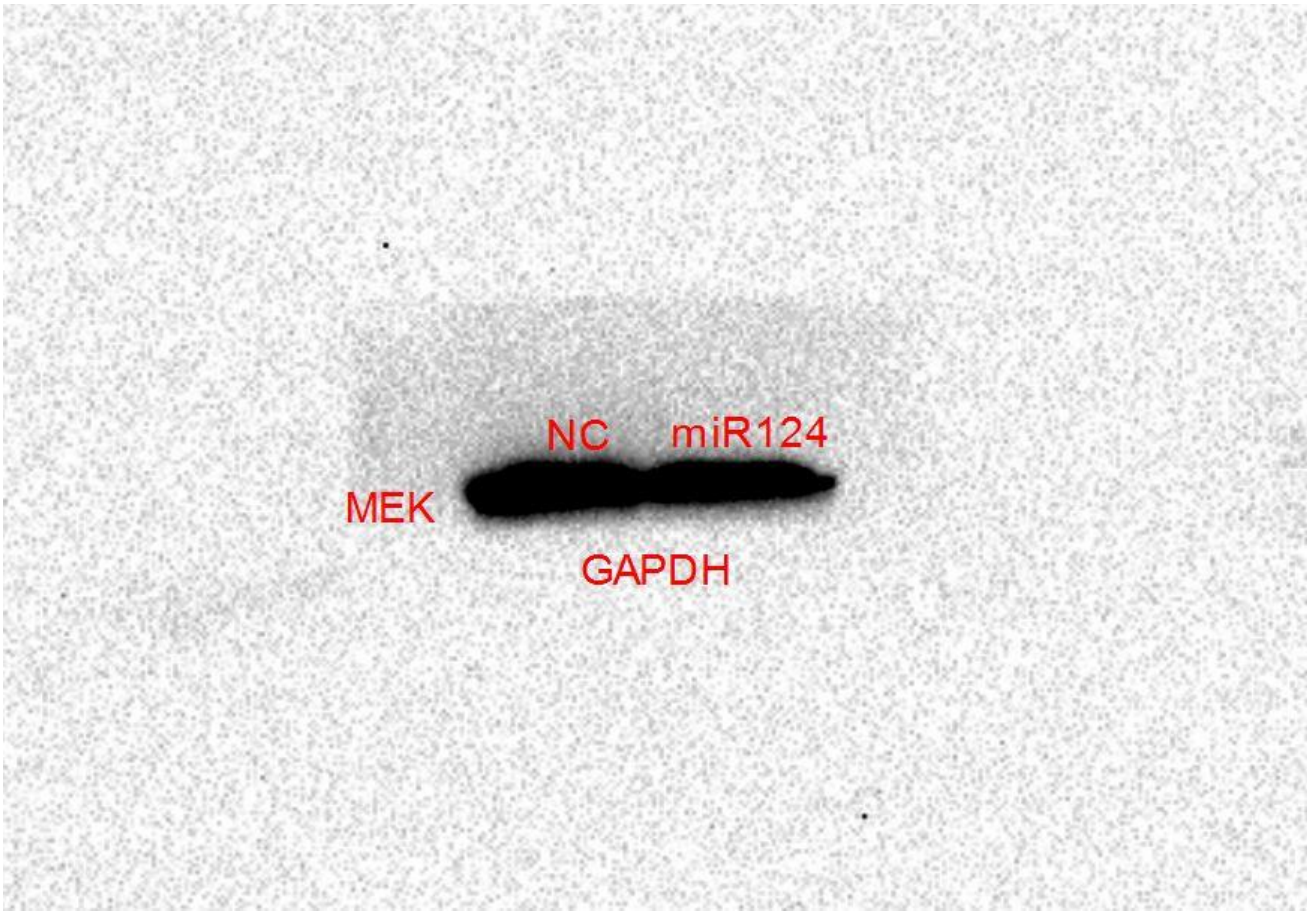
NC
miR124
MEK
GAPDH

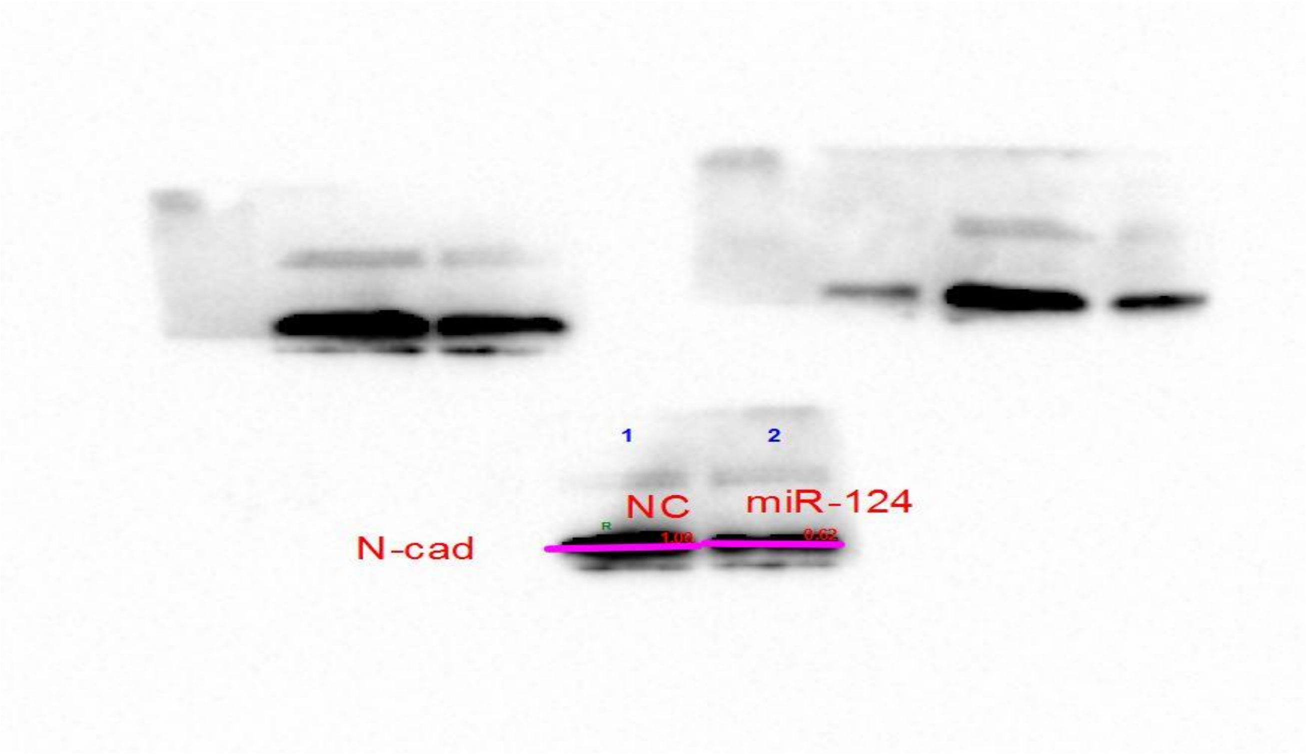
1
2
NC
miR-124
N-cad

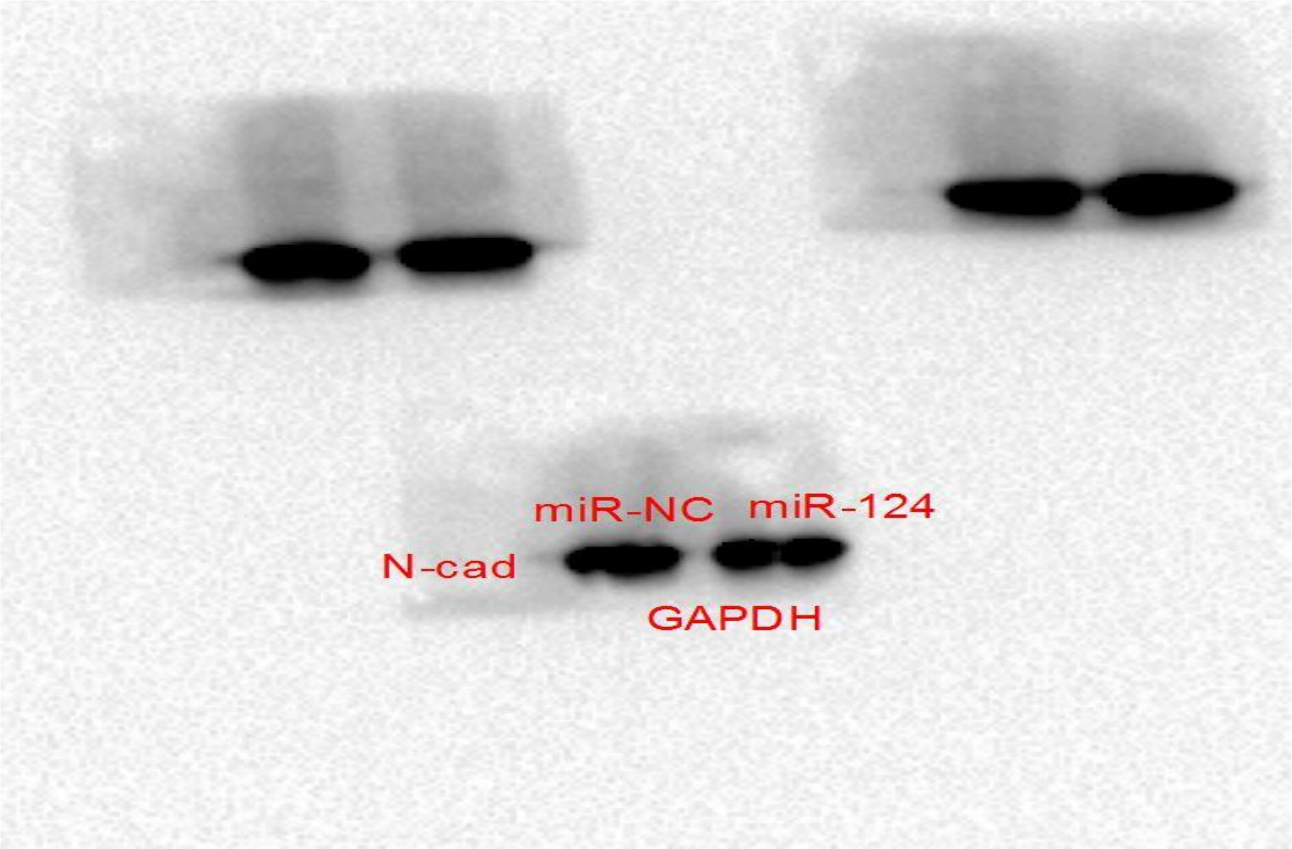
miR-NC
miR-124
N-cad
GAPDH

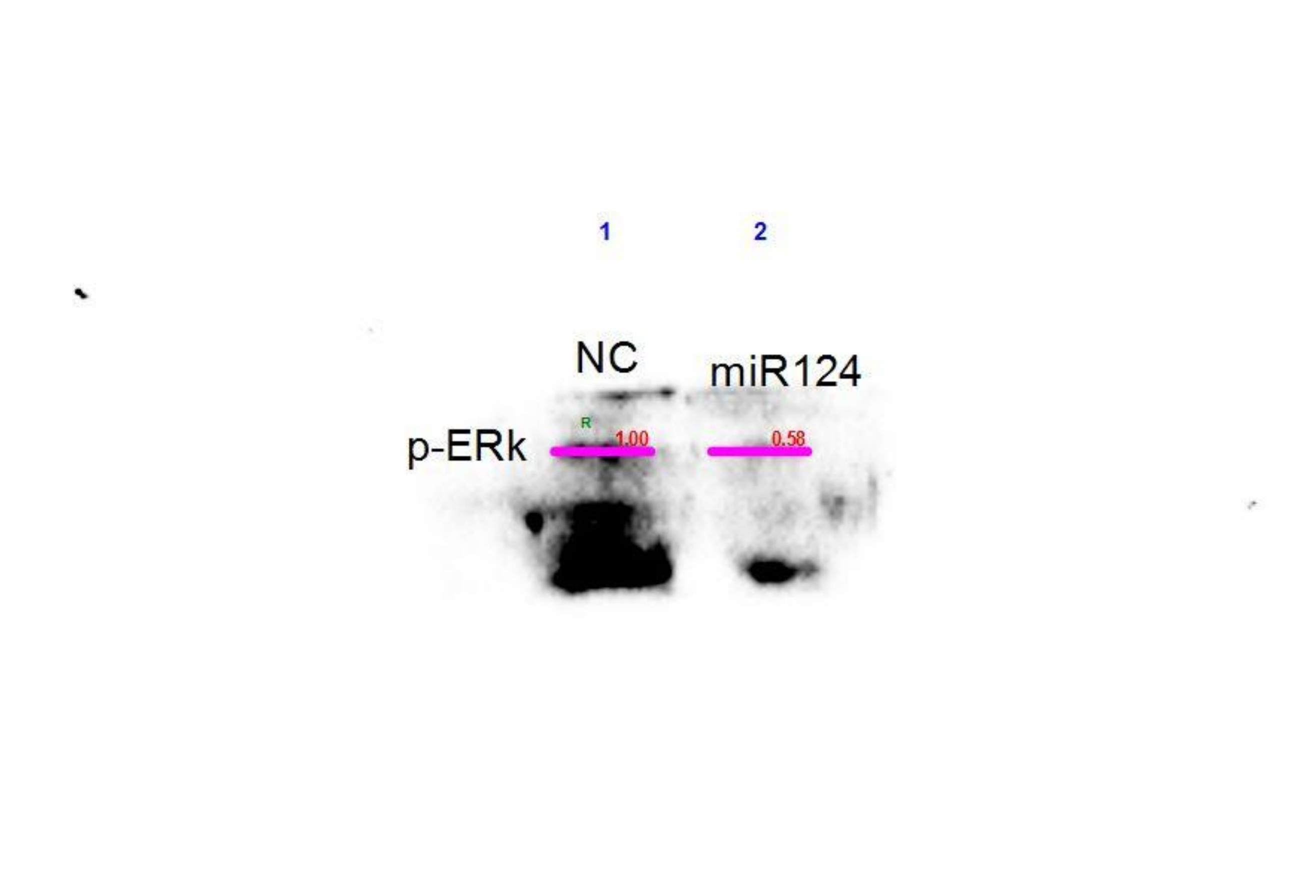
1
2
NC
miR124
p-ERk
1.00
0.58

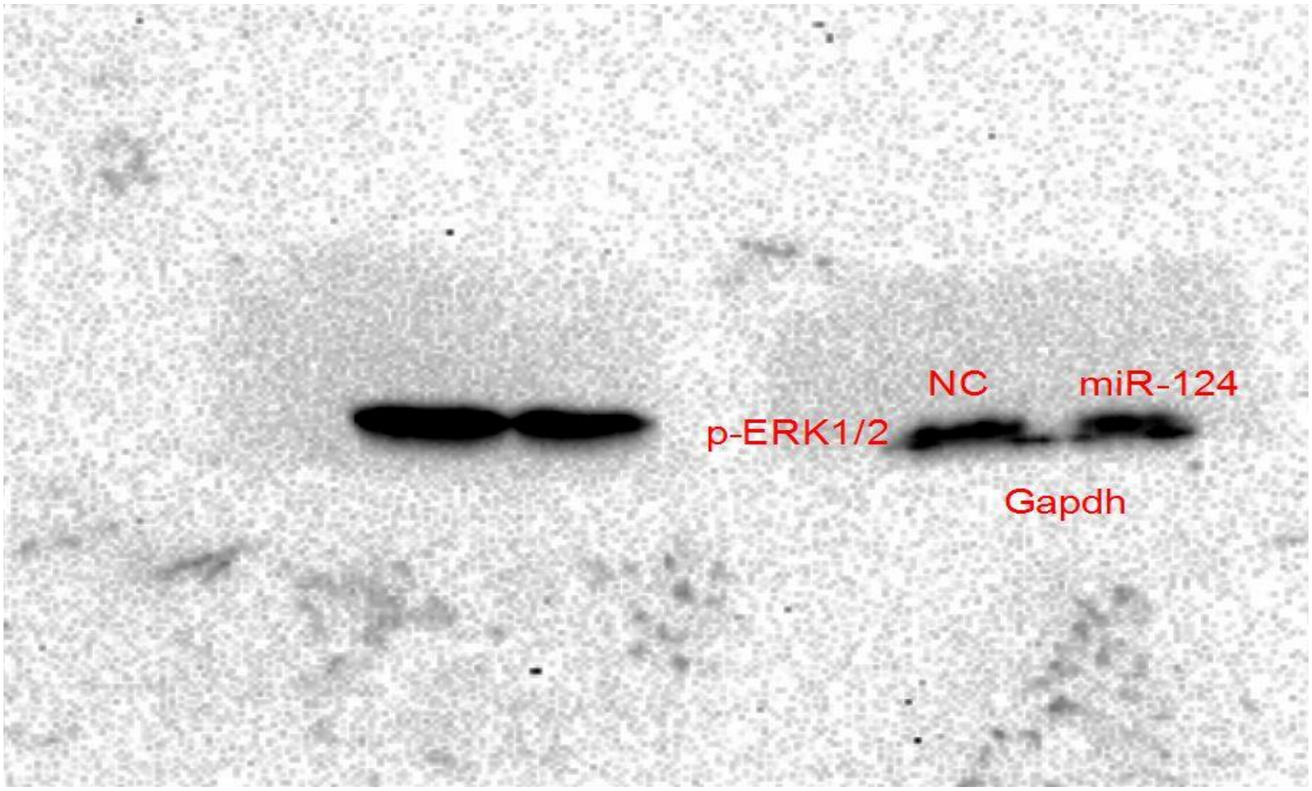
NC
miR-124
p-ERK1/2
Gapdh

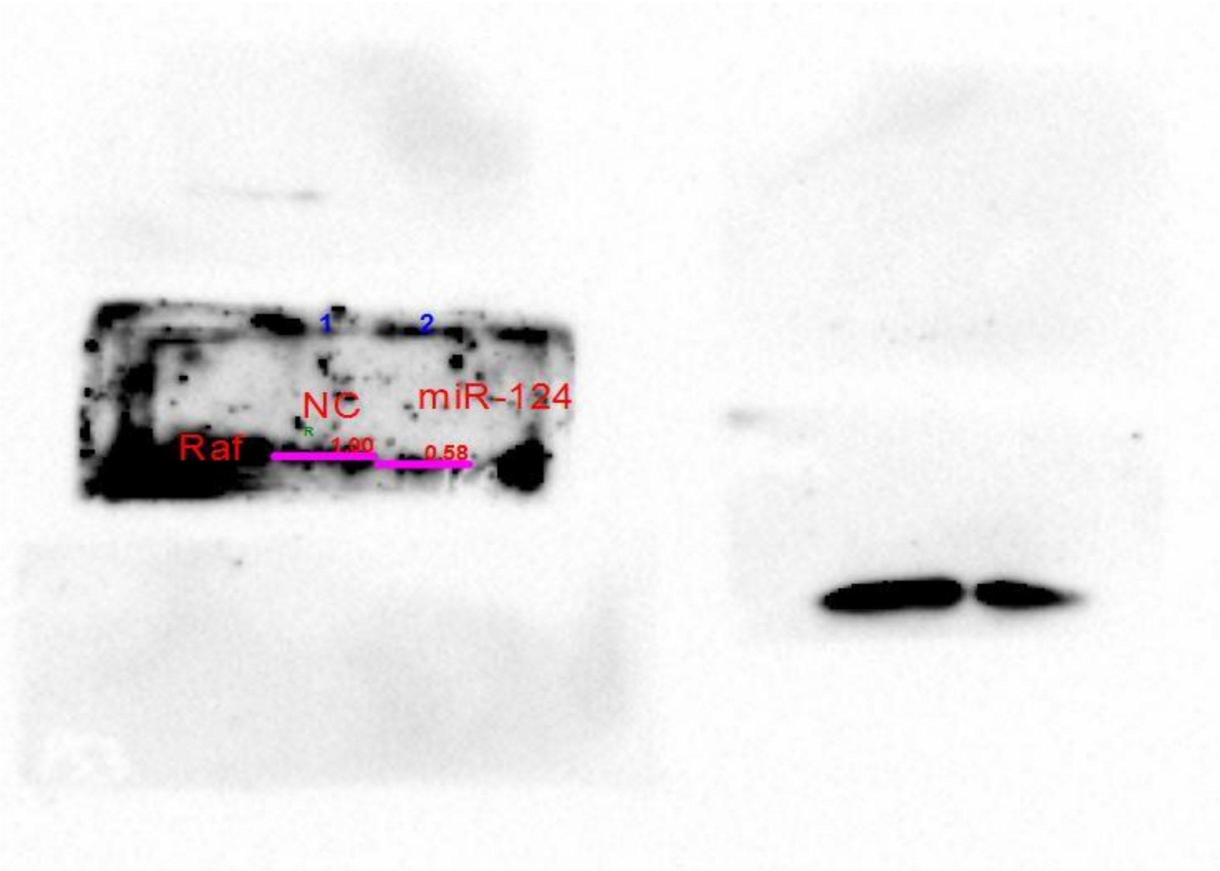
1
2
NC
miR-124
Raf
1.00
0.58

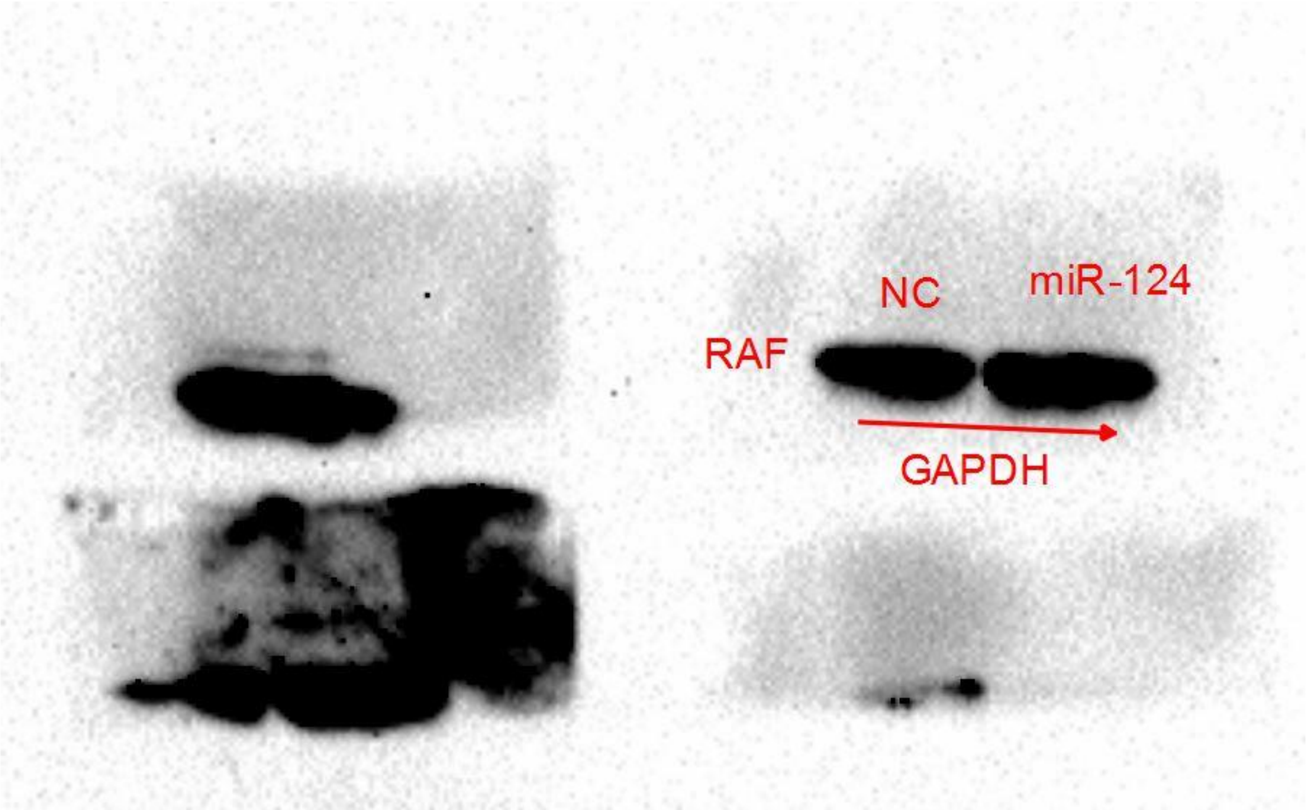
NC
miR-124
RAF
GAPDH

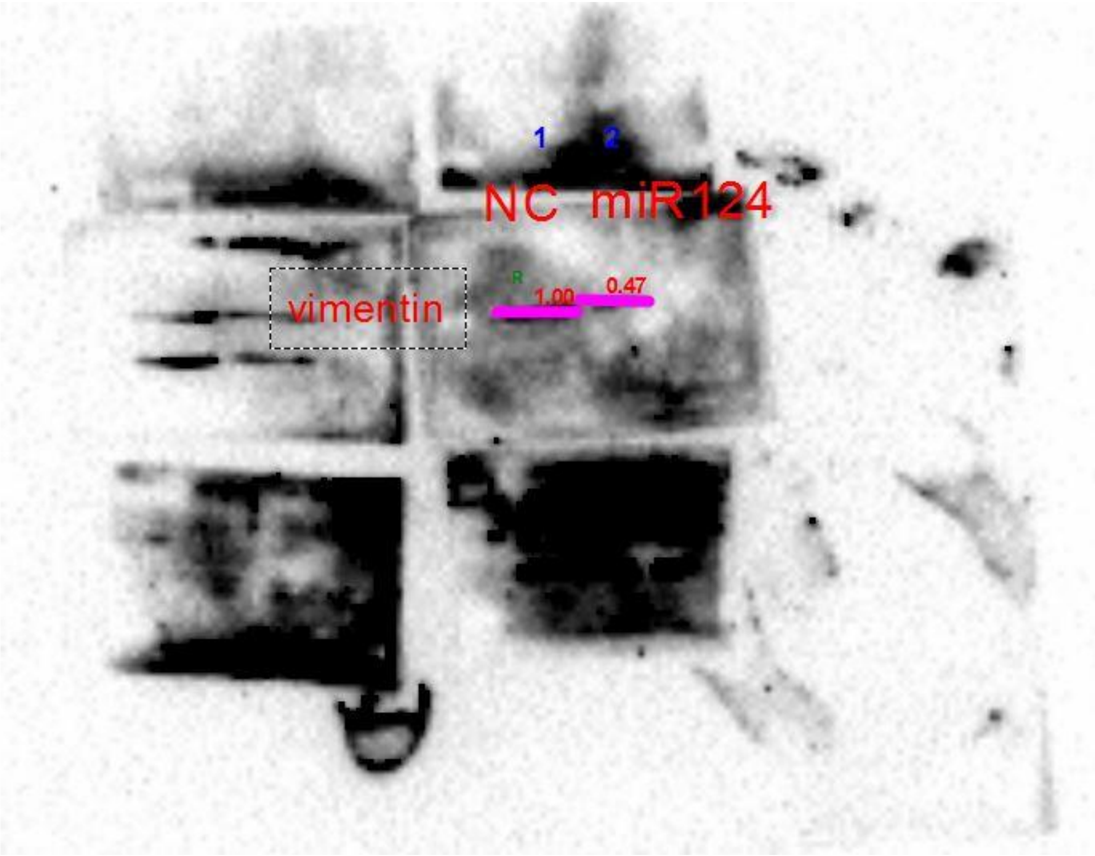
1
2
NC miR124
vimentin
1.00
0.47

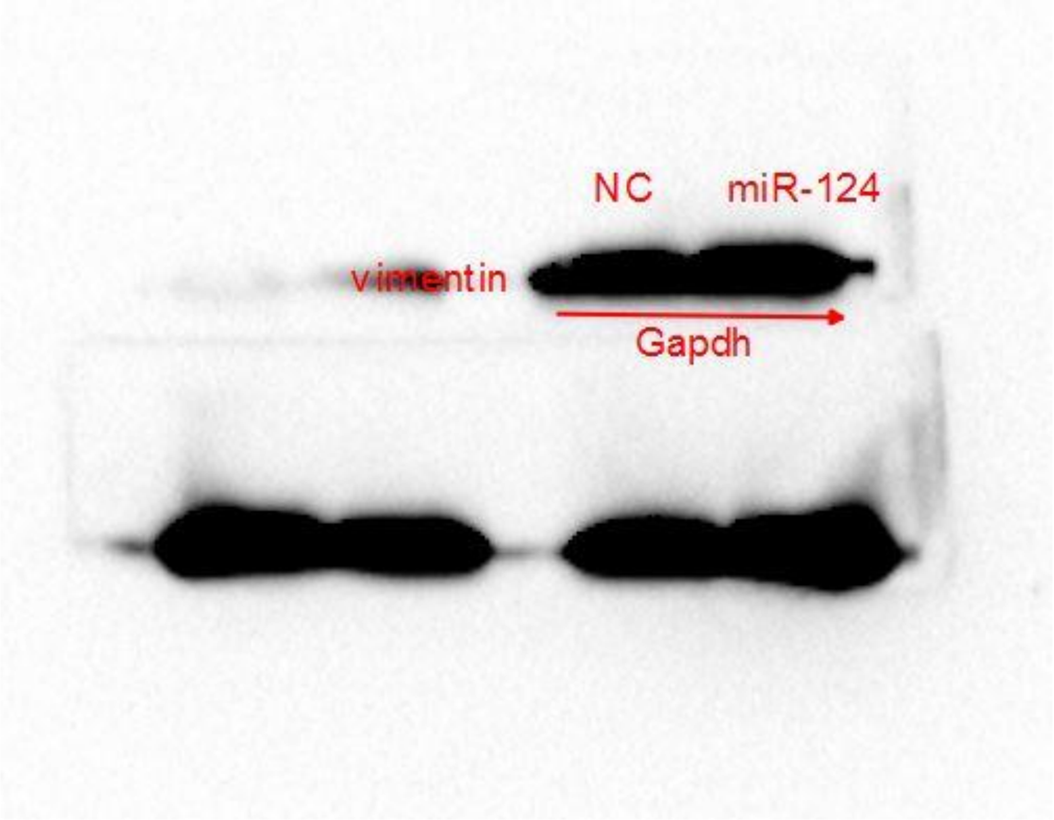
NC
miR-124
vimentin
Gapdh

Figure 5 original Blots of Bax, BCL-2, C-JUN, E-cad, ERK1/2, MEK, N-cad, p-ERK1/2, Raf and vimentin transfected with miR-NC and miR-124-3p mimics in Huh7**.**

2 Supplementary Tables

2.1 Supplementary table 1.

Vector Primer sequences

No	Name	Primers	Sequences
1	psiCHECK-2-CRKL-3′-UTR-WT	Forward	5'-CTCGAGGCTCATAGTGAACACAGCAGACCTAGAAATGTAGC-3'
		Reverse	5'-GCGCGGCCGCCGGCTGATGCAAGTTTTATTGAGACAATAT-3'
2	Site 1	Forward	5’- CTCGAGATTAGCCCTTCCTTGCCAGTGAGAAC -3’
		Reverse	5’- GCGGCCGCAGTCCGTTAAGTTTTACTAGTTT-3’
3	Site 2	Forward	5’-CTCGAGGGGATGATGTGGTTTTTTGCCAGGTGTTTATAAT-3’
		Reverse	5’-GCGGCCGCCGGCTGATGCAAGTTTTATTGAGACAATAT -3’
4	psiCHECK-2-CRKL-3′-UTR-MUT1 (Set 1)	Forward	5' CTCGAGATTAGCCCTTCCTTGCCAGTGAGAAC -3'
		Reverse	5'-GGTCGTGCCCTCCTTGAACTTCCGTGACATTCCTCCC -3'
5	psiCHECK-2-CRKL-3′-UTR-MUT1 (Set 2)	Forward	5'-CAGAGGGAGGAATGTACAGGAAGTTCAAGGAGGGCACG -3'

		Reverse	5'-CTCGAGGGGATGATGTGGTTTTTGCCAGGTGTTTATAAT -3'
6	psiCHECK-2-CRKL-3′-UTR-MUT2 (Set3)	Forward	5'-CTCGAGGGGATGATGTGGTTTTTGCCAGGTGTTTATAAT-3'
		Reverse	5'-GCGGCGGCGGAATACAAAAATAAATTCCGTGTAATCTGTT -3'
7	psiCHECK-2-CRKL-3′-UTR-MUT2 (Set4)	Forward	5'-CTTAACAGATTACACGGAATTTATTTTTGTAT -3
		Reverse	5'-CGGCTGATGCAAGTTTTATTGAGAC-3'

2.2 Supplementary Table 2.

MTT Analysis of miR-124-3p transfected HCCLM3 cells compared with miR-NC

LM3	exp1	exp2	exp3	mean	SD	p value	%
means of all 24 hours experiment NC	0.104	0.1092	0.1058	0.10633333	0.002641		
mean of all 24 hours experiment mimic	0.1054	0.092	0.1006	0.09933333	0.006789	ns	
mean of all 48 hours experiment NC	0.1446	0.1468	0.1658	0.1524	0.011657		
mean of all 48 hours experiment mimic	0.122	0.1268	0.1372	0.12866667	0.00777	*P=0.0426	15.58%
mean of all 72 hours experiemtn NC	0.1894	0.1876	0.2146	0.1972	0.015096		
mean of all 72 hours experiemtn mimic	0.153	0.1472	0.1616	0.15393333	0.007245	*P=0.0110	21.95%
mean of all 96 hours experiment NC	0.2738	0.3082	0.341	0.30766667	0.033603		
mean of all 96 hours experiment mimic	0.1972	0.1904	0.248	0.21186667	0.031477	*P=0.0227	31.14%

Supplementary Table 3

MTT Analysis of miR-124-3p transfected Huh7 cells compared with miR-NC

HUH7

	exp1	exp2	exp3	mean	SD	p value	%
means of all 24 hours experiment NC	0.1018	0.1076	0.1004	0.10326667	0.003818		
mean of all 24 hours experiment mimic	0.095	0.0892	0.0924	0.0922	0.002905	*P=0.0162	10.72%
mean of all 48 hours experiment NC	0.1178	0.1168	0.117	0.1172	0.000529		
mean of all 48 hours experiment mimic	0.1056	0.103	0.1024	0.10366667	0.001701	***P=0.0002	11.45%
mean of all 72 hours experiemtn NC	0.1368	0.1452	0.1446	0.1422	0.004686		
mean of all 72 hours experiemtn mimic	0.1148	0.1206	0.1186	0.118	0.002946	**P=0.0016	17.02%
mean of all 96 hours experiment NC	0.2366	0.2404	0.2494	0.24213333	0.006574		
mean of all 96 hours experiment mimic	0.1704	0.1824	0.1842	0.179	0.007502	***P=0.0004	26.08%
